# Squaric Ester-Based, pH-Degradable Nanogels: Modular Nanocarriers for Safe, Systemic Administration of Toll-like Receptor 7/8 Agonistic Immune Modulators

**DOI:** 10.1021/jacs.1c03772

**Published:** 2021-06-24

**Authors:** Anne Huppertsberg, Leonard Kaps, Zifu Zhong, Sascha Schmitt, Judith Stickdorn, Kim Deswarte, Francis Combes, Christian Czysch, Jana De Vrieze, Sabah Kasmi, Niklas Choteschovsky, Adrian Klefenz, Carolina Medina-Montano, Pia Winterwerber, Chaojian Chen, Matthias Bros, Stefan Lienenklaus, Niek N. Sanders, Kaloian Koynov, Detlef Schuppan, Bart N. Lambrecht, Sunil A. David, Bruno G. De Geest, Lutz Nuhn

**Affiliations:** ∥ Max Planck Institute for Polymer Research, 55128 Mainz, Germany; ‡ Institute for Translational Immunology and Research Center for Immune Therapy, University Medical Center, Johannes Gutenberg-University Mainz, 55131 Mainz, Germany; ¶ Department of Internal Medicine I, University Medical Center of the Johannes Gutenberg-University Mainz, 55131 Mainz, Germany; † Department of Pharmaceutics and Cancer Research Institute Ghent (CRIG), Ghent University, Ghent 9000, Belgium; Δ Department of Internal Medicine and Pediatrics, Ghent University, VIB Center for Inflammation Research, Ghent 9052, Belgium; § Laboratory of Gene Therapy, Department of Nutrition, Genetics and Ethology, Ghent University, Merelbeke 9820, Belgium; ° Department of Dermatology, University Medical Center of Johannes Gutenberg-University Mainz, 55131 Mainz, Germany; $ Institute for Laboratory Animal Science and Institute of Immunology, Hannover Medical School, 30625 Hannover, Germany; ∇ Division of Gastroenterology, Beth Israel Deaconess Medical Center, Harvard Medical School, Boston, Massachusetts 02215, United States; ⊥ Department of Pulmonary Medicine, Erasmus University Medical Center, Rotterdam 3015, Netherlands; # ViroVax, LLC, Lawrence, Kansas 66047, United States

## Abstract

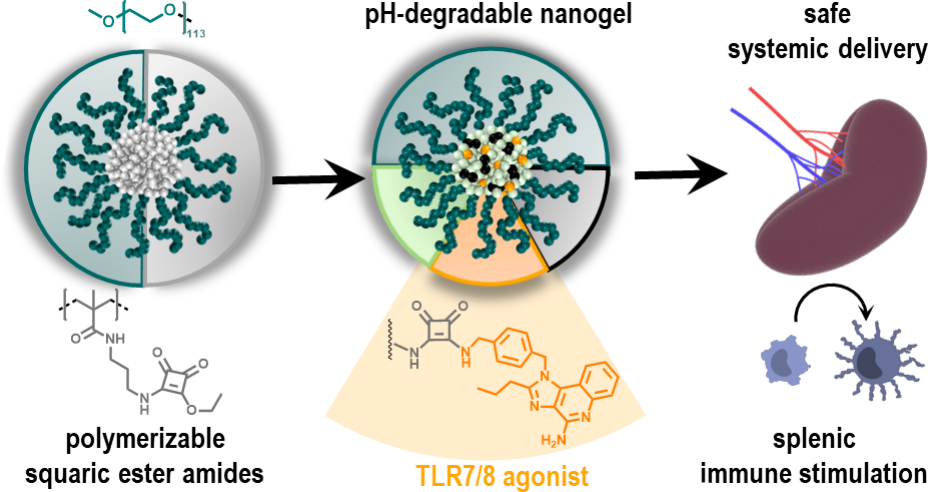

Small-molecular Toll-like receptor 7/8 (TLR7/8) agonists hold promise as immune modulators for a variety of immune therapeutic purposes including cancer therapy or vaccination. However, due to their rapid systemic distribution causing difficult-to-control inflammatory off-target effects, their application is still problematic, in particular systemically. To address this problem, we designed and robustly fabricated pH-responsive nanogels serving as versatile immunodrug nanocarriers for safe delivery of TLR7/8-stimulating imidazoquinolines after intravenous administration. To this aim, a primary amine-reactive methacrylamide monomer bearing a pendant squaric ester amide is introduced, which is polymerized under controlled RAFT polymerization conditions. Corresponding PEG-derived squaric ester amide block copolymers self-assemble into precursor micelles in polar protic solvents. Their cores are amine-reactive and can sequentially be transformed by acid-sensitive cross-linkers, dyes, and imidazoquinolines. Remaining squaric ester amides are hydrophilized affording fully hydrophilic nanogels with profound stability in human plasma but stimuli-responsive degradation upon exposure to endolysosomal pH conditions. The immunomodulatory behavior of the imidazoquinolines alone or conjugated to the nanogels was demonstrated by macrophages *in vitro*. *In vivo*, however, we observed a remarkable impact of the nanogel: After intravenous injection, a spatially controlled immunostimulatory activity was evident in the spleen, whereas systemic off-target inflammatory responses triggered by the small-molecular imidazoquinoline analogue were absent. These findings underline the potential of squaric ester-based, pH-degradable nanogels as a promising platform to permit intravenous administration routes of small-molecular TLR7/8 agonists and, thus, the opportunity to explore their adjuvant potency for systemic vaccination or cancer immunotherapy purposes.

## Introduction

Nanosized drug carriers can improve the pharmacokinetics of potent but systemically toxic small-molecular drugs; however, key challenges remain to establish robust fabrication processes, prevent premature drug release, and guarantee sufficient carrier integrity under physiological conditions. Moreover, opportunities toward on-demand drug release and carrier degradation would be desired.^1−4^ Polymer-based nanocarriers can be designed to address these challenges chemically, especially for applications in immunotherapy.^5,6^ A spatiotemporal control over recently identified, highly potent immunomodulators is crucial, since their drug leakage can cause uncontrolled systemic immune responses combined with immense off-target toxicity.^7,8^ Covalent conjugation of immunomodulators to polymer-based nanocarriers that control the drug’s pharmacokinetic profile can pave the way to safer systemic applications in cancer immunotherapy or as adjuvants during vaccination.^9−11^

One promising class of immunomodulators are agonists of Toll-like receptor 7/8 (TLR7/8) that usually sense viral single-stranded RNA and raise an immediate cellular innate immune response.^12^ Imidazoquinolines, a class of synthetic TLR7/8 agonists, are particularly powerful in stimulating these receptors. They can induce the maturation of antigen-presenting cells and the release of cytokines that promote cellular immune responses, especially via activation of T helper 1 (Th1) and cytotoxic T cells.^13,14^ The latter play a key role in combating cancer and intracellular pathogens.^15,16^ As TLR7/8 are located at the endosomal membrane of phagocytosing innate immune cells, they can be preferentially targeted by nanocarriers equipped with imidazoquinoline-derived TLR7/8 agonists.^17^

Previous studies demonstrated that undesired systemic inflammation by the highly potent small-molecular TLR7/8 agonist 1-(4-(aminomethyl)benzyl)-2-butyl-1*H*-imidazo[4,5-*c*]quinolin-4-amine (IMDQ)^18^ can be circumvented through its covalent conjugation to different types of nanocarriers. Thereby, its activity upon subcutaneous administration can be restricted to the site of injection and the draining lymph nodes. A variety of nanocarriers has been investigated for this objective including lipids,^19,20^ polysaccharides,^21^ synthetic polymers,^22^ micelles,^23^ and nanogels.^24,25^ These nanocarrier formulations have generally been injected subcutaneously and provided a local TLR7/8 activation by lymph node trafficking. However, a few findings revealed that intravenous injections would result in even improved immune responses.^26−29^ Especially during intravenous nanoparticle vaccination, more stem-like antigen-specific CD8^+^ T cells are generated that lead to superior antitumor responses, especially during immune checkpoint inhibition therapy.^30^ Yet, systemic treatments set new requirements for safety and allow for new targeting strategies as compared to local administration. Thus, new efforts have to be made in the design of novel blood-stable nanocarriers enabling intravenous and organ-targeted TLR7/8 agonist administration.

A straightforward way to covalently load IMDQ into nanocarriers is self-assembled reactive ester block copolymers that are converted by amidation reactions into pH-degradable nanogels.^24,25,31^ Via controlled radical polymerization processes we have previously generated poly(triethylene glycol monomethyl ether methacrylate)-*block*-poly(pentafluorophenyl methacrylate) as precursor polymers for which the pentafluorophenyl esters trigger both micellar self-assembly in polar aprotic solvents such as DMSO and selective reactivity toward primary amines.^32,33^ This could also be applied for conjugation of IMDQ and subsequent transformation into fully hydrophilic nanogels. However, for this approach the hydrophilic triethylene glycol monomethyl ether methacrylate block renders a lower critical solution temperature in water,^34^ which can impair the required shielding properties after exposure to biological media. Additionally, the applied pentafluorophenyl ester exhibits only limited hydrolytic resistance. Already slight traces of water can risk its hydrolysis into methacrylic acid^35^ and, thus, impede access to the nanogel formation process. In addition, during amidation toxic pentafluorophenol is released,^36^ which requires careful purification prior to applications *in vitro* or *in vivo*.

Consequently, alternative amine-reactive groups with higher hydrolytic stability and nontoxic byproducts are needed. Tietze et al. reported on squaric alkyl esters for selective amine coupling reactions with high functional group tolerance as well as excellent yields, even under mild conditions in aqueous media.^37^ Due to their homo-bifunctionality squaric alkyl esters can consecutively be aminolyzed by two different amines, whereby an enhanced aromatic stability after first amidation is obtained, providing reduced reactivity for another amidation. Thus, it assures a controlled sequential squaric bisamide formation.^38^ Compared to other reactive esters (including pentafluorophenyl) this alternative linking chemistry allows conjugation in biocompatible solvents (e.g., water, ethanol) while releasing alcohols as nontoxic byproducts.^39^

**Figure 1:**
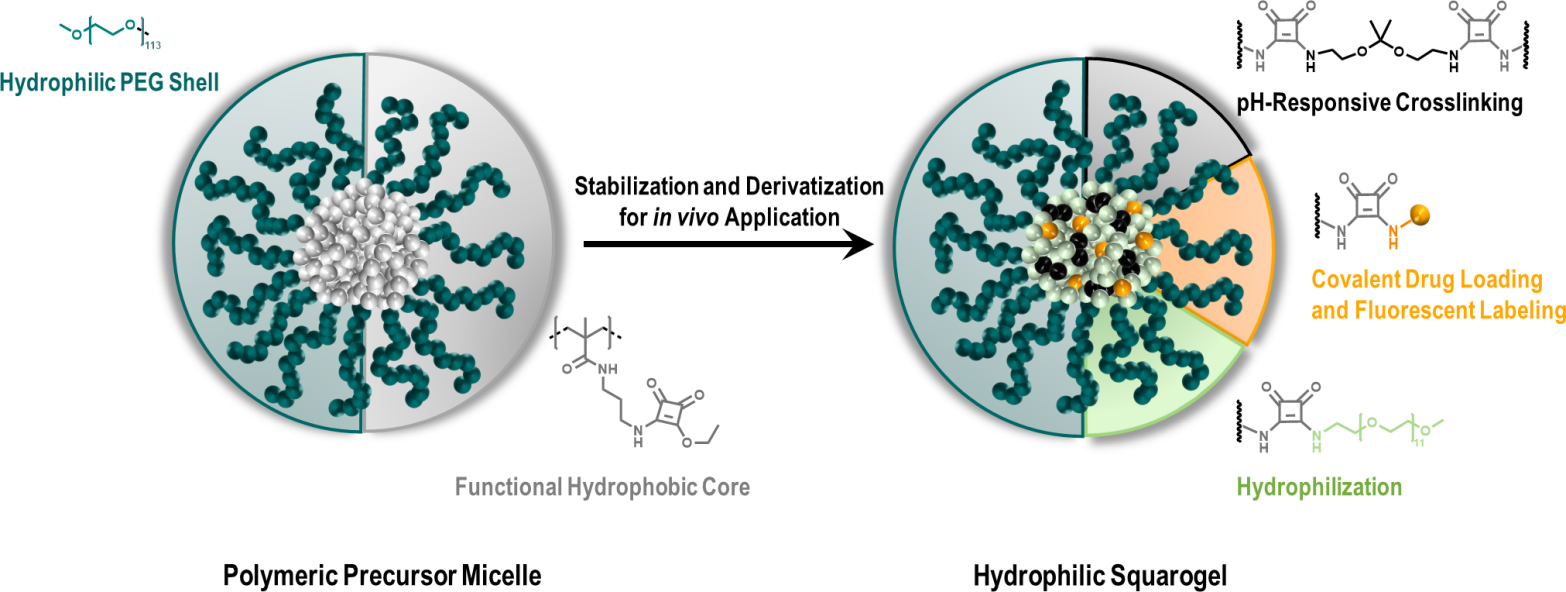
Squaric ester-based nanogels derived from polymeric precursor micelles formed by self-assembly of squaric ester amide-containing amphiphilic block copolymers. Subsequent transformation for *in vivo* application is achieved by amidation of pendant squaric ester amide groups inside the amine-reactive, hydrophobic core including pH-responsive cross-linking, covalent drug or dye loading, and hydrophilization, affording fully hydrophilic drug-loaded nanogels.

In this study, we therefore introduce polymerizable squaric ester amides for a more robust and hydrolysis-resistant postpolymerization modification of pH-degradable, immunodrug-loaded nanogels. Methacrylamides with pendant squaric ester amides are grafted under controlled radical polymerization conditions onto linear hydrophilic PEG chains and serve as solvophobic groups for spontaneous micellar self-assembly now also in polar protic solvents (Figure 1).

By sequential functionalization with primary amines, they can be converted into a modular pH-responsive nanogel platform. This process supports covalent conjugation of both hydrophobic and hydrophilic drugs into the core of these nanogels, e.g., the amine-functionalized TLR7/8 agonist IMDQ. Due to its high degree of PEGylation, this system features excellent stability in blood that permits intravenous administration and local maturation of antigen-presenting immune cells inside the spleen, while circumventing systemic inflammatory responses caused by the unbound TLR7/8 agonist. Altogether, these observations demonstrate the broad versatility of amine-reactive squaric ester-based precursor polymers and how they provide access to a modular pH-degradable nanogel platform for safe systemic administration of immune modulators.

## Results and Discussion

We first aimed to synthesize a polymerizable squaric ester amide monomer that can be polymerized under controlled conditions into well-defined homo- and block copolymers using reversible addition–fragmentation chain transfer (RAFT) agents. While squaric ester amides have already been studied as amine-reactive groups for atom transfer radical polymerization (ATRP)^40^ or RAFT^41^ chain transfer agents, to the best of our knowledge, the use of squaric esters as functional side groups of monomers and corresponding polymers has not been introduced before. They provide access to a new class of hydrolysis-resistant, amine-reactive precursor polymers for multiple postpolymerization purposes.^39^ We investigated the polymer’s reactivity toward different types of amines and observed a self-assembly behavior of corresponding block copolymers in water or ethanol. The core of the resulting micelles can be used to ligate fluorescent dyes or IMDQ followed by core-cross-linking and hydrophilization affording pH-degradable nanogels. Subsequent *in vitro* and *in vivo* characterization revealed long blood circulation and maturation of antigen-presenting cells in the spleen while omitting inflammatory off-target effects.

### Monomer and Polymer Syntheses

**Figure 2:**
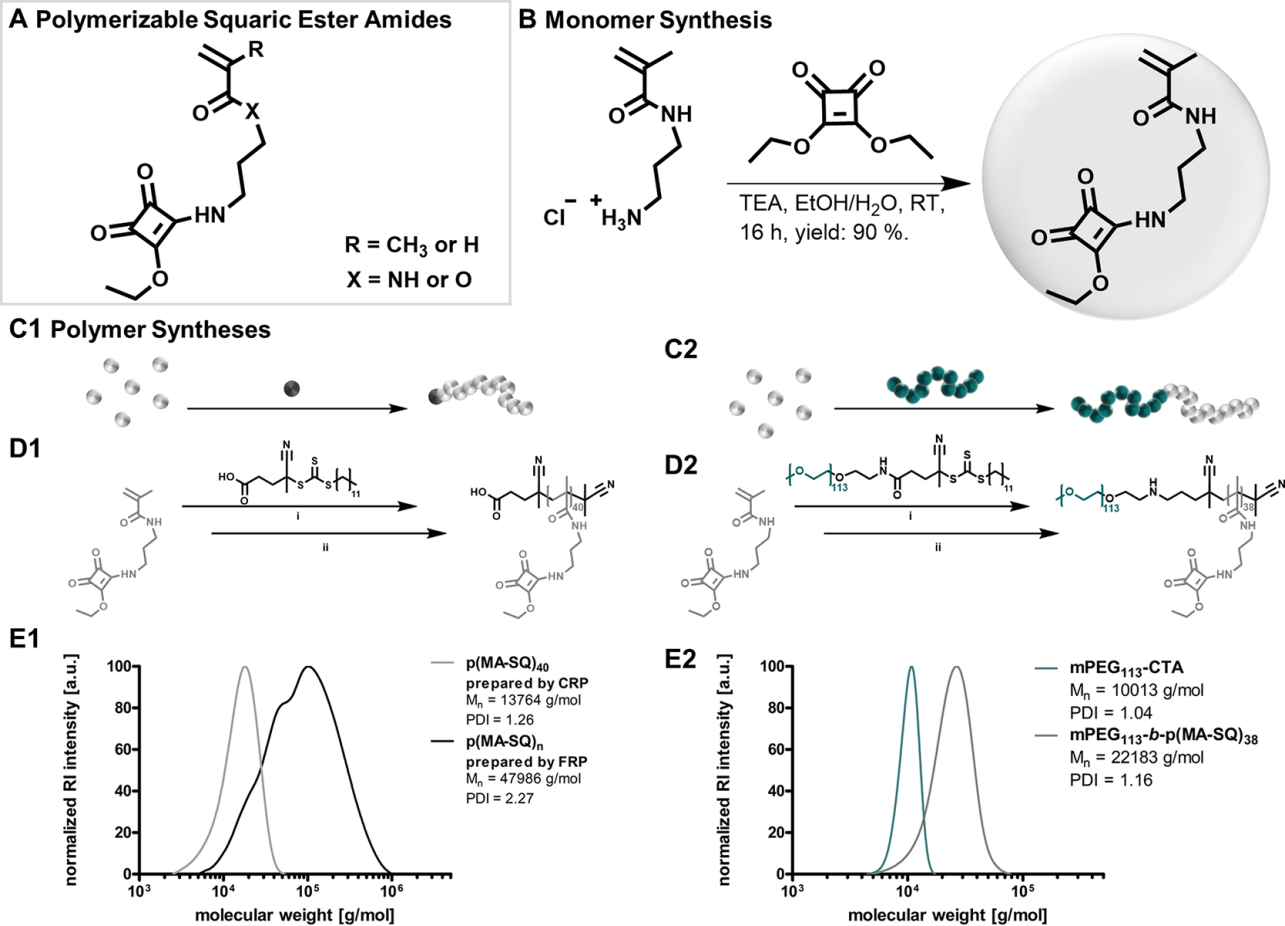
Synthesis of polymerizable squaric ester amides. (A) General chemical structure of synthesized monomers with a pendant squaric ester amide group. (B) Synthesis route toward the most suitable monomer squaric ester amide methacrylamide (MA-SQ). (C) Schematic illustrations and (D) synthesis schemes of RAFT homopolymerization of MA-SQ with a small molecular trithiocarbonate chain transfer agent (TTC-CTA) (C1 and D1) and block copolymerization with macro-chain-transfer agent PEG-TTC-CTA (C2 and D2) (reaction conditions: (i) DMF, 70 °C, 0.2 equiv AIBN per 1.0 equiv TTC-CTA, and (ii) DMF, 70 °C, 50 equiv AIBN (to remove the TTC end group)). (E) Molecular weight distributions of polymers obtained by radical polymerization: (E1) RAFT-derived homopolymer compared to the homopolymer obtained by FRP; (E2) RAFT-derived block copolymer compared to PEG-TTC-CTA as macro-chain-transfer agent.

Initially, polymerizable squaric ester amide monomers with acrylate, acrylamide, or methacrylamide groups were synthesized (Figure 2A). Direct (meth-)acryloylation of 2-aminoethanol-functionalized squaric ester amides failed due to occurring bisacryloyl derivatives and autopolymerization. For instance, we carefully treated 3-ethoxy-4-((2-hydroxyethyl)amino)cyclobut-3-ene-1,2-dione with acryloyl chloride but obtained besides the desired squaric ester amide acrylate product 2-((2-ethoxy-3,4-dioxocyclobut-1-en-1-yl)amino) ethyl acrylate (At-SQ) also a bisacrylated species (At-SQ-At) at similar yields, as documented in the Supporting Information (Figures S14–S23). We therefore opted for (meth-)acrylamides by controlled monoamidation of squaric acid diethyl ester with amine-functionalized (meth-) acrylamides. Unfortunately, due to their aza-Michael reactivity, amino-functionalized (meth-)acrylamides are only stable as protonated salt species. While the corresponding methacrylamide-based monomer was commercially available, we synthesized the TFA salt of *N*-(3-aminopropyl)acrylamide as corresponding acrylamide derivative (Supporting Information Figure S7). It was converted with squaric acid diethyl ester catalyzed by triethylamine in ethanol and water and provided the desired acrylamide monomer at acceptable yields (64%, A-SQ, NMR characterization in Supporting Information Figures S10–S13). However, several attempts to polymerize it, even under free radical polymerization (FRP) conditions, failed or yielded only conversions below 30%. Fortunately, synthesis and polymerization of the methacrylamide analogue was more promising. Monoamidation of squaric acid diethyl ester with the commercially available *N*-(3-aminopropyl)methacrylamide hydrochloride was again conducted in ethanol and water catalyzed by triethylamine. It afforded a squaric ester amide methacrylamide monomer (MA-SQ) with a high yield of 90% (Figure 2B, NMR and MS characterization in Supporting Information, Figures S1–S6).

MA-SQ could afterward be polymerized under FRP conditions at 70 °C using AIBN as radical source with quantitative conversion. Consequently, it was an ideal candidate for RAFT polymerization. We chose a trithiocarbonate as chain transfer agent (TTC-CTA) and polymerized MA-SQ with either a small-molecular TTC-CTA or a PEG-functionalized macro-TTC-CTA (Supporting Information Figure S24–S26; note that instead of commercially available macro-TTC-CTAs bearing an ester group between PEG and the CTA group, we preferred to apply an amide derivative to avoid possible ester aminolysis and cleavage during amidation). At a monomer to CTA ratio of 50:1, conversions of 75–80% were found affording p(MA-SQ)_40_ as homopolymer and PEG_113_-*b*-p(MA-SQ)_38_ as block copolymer (Figure 2C/D).

Consistent with earlier reports, NMR analysis validated that squaric ester amide moieties withstood radical polymerization conditions and remained intact (Figures S28 and S32).^40,41^ Molecular weight distributions of p(MA-SQ)_40_ obtained by size-exclusion chromatography (SEC) revealed well-defined homopolymers with a number-average molar mass (*M*_n_) of 13 800 g/mol and polydispersity index (PDI) of 1.26 (Figure 2E1). In stark contrast to the FRP, the RAFT homopolymers of MA-SQ confirmed controlled polymerization conditions. Via NMR spectroscopy, monomer conversion over time demonstrated first-order kinetics for the polymer formation (Figures S34 and S35). Similar conditions were applied during block copolymerization and provided polymers with a narrow PDI of 1.16 and *M*_n_ of 22 200 g/mol (Figure 2E2). The molecular weight shift of the block copolymer (PEG_113_-*b*-p(MA-SQ)_38_) compared to PEG-TTC-CTA clearly attested full MA-SQ monomer grafting onto the macro-CTA. Additionally, DOSY NMR analysis confirmed successful block copolymer formation by providing identical diffusion coefficients of both blocks (Figure S29). To avoid interference with the trithiocarbonate (TTC) end group during subsequent amidation of the squaric ester amides, both homo- and block copolymer were further treated with an excess of AIBN for end group removal. This additional step did not impair the composition or distribution of the polymers (compare Supporting Information Figures S30 and S33).

### Amine-Selective Conversion

**Figure 3:**
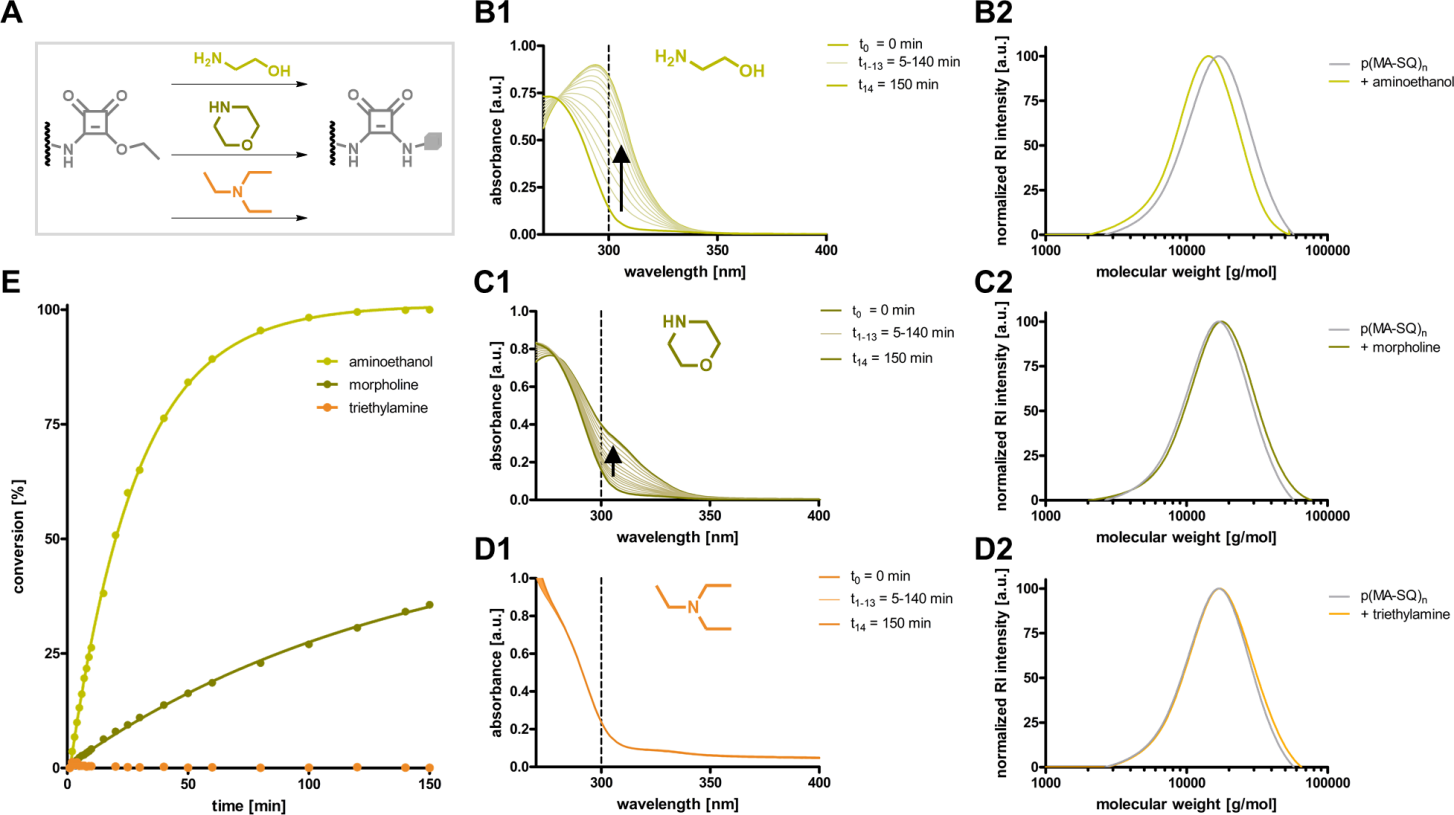
Amine selectivity of pendant squaric amide groups of p(MA-SQ)_40_. (A) Scheme for the conversion of polymeric squaric ester amides to squaric bisamides by amidation with aminoethanol, morpholine, or triethylamine. UV/vis spectra during conversion with (B1) aminoethanol, (C1) morpholine, or (D1) triethylamine and the corresponding molecular weight distributions of p(MA-SQ)_40_ before and after conversion with (B2) aminoethanol, (C2) morpholine, or (D2) triethylamine. (E) Corresponding conversions estimated by UV absorbance over time.

In order to verify amine reactivity, the homopolymer p(MA-SQ)_40_ was first reacted with an excess of primary, secondary, or tertiary amine. As model compounds aminoethanol, morpholine, and triethylamine were selected (Figure 3A, note that these reactions were conducted in DMSO to ensure complete solubility of the homopolymer). Interestingly, the formed squaric bisamides provide an absorption maximum around 300 nm compared to the squaric ester amide educts. Therefore, their aminolysis can easily be monitored over time by UV/vis absorbance spectroscopy.^39^ A strong and fast increase in absorbance at 300 nm revealed rapid and quantitative conversion of the squaric ester amides by the primary amine aminoethanol. The secondary amine morpholine could only convert about 25% after 2 h, while no conversion was observed with triethylamine as tertiary amine (Figure 3B1–D1, Figure 3E, and Supporting Information Figure S37). The respective homopolymers were isolated by precipitation in diethyl ether. Recorded molecular weight distributions by SEC demonstrated no significant change in *M*_n_ and PDI for p(MA-SQ)_40_ treated with triethylamine, while a complete peak shift to smaller molecular weights was found after conversion with aminoethanol (Figure 3B2–D2). The squaric ester amide polymers treated with morpholine showed only a minimal peak shift reflecting their incomplete conversion. These observations were confirmed by NMR analysis (Figures S38 and S39). Further studies indicated that full conversion by secondary amine morpholine can still be achieved at elevated temperature and extended reaction time (Figure S40, e.g., after 22 h at 70 °C complete squaric ester amide aminolysis was demonstrated; compare Figures S41 and S42). These results highlight the suitability of squaric ester amides as reactive polymer side groups for quantitative postpolymerization modification with preferentially fast conversion of primary amines.

### Nanogel Fabrication and Characterization

**Figure 4:**
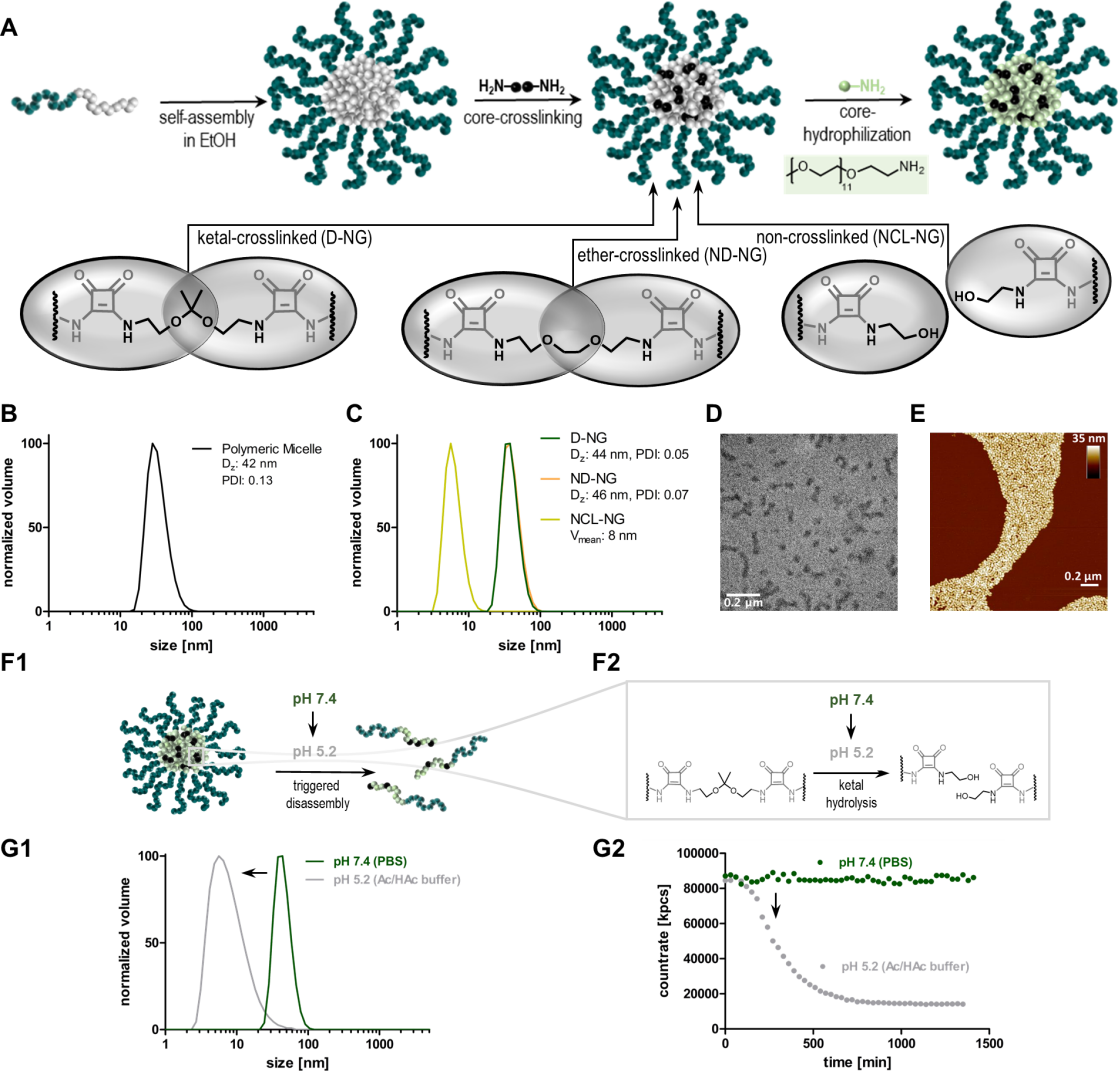
Nanogel fabrication process. (A) Scheme of sequential nanogel formation from squaric ester amide-derived, self-assembling precursor polymers. Based on the cross-linking strategy, degradable nanogels (D-NG) as well as nondegradable or non-cross-linked control samples (ND-NG, NCL-NG) can be obtained. (B) DLS size distribution of self-assembled micelles in ethanol and (C) fully fabricated nanogels D-NG, ND-NG, and NCL-NG in PBS. (D) TEM image of D-NG. (E) AFM image of D-NG. (F1 and F2) Scheme of ketal-hydrolysis in acidic media resulting in D-NGs' disassembly into soluble unimers (NCL-NG). (G) DLS study of D-NG at neutral pH in PBS compared to mildly acidic pH in HAc/Ac buffer by (G1) size distribution and (G2) DLS count rate over time.

Polymeric squaric ester amides can be used as precursors to introduce new functionalities by amidation reactions. Following the design concept of Figure 4A, we were interested in whether this postpolymerization modification can also be combined with self-assembly to sequentially access nanogels (by micelle formation, covalent core-cross-linking, and transformation of the core from hydrophobic to hydrophilic). The well-defined amphiphilic block copolymer PEG_113_-*b*-p(MA-SQ)_38_ was suspended in ethanol and treated with ultrasound, yielding narrowly dispersed micelles. By dynamic light scattering (DLS) their sizes were analyzed, revealing a *z*-average diameter (*D*_*Z*_) of 44 nm and a narrow PDI of 0.13 (Figure 4B). Next, the polarity of the core was switched chemically by exploiting its reactivity toward hydrophilic amines. After addition of 5.0 equiv of PEG_11_-amine the micelles disassembled within 30 min and provided fully soluble polymers (Supporting Information Figures S43 and S44; note that amines like aminoethanol did not increase the polarity to unfold the micelles). Thus, core hydrophilization is possible to fabricate fully hydrophilic nanogels.

Next, polymeric micelles were modified by pH-responsive cross-links prior to hydrophilization (note that the following process is possible in both pure ethanol and buffered water; compare Supporting Information Figure S50). In relation to 1.0 equiv of pendant squaric ester amide moieties, 0.15 equiv of the ketal-containing bisamine 2,2′-bis(aminoethoxy)propane was added. Its ketal unit renders the nanocarriers’ sensitivity to endosomal acidic pH. As shown in previous reports, it can effectively hydrolyze at pH 5, resulting in a disassembly of nanogels into soluble polymer chains.^33,42,43^ In order to provide a nondegradable nanogel version, the core of the micelles was conjugated with 0.15 equiv of an ether-containing bisamine (1,2-(bis(aminoethoxy)ethane). Additionally, a non-cross-linked soluble polymer representing the ketal-cross-linked nanogel after hydrolysis was obtained by aminolysis with 0.3 equiv of 2-aminoethanol. For each species, all remaining squaric ester amide moieties were finally converted with 3.0 equiv of PEG_11_-amine, resulting in fully hydrophilic nanogel or soluble polymer species (Figure 4A). All samples were purified by dialysis against water containing 0.1 wt % ammonia to prevent premature ketal-hydrolysis. Subsequent lyophilization afforded the nanogels as dry powders, which could be easily redispersed in PBS buffer.

UV/vis absorbance spectroscopy could again be used to monitor the conversion of the squaric esters inside the self-assembled micelles/core-cross-linked nanogels by following the increase of the absorption maximum around 300 nm related to the formed squaric bisamides (compare Supporting Information Figures S46–S48). Sequential addition of primary amines for functionalization, cross-linking, and final conversion of the remaining squaric ester amide moieties with PEG_11_-amine yielded a stepwise increase of the absorbance. Note that no difference between the absorption after addition of 3.0 equiv of PEG_11_-amine and the purified nanogel after extensive dialysis against water with 0.1 wt % ammonia could be found, confirming quantitative squaric ester conversion inside the nanogels during the fabrication process (compare Supporting Information Figures S46–S48).

As shown by DLS analysis, the ketal-cross-linked, degradable nanogel (D-NG) showed a very narrow monomodal distribution and a *D*_*z*_ of 44 nm, which was similar to the size of the precursor micelle. The same was found for the nondegradable nanogel (ND-NG, *D*_*z*_ = 46 nm), while for the non-cross-linked version (NCL-NG) a significantly lower count rate was detected indicating the absence of particular scattering. The average volume diameter of the NCL-NG was 8 nm (Figure 4C). The ketal-cross-linked nanogels were imaged by transmission electron microscopy (TEM) and atomic force microscopy (AFM). The dried particles could be recorded as spherical and some slightly elongated morphologies with narrow dispersities (Figure 4D and E; Supporting Information Figure S49). To prove that the ketal-cross-linked nanogels fully disassemble into soluble unimers under endolysosomal pH conditions (Figure 4F1,F2), several DLS stability studies at pH 7.4 or 5.2 were performed. D-NG was exposed to acidic pH in acetate buffer at 10 mg/mL and subsequently monitored over time by DLS. A gradual decline in count rate within 600 min was observed, while a decrease of the particles’ size distribution demonstrated complete nanogel disassembly into single polymer chains (Figure 4G1,G2). In contrast, no change in count rate or size was observed at neutral pH for D-NG in PBS (Figure 4G1,G2). This was also the case for ND-NG and NCL-NG at both pH levels over time (Supporting Information (Figure S54). Thus, ketal-cross-linked nanogels represent ideal candidates for drug delivery due to their transient supramolecular architecture that unfolds into secretable smaller entities upon exposure to endolysosomal pH values.

### Nanogel Functionalization

**Figure 5:**
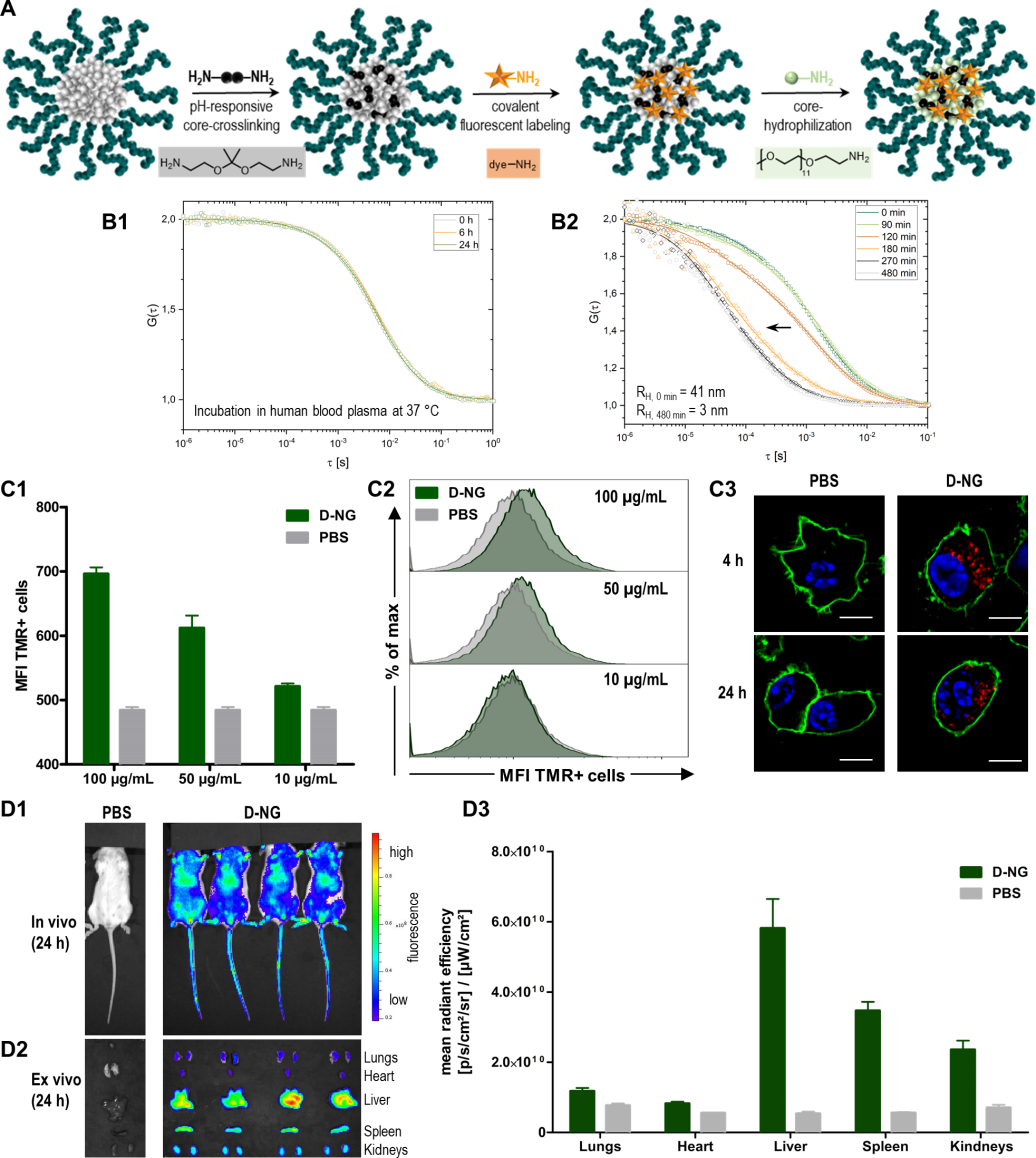
Fluorescently labeled nanogels provide sufficient particle integrity for safe *in vivo* administration. (A) Scheme of sequential nanogel fabrication with *in situ* installed fluorescent dye-labeling. (B) Oregon Green cadaverine (OG)-labeled nanogels (D-NG) investigated by fluorescence correlation spectroscopy toward stability in plasma and the ability to disassemble upon exposure to endolysosomal pH values: (B1) normalized autocorrelation curves (dots) and corresponding fits (solid line) of OG-labeled D-NG incubated in human blood plasma for 0, 6, and 24 h; (B2) normalized autocorrelation curves (dots) and corresponding fits (solid line) of OG-labeled D-NG incubated in acidic buffer (pH 5.2) over time. (C) Tetramethylrhodamine cadaverine (TMR)-labeled nanogels (D-NG) investigated for the *in vitro* uptake behavior in RAW murine macrophages using flow cytometry and confocal microscopy imaging (*n* = 3): (C1) cellular mean fluorescence intensities (MFI) of RAW macrophages incubated with TMR-labeled D-NG at 100, 50, and 10 μg/mL for 24 h; (C2) corresponding histograms of RAW macrophages incubated with TMR-labeled D-NG at 100, 50, and 10 μg/mL for 24 h (*n* = 3) or PBS (control). (C3) Confocal microscopy images of RAW macrophages incubated with TMR-labeled D-NG at 100 μg/mL for 4 and 24 h (blue: nuclei stained with Hoechst 33258; green: cell membrane stained with cholera toxin B (CTB)-AF488; red: TMR-labeled D-NG, scale bar 10 μm). (D) Near infrared dye 800RS cadaverine (NIR)-labeled nanogels (NIR-labeled D-NG) investigated for their biodistribution after systemic application to BALB/c mice via tail vein injection using an *in vivo* NIR-imaging system (IVIS): (D1) whole body fluorescence imaging 24 h after intravenous injection of 100 μL of NIR-labeled D-NG dispersion (2 mg/mL); (D2) *ex vivo* organ distribution imaging and (D3) semiquantitative analysis (*n* = 4).

To further investigate the nanogels’ covalent drug delivery function, their squaric ester amides were first used to introduce fluorescent labels. Prior to the described cross-linking and core-hydrophilization, 0.01 equiv of amine-bearing fluorescent dyes (e.g., Oregon Green cadaverine (OG), tetramethylrhodamine cadaverine (TMR), or the near-infrared dye 800RS cadaverine (NIR)) was conjugated to the core (Figure 5A). Covalent dye conjugation did not affect the nanogel fabrication process. To ensure the absence of free dye, nanogels were extensively spin-filtrated (MWCO: 10 000 g/mol) using a mixture of 0.1 wt % ammonia in water/EtOH (v/v, 1:2) in addition to the described purification methods. Dye labeling enables analyses of the nanocarriers’ behavior in complex biological environment and, thus, provides key features to evaluate their applicability for immunomodulating drug delivery.

We first investigated the dye-labeled nanogel’s stability in human blood plasma as complex biological medium relevant for *in vitro* and *in vivo* application. Selective monitoring of nanogel integrity over time was facilitated by fluorescence correlation spectroscopy (FCS). It provides information about the nanocarrier’s size in solution by autocorrelation analysis of fluorescence intensity fluctuations that result from diffusional motions of fluorescent species (e.g., labeled nanocarriers) through a confocal volume.^44^ After incubation of pH-degradable nanogels (OG-labeled D-NG) in human blood plasma for 0, 6, and 24 h at 37 °C, FCS analysis confirmed that the nanogel’s autocorrelation curve and the corresponding hydrodynamic radius remained identical (Figure 5B1). Consequently, the carriers are profoundly stable under these biologically relevant conditions: Neither an increase of the nanogel’s size, which would reflect aggregation with serum components nor its decrease was observed, which would imply nanogel degradation. The latter could only be observed by FCS when the ketal-cross-linked OG-labeled D-NG was again exposed to pH 5.2 using acetate buffer. As evident from the shift of the autocorrelation curves, one can perfectly observe a sequential disassembly of the nanogels into single unimers (Figure 5B2).

Next, cellular nanogel interaction was studied for TMR-labeled D-NG using flow cytometry as well as confocal microscopy. RAW macrophages were selected as model for phagocytosing cells of the reticuloendothelial system that usually rapidly engulf nanocarriers after systemic administration. However, only a minor uptake of D-NG was observed after dosing from 10 to 100 μg/mL. A weak increase in mean fluorescence intensity from 500 (basal autofluorescence) to 700 could be recorded by flow cytometry (Figure 5C1), and the resulting histogram provided only a minor but homogeneous shift of all cells to increased fluorescence (Figure 5C2). These observations were independent from the type of nanogel, as similar low mean fluorescence intensities and low TMR-positive cells were found for all species (D-NG, ND-NG, and NCL-NG; compare Supporting Information Figure S56). These results were further supported by confocal microscopy images of RAW macrophages incubated at 100 μg/mL for both 4 and 24 h (Figure 5C3 and Supporting Information Figure S55). All cells provided a homogeneous intracellular localization of some nanogels presumably inside vesicular compartments. Due to the fact that particle uptake was only observed upon high particle dosing at 100 μg/mL, these *in vitro* studies suggested the potentially useful property of these nanogel carriers to withstand rapid phagocytosis, making them applicable for systemic *in vivo* applications.

We therefore intravenously injected NIR-labeled D-NG into mice’s tail veins and observed after 24 h still a strong fluorescent signal distributed all over the body by the *in vivo* NIR imaging system (IVIS) (Figure 5D1). These findings confirmed that the nanogels remained in the blood circulation long enough to address multiple organs. Subsequently, major organs were harvested and imaged *ex vivo* to better quantitate the NIR signals (Figure 5D2). Semiquantitative analysis confirmed that nanogels were sequestrated not only in the liver, a typical nonspecific sink for systemically administered nanocarriers, and kidneys (probably due to the renal clearance properties of the soluble polymers after hydrolysis of ketal-cross-linked nanogels), but also in the spleen. The spleen is a central organ of the lymphatic system, being highly relevant for immunodrug delivery because it harbors many antigen-presenting cells (Figure 5D3). We, therefore, opted for a strategy to systemically deliver a TLR7/8 agonist into this organ and induce a local immune modulation.

### Effective and Safe TLR7/8 Agonist Delivery for Intravenous Routes of Administration

**Figure 6:**
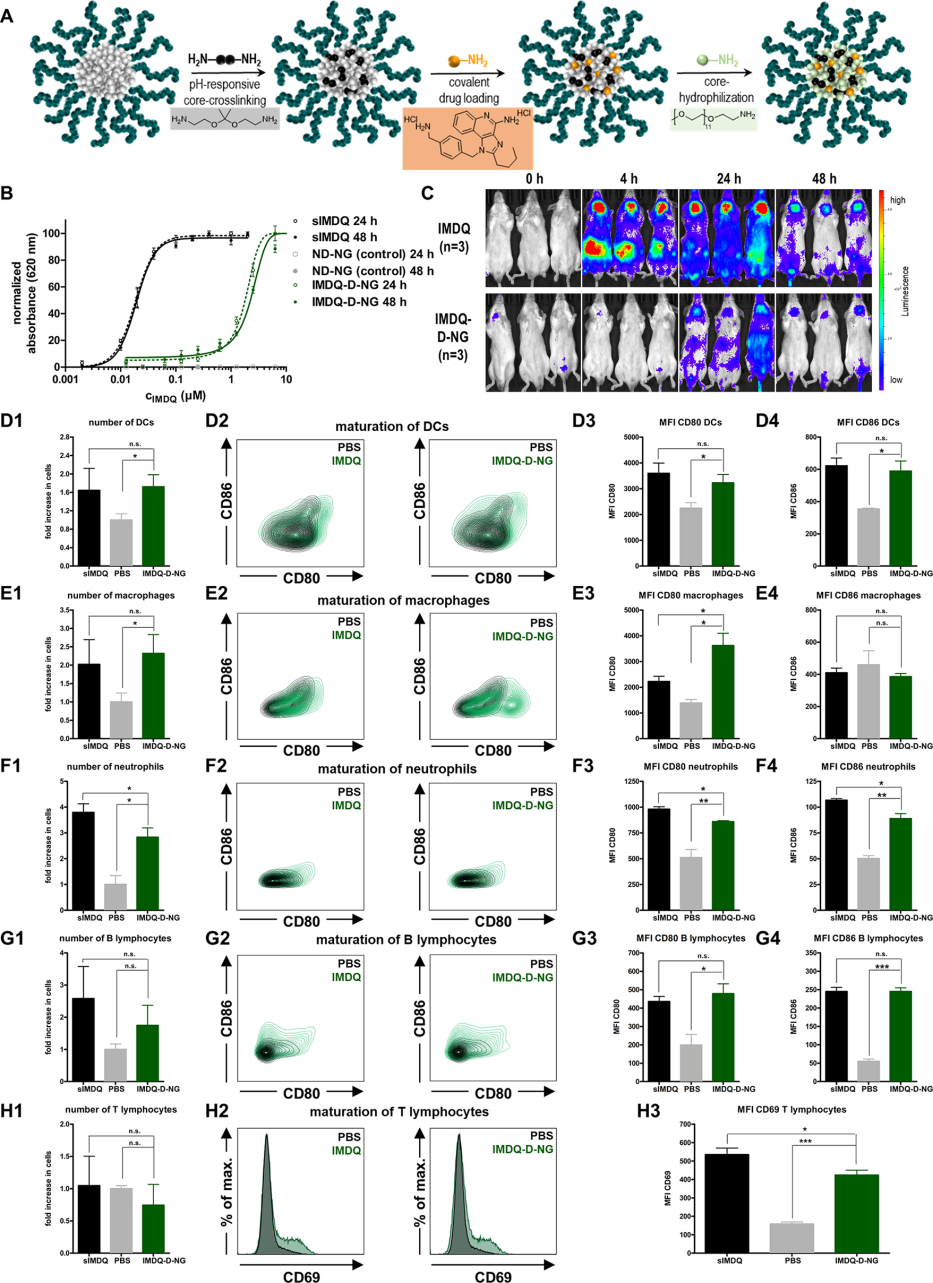
Squaric ester amide-based nanogels with covalently attached TLR7/8 agonist IMDQ. (A) Scheme of sequential nanogel fabrication with integral *in situ* conjugation of IMDQ. (B) TLR agonistic activity of soluble IMDQ (sIMDQ) and covalently attached IMDQ to degradable nanogels (IMDQ-D-NG) measured by NF-κB activation of RAW Blue cells after 24 and 48 h via a Quanti-Blue reporter assay (*n* = 4). (C) Bioluminescence images of heterozygous IFN-β (IFN-β^+/Δβ-luc^) reporter mice intravenously injected with sIMDQ and covalently attached IMDQ-D-NG (5 μg of IMDQ soluble or bound to nanogel in 100 μL of PBS); images were recorded before (0 h) as well as 4, 24, and 48 h after tail vein injection (*n* = 3). (D–H) Results of flow cytometric analysis of isolated splenocytes (compare Figure S60 for the applied gating procedure) after systemic administration of soluble IMDQ vs nanogel-conjugated IMDQ (1: fold increase of cells in the spleen in relation to PBS treated mice; 2: maturation plots by CD86 and CD80 or CD69 for T cells; 3: corresponding MFI values). (D) Dendritic cells (DCs), (E) macrophages, (F) neutrophils, (G) B lymphocytes, and (H) T lymphocytes.

The highly potent small-molecular TLR7/8 agonist IMDQ^18^ was selected as an immune modulatory compound to trigger maturation of splenic antigen-presenting cells. This compound has been demonstrated to activate TLR7/8 even when it is bound to macromolecular nanocarriers.^24,45,46^ Due to its primary benzylic amine, IMDQ can straightforwardly be conjugated to pendant squaric ester amide groups and, therefore, directly be incorporated into the nanogel fabrication process (Figure 6A). IMDQ conjugation did not affect the nanogel fabrication, as cross-linked nanogels (IMDQ-D-NG and IMDQ-ND-NG) were of similar size and the non-cross-linked species (IMDQ-NCL-NG) was again completely soluble (compare Supporting Information Figure S51). Quantification of the covalently attached IMDQ was conducted by ^1^H NMR spectroscopy. For the soluble NCL-NG species, a drug load of 2.7 wt % was found (compare Supporting Information Figure S52) being covalently attached to the polymer (confirmed by DOSY NMR, Figure S53).

For demonstrating TLR7/8 stimulation activity, we used a RAW-Blue macrophage reporter cell line that is genetically engineered to secrete embryonic alkaline phosphatase (SEAP) in response to TLR activation and downstream signaling via the NF-κB pathway. SEAP expression itself can readily be detected from the cell culture supernatant via UV/vis spectrophotometry after addition of a coloring substrate (Quanti-Blue assay). RAW Blue macrophages were pulsed with increasing doses of either soluble IMDQ (sIMDQ) or IMDQ-D-NG (empty nanogel D-NG served as a control). A strong TLR activation was found for sIMDQ already at sub-micromolar concentrations, while nonligated control nanogels did not induce any significant activation (Figure 6B). IMDQ-D-NG led to a dose-dependent activation, albeit less potent than sIMDQ, but still active in the micromolar concentration range. Similar activities were found after 24 as well as 48 h and could be confirmed for both IMDQ-ND-NG and IMDQ-NCL-NG (Figure S58), which may corroborate the weak cellular internalization. In parallel, RAW Blue cellular viability was monitored by an MTT assay. No significant influence on metabolic activity was found for all nanogel samples in the tested experimental window (Figure S57).

We then investigated the nanogel’s *in vivo* immune stimulatory activity with a special focus on the activation of antigen-presenting cells in the spleen while avoiding systemic off-target effects. The latter can be visualized by bioluminescence imaging of heterozygous BALB/c IFN-β (IFN-β^+/Δβ-luc^) reporter mice,^47^ in which luciferase as reporter gene is genetically linked to the expression of type I interferon IFN-β, a cytokine that is induced upon TLR7/8 stimulation. Mice were intravenously injected into the tail vein, and bioluminescence was monitored after 4, 24, and 48 h (Figure 6C and Supporting Information Figure S59). For sIMDQ, a strong signal was observed all over the body, reflecting a systemic inflammatory response in those mice. A prominent IFN-β expression occurred in the neck and abdomen and remained high up to 48 h postinjection. The nanogel-conjugated IMDQ, however, did not cause such a severe global immune activation all over the body. After 4 h no severe systemic inflammation was recorded. Only after 24 h a mild activity was observed with some expression in the abdomen and neck region, but with reduced gain compared to sIMDQ (Figure 6C and Supporting Information Figure S59). These observations clearly underline that severe systemic inflammatory responses are absent for the nanogel carrier system.

To further corroborate the absence of soluble IMDQ’s acute toxicity, we also monitored the systemic inflammatory cytokines in sera of mice treated with soluble IMDQ vs nanogel-conjugated IMDQ. The results are provided in the Supporting Information Figure S62–S64 and confirm the observations of the IFN-β reporter mice: After 4 h, increasing serum cytokine levels of TNF-α, IL-6, and INF-γ are found in mice treated with soluble IMDQ. They are significantly elevated compared to mice treated with PBS, while mice treated with nanogel-bound IMDQ only provide slightly increased but not significant TNF-α, IL-6, and INF-γ levels (Supporting Information Figure S62).

After 24 h, those cytokines are again decreased to levels of PBS-treated mice. The elevated cytokine levels for soluble IMDQ-treated mice caused further liver toxicities by increased ALT, AST, and GLDH enzyme serum activities (Supporting Information Figure S63). Additionally, further influence on pancreas toxicity by high lipase serum activity as well as kidney toxicity by elevated blood urea nitrogen (BUN) can be observed (Supporting Information Figure S64). These data underline again the improved safety profile for the IMDQ-loaded nanogels to circumvent these acute toxicities but focus the immune stimulatory potential of the TLR7/8 agonist to the spleen.

One may speculate that such reduced systemic off-target effects for the nanogel-conjugated IMDQ might simply be caused by the minor receptor activation potency or reduced bioavailability. However, when it comes to immune intervention strategies (e.g., vaccination or cancer immunotherapy), site-specific immune activation (i.e., spleen specific, rather than systemic) is of important relevance and the major goal of this study.

Previous biodistribution analysis confirmed that a sufficient amount of nanogels was found inside the spleen after systemic activation (Figure 5D). To further validate that IMDQ-loaded nanogels still activate spleen-resident immune cells, we isolated splenocytes from the mice 48 h after injection and performed quantitative flow cytometric analyses (for further details on the gating procedure compare Supporting Information Figure S60). Both sIMDQ and IMDQ-D-NG comparably increased the number of myeloid cells with no influence on B and T lymphocytes (Figure 6D–H1). Dendritic cells (DCs), macrophages, and neutrophils are known to express and respond to TLR7/8 activation effectively. Their numbers are therefore increased to a similar extent for both sIMDQ and IMDQ-D-NG (Figure 6D1–F1).

We then checked for maturation markers on those cells and found again that IMDQ-D-NG triggered their expression to a similar level as sIMDQ while omitting systemic inflammations (Figure 6D2–H2 and Supporting Information Figure S61). For DCs and neutrophils (Figure 6D3/4 and F3/4) the expression levels of CD80 and CD86 were significantly enhanced by IMDQ-D-NG as well as sIMDQ. They were also upregulated on B lymphocytes that in contrast to T lymphocytes have antigen presenting ability (Figure 6G3/4). Also, the T lymphocyte activation marker CD69 was induced, again comparably, by IMDQ-D-NG and sIMDQ treatment, indicating further downstream T lymphocyte activation (Figure 6H3).

Most interestingly, for macrophages no difference in the expression of CD86 was observed (Figure 6E4), as CD86 upregulation is more typical for DCs. However, CD80 expression was more significantly triggered by IMDQ-D-NG and even exceeded the expression levels of sIMDQ (Figure 6E3). Apparently, nanogel-bound IMDQ seems to have a preferential stimulation potential in those highly phagocytotic immune cells inside the spleen. These properties outperform the treatment with sIMDQ and make the nanogels highly interesting for further therapeutic immune interventions.

Altogether, these observations confirm the hypothesis that our nanocarrier can deliver the TLR7/8 agonist into the spleen and maintain its immune stimulatory activity inside that organ. It prevents massive systemic immune activations and subsequent severe inflammatory off-target effects but conserves organ-specific immune stimulation inside the spleen, especially to resident macrophages. Since this organ is highly relevant for further immune intervention strategies including cancer immunotherapy or vaccination,^48,49^ we consider squaric ester-based, pH-degradable nanogels as a relevant platform to exploit further systemic immunologic adjuvant strategies.

## Conclusions

In this study, we have introduced polymerizable squaric ester amides as novel functionable tool to robustly fabricate pH-responsive nanogels serving as a versatile nanocarrier platform for safe delivery of the highly potent immunomodulator IMDQ after intravenous administration. To the best of our knowledge, the synthesis of methacrylamide monomers with a functional pendant squaric ester amide moiety has not been reported before. Its subsequent controlled RAFT block copolymerization as well as its self-assembling behavior in polar protic solvents provides access to precursor micelles with amine-reactive cores. Through their sequential amidation, covalent dye or drug loading, acid-sensitive cross-linking, and core-transformation from hydrophobic to hydrophilic polarity can be performed, affording fully hydrophilic nanogels with profound stability in plasma and the bloodstream, with modest uptake by phagocytic cells and with an inherently triggered degradation upon exposure to mildly endolysosomal pH conditions. The carriers’ potential to covalently conjugate IMDQ as a highly potent immunomodulator enables the drug’s safe and efficient delivery by preventing systemic off-target inflammation but retaining its spatial activity in the spleen. These findings underline the potential of this carrier platform to access intravenous administrations routes for small-molecular TLR7/8 agonists (and other relevant immune modulators) and, thus, provide novel opportunities to explore their superior adjuvant potency for systemic vaccination or cancer immunotherapy purposes.

## Supporting Information Available

Experimental details including materials, instrumentation, monomer and polymer syntheses, and characterization, nanogel fabrication and characterization, IMDQ ligation and quantification, *in vitro* and *in vivo* evaluation (PDF)
