# Supplementary material for: Squaric Ester-Based, pH-Degradable Nanogels: Modular Nanocarriers for Safe, Systemic Administration of Toll-like Receptor 7/8 Agonistic Immune Modulators
Source: J Am Chem Soc. 2021 Jun 24;143(26):9872–83. doi: 10.1021/jacs.1c03772 (PMC8267846; doi:10.1021/jacs.1c03772)
Supplement: Supplementary file 1 — ja1c03772_si_001.pdf [file ja1c03772_si_001.pdf]

Supporting Information

Squaric Ester-Based, pH-Degradable Nanogels: Modular Nanocarriers for Safe, Systemic Administration of Toll-Like Receptor 7/8 Agonistic Immune Modulators

Anne Huppertsberg[‖], Leonard Kaps[‡,Π], Zifu Zhong[†], Sascha Schmitt[‖], Judith Stickdorn[‖], Kim Deswarte[Δ], Francis Combes[§], Christian Czysch[‖], Jana De Vrieze[†], Sabah Kasmi[†], Niklas Choteschovsky[‡], Adrian Klefenz[‡], Carolina Medina-Montano[º], Pia Winterwerber[‖], Chaojian Chen[‖], Matthias Bros[º], Stefan Lienenklaus[$], Niek N. Sanders[§], Kaloian Koynov[‖], Detlef Schuppan[‡,∇], Bart N. Lambrecht[Δ,⊥], Sunil A. David[#], Bruno G. De Geest[†], and Lutz Nuhn[‖,*]

‖ Max Planck Institute for Polymer Research, 55128 Mainz, Germany;
‡ Institute for Translational Immunology and Research Center for Immune Therapy, University Medical Center, Johannes Gutenberg-University Mainz, 55131 Mainz, Germany;
Π Department of Internal Medicine I, University Medical Centre of the Johannes Gutenberg-University Mainz, 55131 Mainz, Germany;
† Department of Pharmaceutics and Cancer Research Institute Ghent (CRIG), Ghent University, Ghent 9000, Belgium;
Δ Department of Internal Medicine and Pediatrics, Ghent University, VIB Center for Inflammation Research, Ghent 9000, Belgium;
§ Laboratory of Gene Therapy, Department of Nutrition, Genetics and Ethology, Ghent University, Merelbeke 9820, Belgium;
º Department of Dermatology, University Medical Center of Johannes Gutenberg-University Mainz, 55131 Mainz, Germany;
$ Institute for Laboratory Animal Science and Institute of Immunology, Hannover Medical School, 30625 Hannover, Germany;
∇ Division of Gastroenterology, Beth Israel Deaconess Medical Center, Harvard Medical School, Boston, MA 02215, United States;
⊥ Department of Pulmonary Medicine, Erasmus University Medical Center, Rotterdam 3015, Netherlands;
ViroVax, LLC, Lawrence, Kansas 66047, United States.

Table of Content

1. Chemicals and Solvents **3**

2. Instrumentation **4**

2.1 Nuclear Magnetic Resonance (NMR) Spectroscopy 4

2.2 Electrospray-Ionization mass spectrometry (ESI-MS) 4

2.3 Ultraviolet-Visible Spectroscopy (UV-Vis) 4

2.4 Size-Exclusion Chromatography (SEC) 4

2.5 Dynamic Light Scattering (DLS) 4

2.6 Fluorescence Correlation Spectroscopy (FCS) 5

2.7 Atomic Force Microscopy (AFM) 5

2.8 Transmission Electron Microscopy (TEM) 6

3. Syntheses **7**

3.1 Monomers 7

3.2 Synthesis of macro-trithiocarbonate-chain transfer agent (macro-TTC-CTA) 23

3.3 Block copolymerization of MA-SQ with PEG-TTC-CTA 26

3.4 Homopolymerization of MA-SQ with TTC-CTA 29

3.5 Reaction kinetics of RAFT homopolymerization 31

3.6 Polymer-analogous reaction of homopolymer by amidation with primary, secondary, or tertiary amines 32

3.7 Block copolymer-analogous reaction of self-assembled micelles by amidation with primary amines 36

4. Nanogel Fabrication **37**

4.1 Preparation of nanogels 37

4.2 IMDQ quantification via ^{1}H NMR analysis 41

4.3 Degradation/hydrolysis study of ketal-crosslinked nanogels 42

5. *In Vitro* Experiments **43**

5.1 Cell culturing 43

5.2 Cell uptake experiment in RAW Blue-macrophages using confocal fluorescence microscopy 43

5.3 Flow cytometry (FACS) 44

5.4 Cellular metabolic activity of RAW-Blue macrophages using MTT assay 44

5.5 RAW-Blue macrophage TLR reporter assay 45

6. *In Vivo* Experiments **46**

6.1 Biodistribution imaging of 800RS-labeled nanogels 46

6.2 Bioactivity imaging of IMDQ-loaded nanogels 46

6.3 Flow cytometry analysis of splenic innate immune activation 47

6.4 Acute toxicity studies 50

7. Statistical analysis **51**

1. Chemicals and Solvents

Unless otherwise stated, all chemicals were obtained from commercial sources, such as Sigma Aldrich (Taufkirchen, Germany), TCI Chemicals (Tokyo, Japan) or Rapp Polymere (Tübingen, Germany) and used as received. Fluorescent dyes Oregon Green 488 cadaverine and tetramethylrhodamine cadaverine were obtained from Thermo Fisher Scientific (Waltham, MA, USA) and 800RS-amine was purchased from Li-Cor Biosciences (Lincoln, NE, USA).

Azobisisobutyronitrile (AIBN) was recrystallized from ethanol twice, prior to use. TLR 7/8 agonist 1-(4-(aminomethyl)benzyl)-2-butyl-1-H-imidazo[4,5-c]quinolin-4-amine (IMDQ) could be provided following an earlier report.[1]

Solvents (HPLC grade) were purchased from Acros Organics (Geel, Belgium) and Thermo Fisher Scientific. Deuterated solvents were obtained from Sigma Aldrich. Millipore water was prepared using a MILLI-Q® Reference A+ System. Silica gel chromatography was performed using silica obtained from Machery-Nagel (particle size: 0.063–0.2 mm). For dialysis Spectra/Por7™ dialysis membranes with a molecular weight cut-off of 1000 g/mol were used.

Cell culture medium and supplements as well as Dulbecco's PBS were obtained from Thermo Fisher Scientific. The RAW-Blue reporter cell line (264.7) and QUANTI-Blue™ substrate was purchased from InvivoGen (San Diego, CA, USA). RAW-Blue macrophages were cultured in DMEM-GlutaMAX™ medium, which was supplemented with 10% fetal bovine serum, 1% penicillin/streptomycin, 0.02 % normocin, and 0.01% zeocin at 37°C with 5% CO_2 saturation.

[1] Shukla, N. M.; Malladi, S. S.; Mutz, C. A.; Balakrishna, R.; David, S. A. Structure-Activity Relationships in Human Toll-like Receptor 7-Active Imidazoquinoline Analogues. *J. Med. Chem.* **2010**, 53 (11), 4450–4465.

2. Instrumentation

2.1 Nuclear Magnetic Resonance (NMR) Spectroscopy

^{1}H, and ^{13}C NMR spectra were recorded at room temperature (RT) on a Bruker Avance III 300 MHz, Bruker Avance III 400 MHz or Bruker Avance III 500 MHz spectrometer. The chemical shifts (δ) are given in parts per million (ppm) relative to TMS. NMR spectra were processed with the software MestReNova 11.0.4 by Mestrelab Research. Samples were prepared in deuterated solvents and their corresponding signals referenced to residual non-deuterated solvent signals.

2.2 Electrospray-Ionization mass spectrometry (ESI-MS)

ESI mass spectra were recorded by an Agilent 6545 QTOF-MS (Santa Clara, CA, USA). Samples were prepared at 0.1 mg/mL in methanol.

2.3 Ultraviolet-Visible Spectroscopy (UV-Vis)

UV/Vis spectra of the polymer-analogous reaction of homopolymer with amines were recorded on a V-630 spectrophotometer equipped with a Peltier thermostatted ETC-717 single cell holder purchased from JASCO (Pfungstadt, Germany). Measurement conditions of 20 °C were guaranteed by a V50 water thermostat from Krüss Optronic (Hamburg, Germany).

RAW-Blue and MTT assay analysis were performed using a Spark 20M Multimode Microplate Reader from Tecan Trading AG (Mannedorf, Switzerland).

2.4 Size-Exclusion Chromatography (SEC)

SEC was performed using 20A system equipped with a 20A ISO-pump and a 20A refractive index (RI) detector purchased from Shimadzu (Tokyo, Japan). N,N-dimethylacetamide (DMAc) containing 50 mM lithium bromide was used as eluent at a flow rate of 0.7 mL/min and a column temperature of 50 °C. PLgel 5 µm MIXED-D columns were obtained from Agilent Technologies (Santa Clara, CA, USA) and calibrated using poly(methyl methacrylate) (PMMA) standards obtained from PSS (Mainz, Germany).

As internal standard toluene was added to the samples. Samples were prepared at 1 mg/mL and filtered through GHP syringe filters (0.2 µm pore size, Acrodisc) prior to injection. The data was processed with the software PSS WINGPC UniChrom.

2.5 Dynamic Light Scattering (DLS)

Single-angle DLS measurements were performed at 25 °C using a Malvern ZetaSizer Nano S purchased from Malvern Instruments Ltd. (Malvern, Great Britain) with a He/Ne Laser (λ = 633 nm) at a fixed scattering angle of 173°. All measurements were performed in triplicate. The obtained data was processed by cumulant fitting for D_z and PDI, or by CONTIN fitting for intensity-, volume-, and number-weighted particle size distribution. Samples were prepared at 0.1 mg/mL. Dust was removed prior to each measurement by filtration through GHP syringe filters (0.2 µm pore size, Acrodisc).

2.6 Fluorescence Correlation Spectroscopy (FCS)

Prior to FCS measurements fluorescently Oregon Green-labeled nanogels were purified from remaining unbound dye by spin-filtration. Thus, 2 mg of the respective nanogel type was re-dispersed in 1 mL PBS using sonification, before unbound Oregon Green cadaverine dye was removed by spin-filtration of the nanogel dispersion using centrifugal filter units (regenerated cellulose, MWCO: 10,000 g/mol). After centrifugation 0.8 mL PBS was added and centrifuged again. This procedure was repeated until complete disappearance of Oregon Green-derived absorbance in the filtrate was monitored by UV-Vis measurements.

To monitor degradation of pH-responsive nanogels under acidic conditions 10 µL of nanogel dispersion (2 mg/mL in PBS) was diluted with 90 µL Ac/HAc buffer (pH 5.2). FCS measurements were performed after 0, 90, 120, 180, 270, and 480 min.

To investigate the nanogels plasma stability 10 µL of nanogel dispersion (2 mg/mL in PBS) was incubated with 90 µL of human plasma at 37 °C under vigorous shaking. FCS data were recorded after 0, 6, and 24 h.

FCS measurements were performed using a commercial LSM880 setup (Carl Zeiss, Jena, Germany) equipped with a C-Apochromat 40x/1.2 W water immersion objective. For excitation of Oregon Green an argon laser (488 nm) was used. The fluorescence light was collected with the same objective and after passing a pinhole directed to a spectral detection unit (Quasar, Carl Zeiss). There the fluorescence light is separated spectrally by a grating element on a 32 channel array of GaAsP detectors operating in a single photon counting mode. The emission for Oregon Green was collected in the spectral range of 508-606 nm.

All measurements were performed in eight-well polystyrene-chambered coverglass (Laboratory-Tek, Nalge Nunc International, Penfield, NY, USA). The obtained FCS autocorrelation curves were fitted with the theoretical model function for an ensemble of either one or two types of freely diffusing fluorescence species. The fits yielded the diffusion coefficients (D) of the studied species. Finally, the hydrodynamic radii (R_H) were calculated using the Stokes-Einstein relation: $R_{\mathrm{H}} = \frac{k_{\mathrm{B}}T}{6\pi\eta D}$, where k_B is the Boltzmann constant, T is the temperature, and η is the viscosity of the solvent.

2.7 Atomic Force Microscopy (AFM)

AFM measurements were performed with a Bruker Dimension FastScan Bio® atomic force microscope, which was operated in PeakForce mode. AFM probes with a nominal spring constant of 0.25 N/m (FastScan-D, Bruker) were applied. For sample preparation 30 µL of nanogel dispersion (0.5 mg/mL in Millipore water) was dropcasted onto a freshly cleaved mica substrate (circular, 15 mm). Samples were scanned with scan rates of 0.994 Hz and data was processed using NanoScope Analysis 1.8.

2.8 Transmission Electron Microscopy (TEM)

For preparation of TEM images 5 μL of nanogel dispersion (0.5 mg/mL in Millipore water containing 0.1 wt% ammonia) was added onto a carbon-coated copper grid. After air-drying TEM measurements were performed using a JEOL JEM-1400 TEM operating at an accelerating voltage of 120 kV.

3. Syntheses

3.1 Monomers

3.1.1 N-(3-(2-ethoxy-3,4-dioxocyclobut-1-en-1-yl)amino)propyl)methacrylamide, Squaric ester amide methacrylamide (MA-SQ)

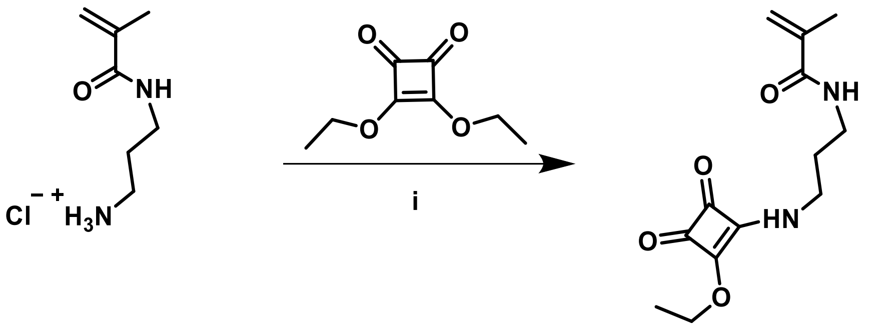

Figure S 1: Synthesis of MA-SQ, i) TEA, EtOH/H_2O, RT, 16 h, yield: 90 %.

In a round bottom flask, squaric acid diethyl ester (7.86 g, 46 mmol, 1.1 eq.) and triethylamine (TEA) (6.37 g, 63 mmol, 1.5 eq.) were diluted in ethanol (70 mL). To this solution N-(3-aminopropyl)methacrylamide hydrochloride (APMA) dissolved in mixture of water (30 mL) and ethanol (30 mL) was slowly added *via* dropping funnel over 2 h. The reaction mixture was allowed to stir for 16 h at RT. After a negative Ninhydrin test indicated complete conversion of the primary amine APMA, toluene (2 x 250 mL) was added to the reaction mixture to remove ethanol and water by azeotropic evaporation *in vacuo*. To remove MEHQ inhibitor the resulting crude product was dissolved in a mixture of ethyl acetate (15 mL) and ethanol (30 mL) and purified by passing over a short column of activated basic alumina. Again, toluene (2 x 50 mL) was added to remove the solvents by azeotropic evaporation *in vacuo*. Further purification of the product was achieved by silica gel chromatography using ethyl acetate as eluent. The product was obtained as white solid (10.10 g, 90 %).

^{1}H-NMR (300 MHz, DMSO-d_6): δ (ppm) = 8.73 (s, 0.5H, **a**)[a)] 8.54(s, 0.5H, **a**)[a)], 7.90 (s, 1H, **b**), 5.63 (s, 1H, **c**), 5.32 (s, 1H, **d**), 4.64 (m, 2H, **e**), 3.48[a)] (m, 1H, **f**), 3.29[a)] (m,1H, **f**) 3.13 (m, 2H, **g**) 1.84 (s, 3H, **h**)1.69 (m, 2H, **i**), 1.35 (m, 3H, **j**). [a)] due to rotamers a splitting of the signals is observed.

^{13}C-NMR (75 MHz, DMSO-d_6): δ (ppm) = 189.39 (**a**)[a)], 189.30 (**a**)[a)], 182.12(**b**)[a)], 181.92 (**b**)[a)], 177.00 (**c**)[a)], 176.54 (**c**)[a)], 172.58 (**d**)[a)], 172.22 (**d**)[a)], 167.55 (**e**)[a)], 167.52(**e**)[a)], 139.94 (**f**), 118.95 (**g**), 68.80 (**h**), 41.67 (**i**), 41.33 (**i**), 36.00, (**j**) 30.24 (**k**), 29.76 (**l**), 18.63 (**m**), 15.65 (**n**). [a)] due to rotamers a splitting of the signals is observed.

ESI-MS: [m/z] = 267.17 [M+H]$^{+}$ calc. 267.13), 289.19 [M+Na]$^{+}$ (calc. 289.12), 555.57 [2M+Na]$^{+}$ (calc. 555.24).

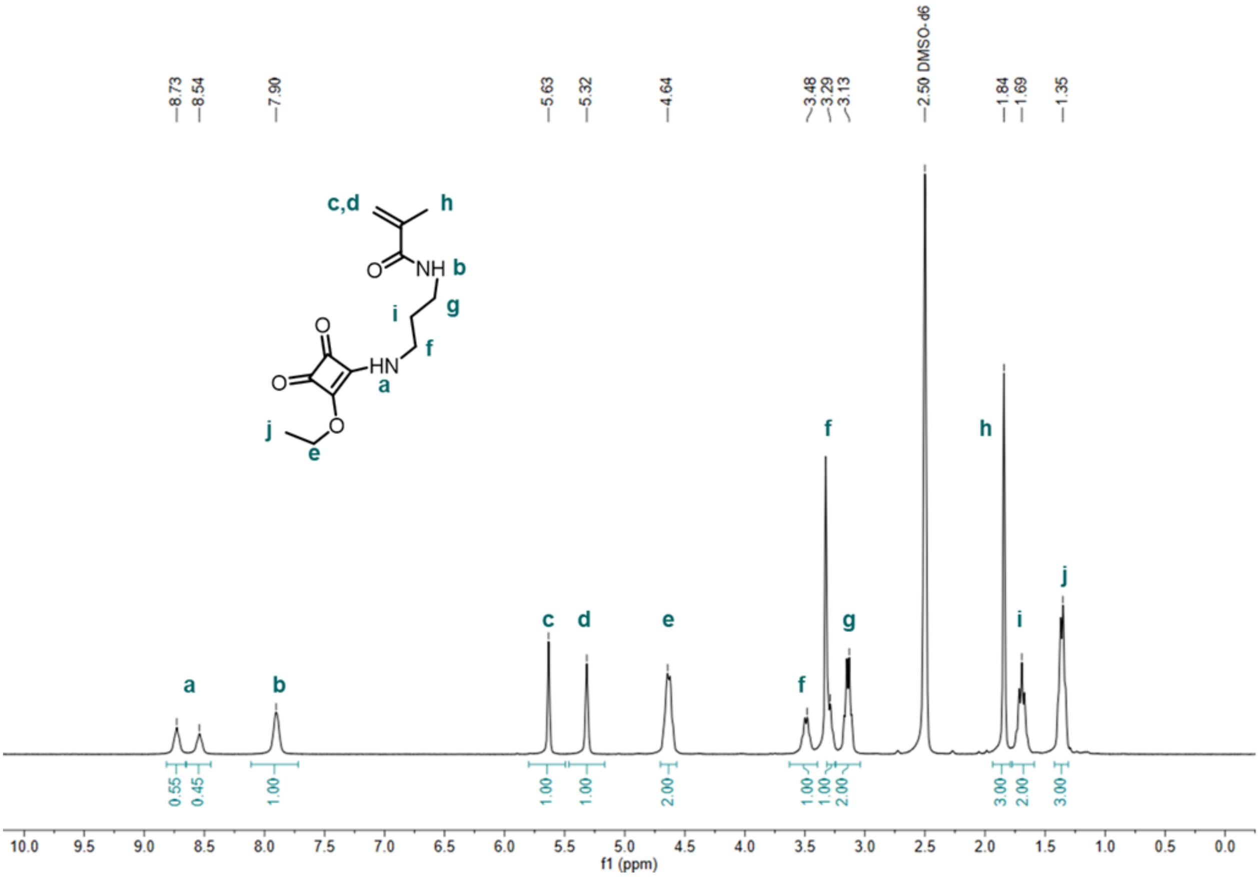

Figure S 2: ^{1}H-NMR spectrum (300 MHz) of MA-SQ in DMSO-d_6.

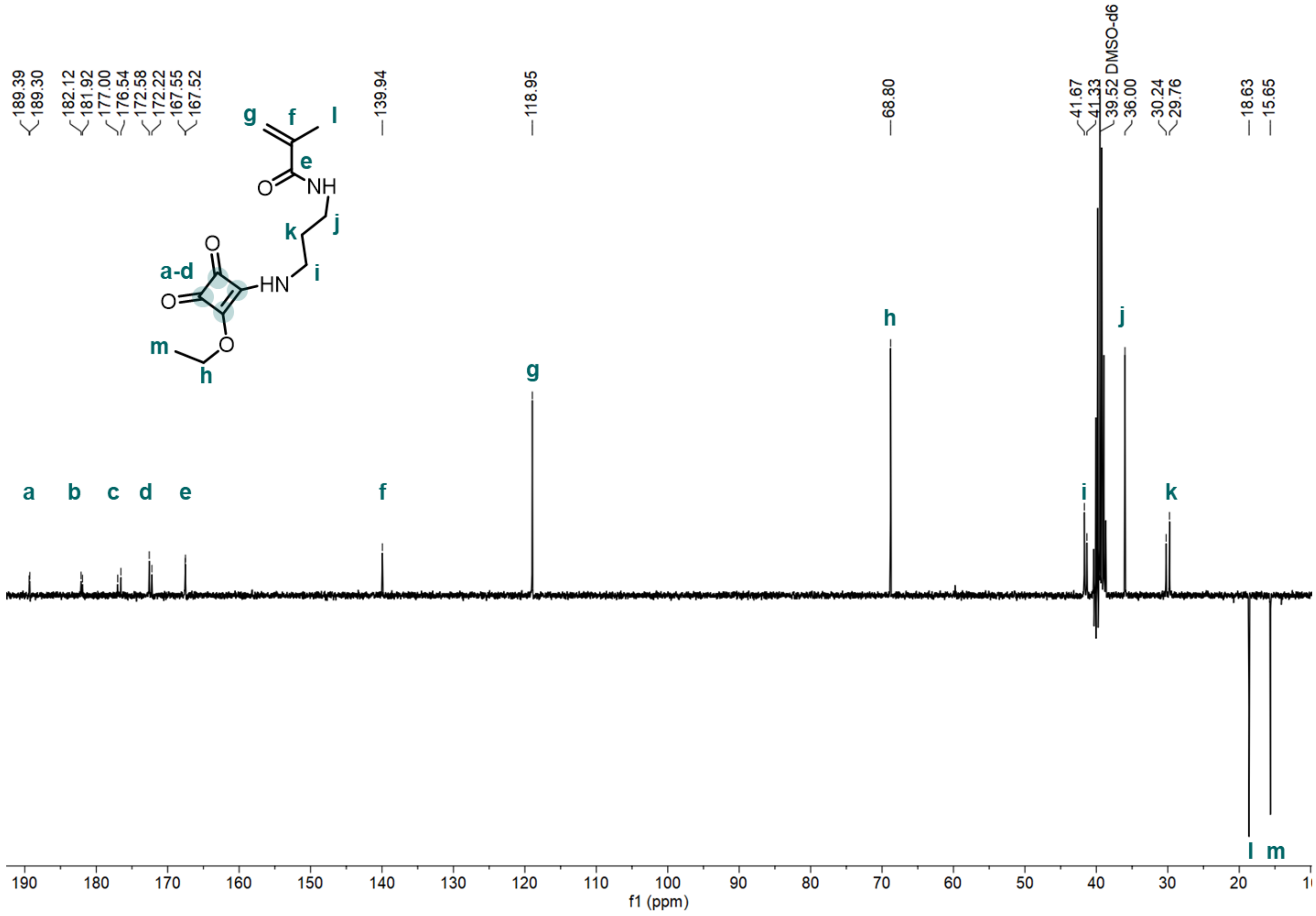

Figure S 3: APT ^{13}C NMR spectrum (75 MHz) of MA-SQ in DMSO-d_6.

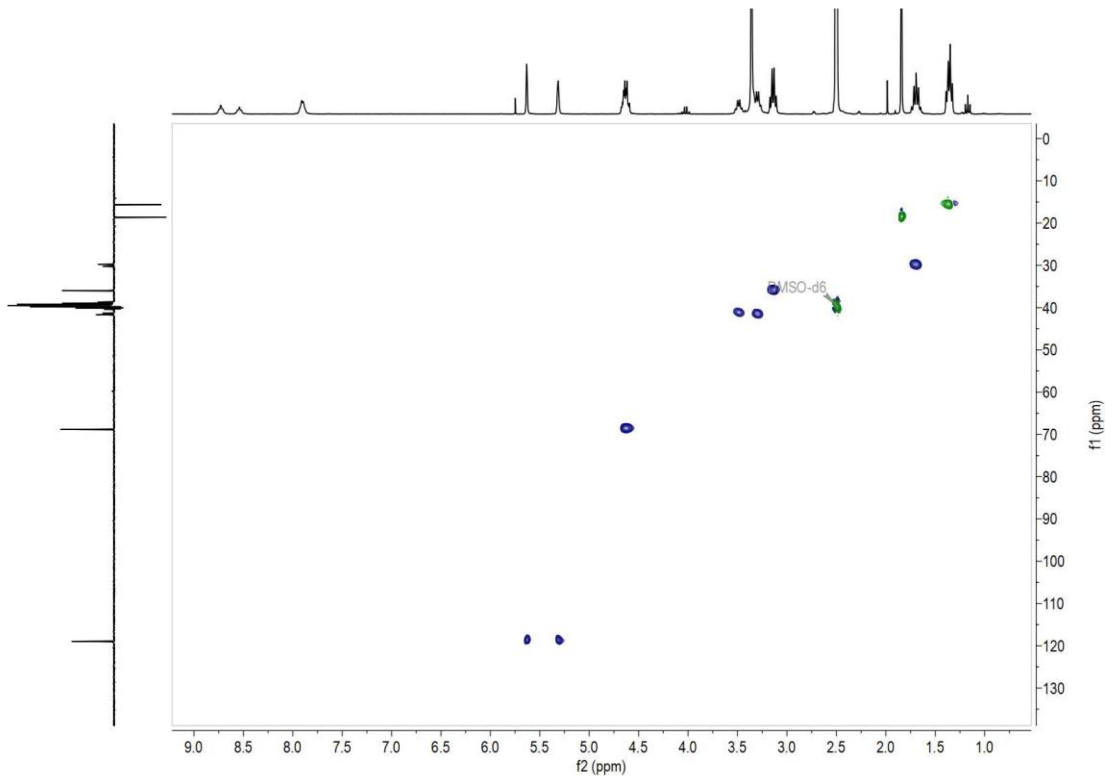

Figure S 4: 2D HSQC NMR spectrum of MA-SQ in DMSO-d$_6$.

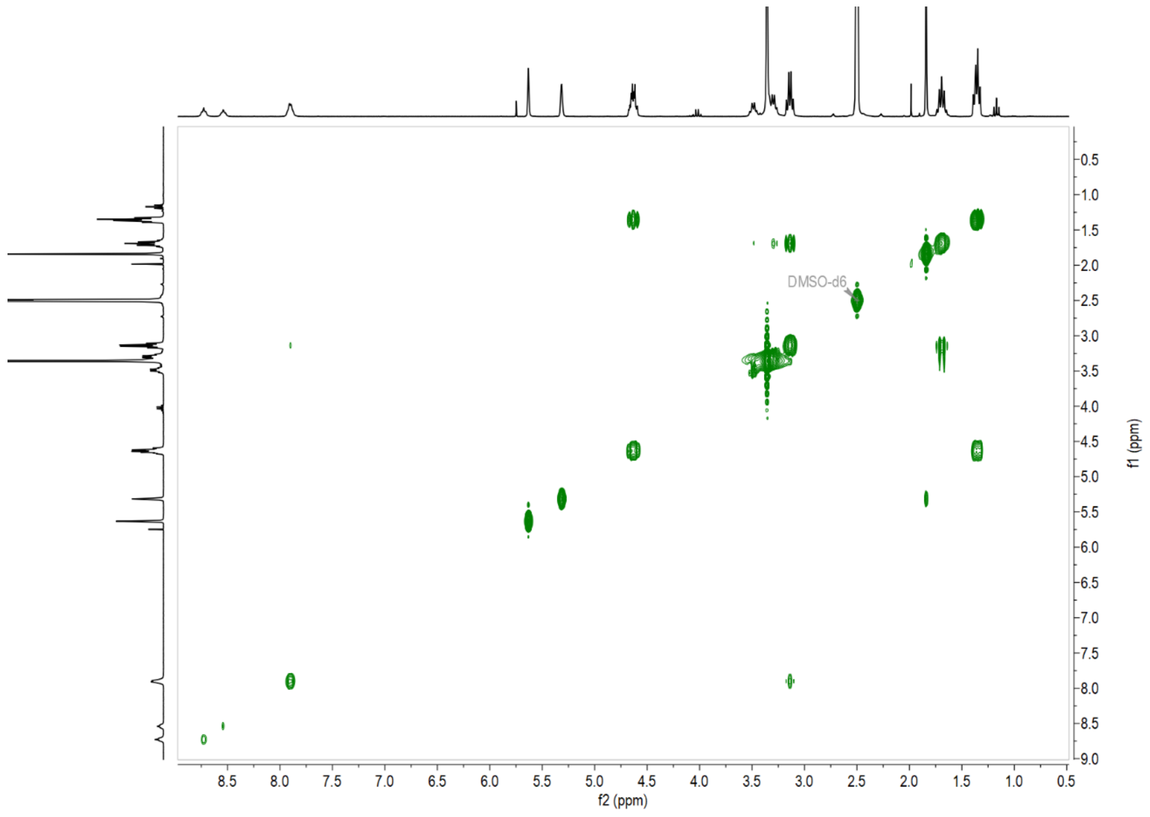

Figure S 5: 2D COSY NMR spectrum of MA-SQ in DMSO-d$_6$.

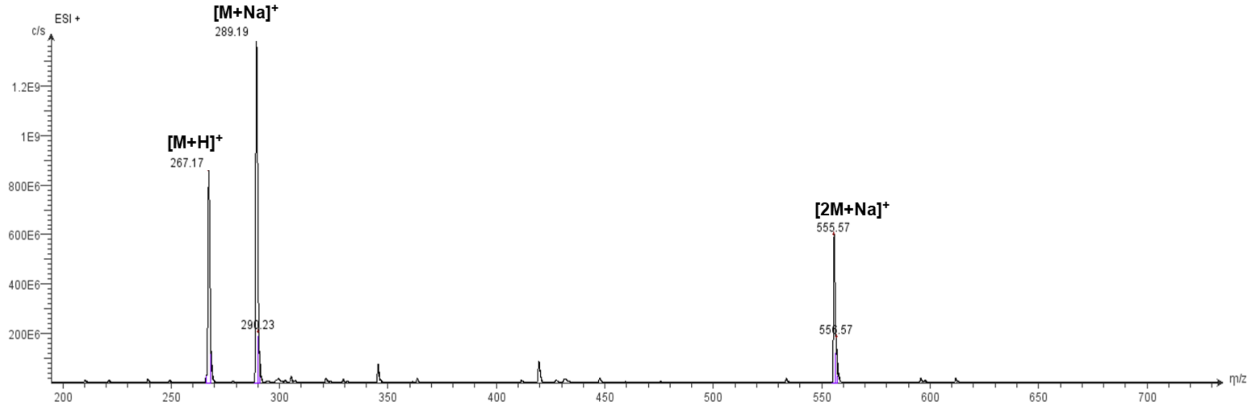

Figure S 6: ESI-MS spectrum of MA-SQ (positive ion mode).

3.1.2 N-(3-((2-ethoxy-3,4-dioxocyclobut-1-en-1-yl)amino)propyl)acrylamide, Squaric ester amide acrylamide (A-SQ)

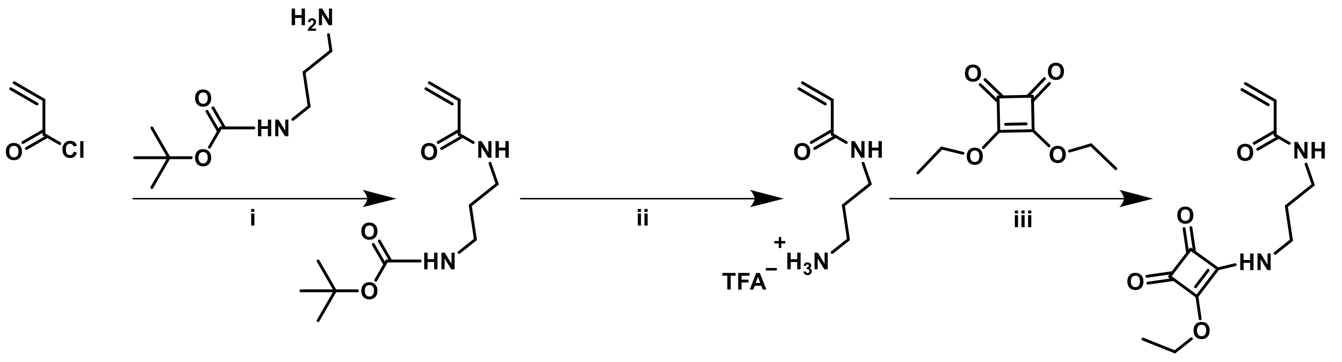

Figure S 7: Synthesis of A-SQ: i) TEA, DCM, 0 °C, 16 h, yield: 32 %, ii) TFA, DCM, RT, 4 h, yield: quantitative, and iii) TEA, EtOH, RT, yield: 64 %.

Synthesis of N-(Boc-3-aminopropyl)acrylamide (i)

In an oven-dried round-bottom flask, acryloyl chloride (6.89 mmol, 0.56 mL, 1.2 eq.) was dissolved under Argon atmosphere in anhydrous DCM (100 mL). The solution was cooled for 30 min using an ice bath, before a mixture of N-Boc-1,3-propanediamine (5.74 mmol, 1.00 g, 1 eq.) and TEA (37.31 mmol, 5.3 mL, 6.5 eq.) dissolved in anhydrous DCM (100 mL) was added dropwise *via* dropping funnel over 3 h. The reaction mixture was allowed to stir overnight on ice melting to water, before being extracted with brine (3x 200 mL). The combined organic layers were dried over Na_2SO_4, filtered, and concentrated *in vacuo*. Further purification of the product was achieved by silica gel chromatography using a mixed eluent of ethyl acetate and n-hexane (2:1). The product was obtained as yellow oil (0.41 g, 32%).

^{1}H-NMR (300 MHz, DMSO-d_6): δ (ppm) = 8.05 (t, *J* = 5.4 Hz, 1H, **a**), 6.78 (t, *J* = 5.7 Hz, 2H, **b**), 6.19 (dd, *J* = 17.1, 9.9 Hz, 1H, **c**), 6.05 (dd, *J* = 17.1, 2.5 Hz, 1H, **d**), 5.56 (dd, *J* = 9.9, 2.5 Hz, 1H, **e**), 3.11 (q, *J* = 6.9 Hz, 2H, **f**), 2.92 (q, *J* = 6.8 Hz, 2H, **g**), 1.53 (p, *J* = 7.1 Hz, 2H, **h**), 1.37 (s, 9H, **i**).

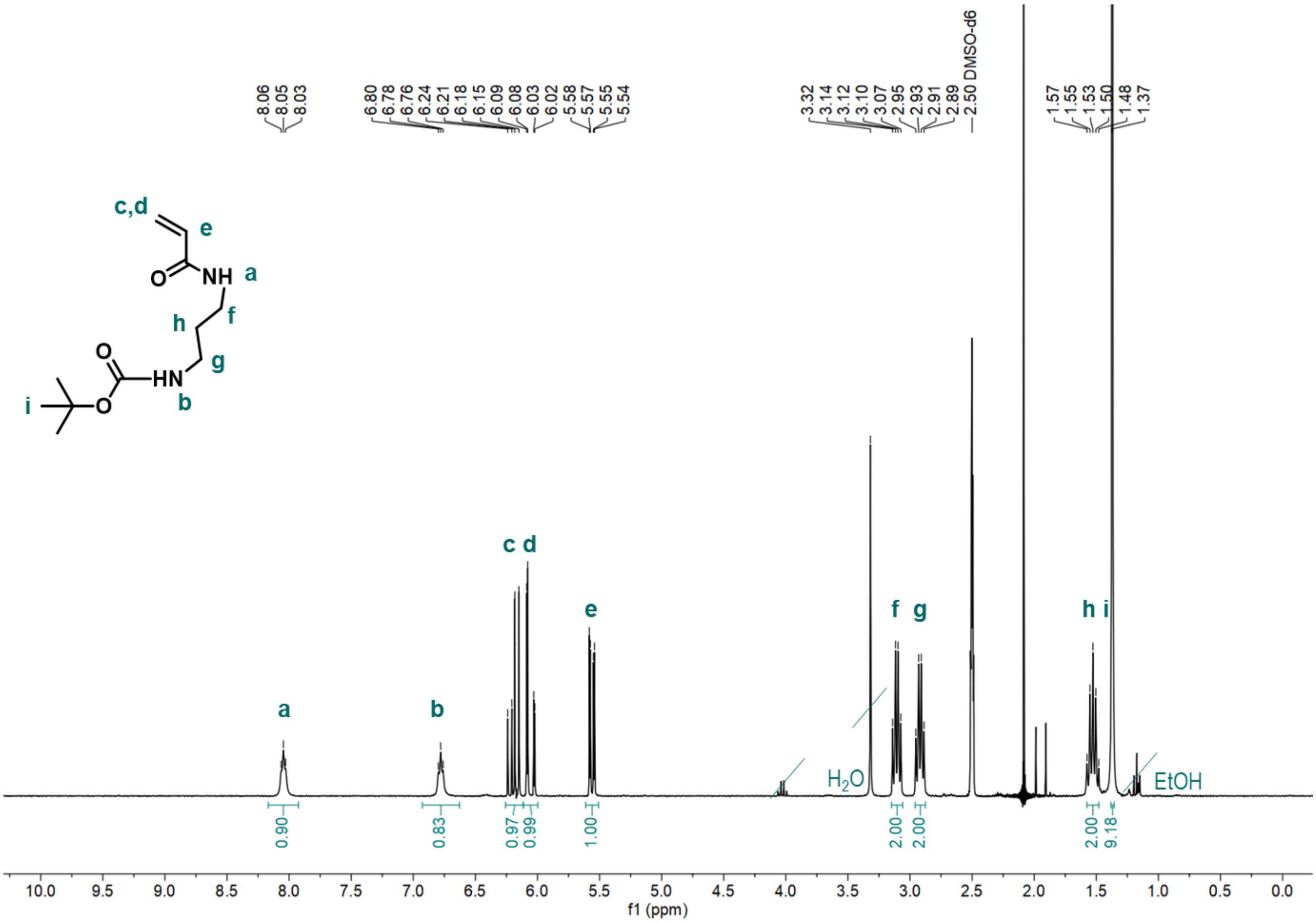

Figure S 8: ^{1}H-NMR spectrum (300 MHz) of N-(Boc-3-aminopropyl)acrylamide in DMSO-d_6.

Synthesis of N-(3-aminopropyl)acrylamide TFA salt (ii)

In an oven-dried round bottom flask, N-(Boc-3-aminopropyl)acrylamide (400 mg, 1.75 mmol, 1 eq.) was dissolved under Argon atmosphere in anhydrous DCM (10 mL). This solution was treated with a mixture of TFA and anhydrous DCM (10 mL, v:v, 1:1). The reaction mixture was allowed to stir for 4 h at RT, before toluene (2x 100 mL) was added to remove TFA by azeotropic evaporation *in vacuo*. The remaining oily crude product was dissolved in ethanol (25 mL), filtered, and concentrated *in vacuo* affording the product as amber oil (425 mg, quantitative).

^{1}H-NMR (400 MHz, DMSO-d_6): δ (ppm) = 8.28 (t, *J* = 5.9 Hz, 1H, **a**), 7.81 (s, 3H, **b**), 6.21 (dd, *J* = 17.1, 10.0 Hz, 1H, **c**), 6.08 (dd, *J* = 17.1, 2.3 Hz, 1H, **d**), 5.60 (dd, *J* = 10.0, 2.3 Hz, 1H, **e**), 3.19 (q, *J* = 6.7 Hz, 2H, **f**), 2.86 – 2.71 (m, 2H, **g**), 1.71 (p, *J* = 14.3, 6.8 Hz, 2H, **h**).

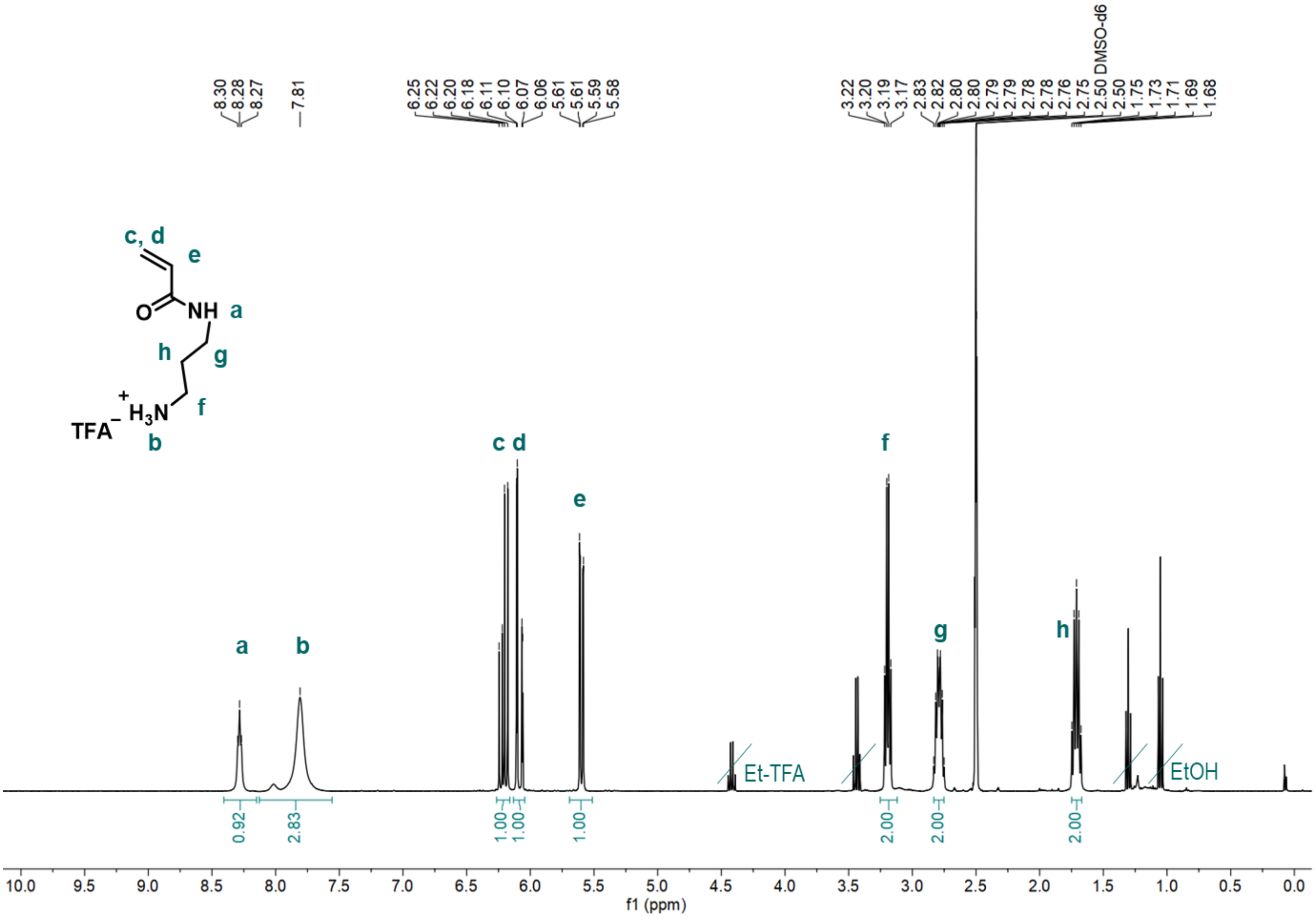

Figure S 9: ^{1}H-NMR spectrum (400 MHz) of N-(3-aminopropyl)acrylamide TFA salt in DMSO-d_6.

Synthesis of N-(3-((2-ethoxy-3,4-dioxocyclobut-1-en-1-yl)amino)propyl)acrylamide, Squaric ester amide acrylamide (A-SQ)

In a round bottom flask, squaric acid diethyl ester (326.6 mg, 1.92 mmol, 1.1 eq.) and TEA (264.8 mg, 2.62 mmol, 1.5 eq.) were diluted in ethanol (20 mL). To this solution N-(3-aminopropyl)acrylamide TFA salt (423.0 mg, 1.75 mmol, 1 eq.) dissolved in ethanol (5 mL) was slowly added *via* dropping funnel over 1 h, whereby the product formed a white precipitate. After a reaction time of 6 h, the reaction mixture was concentrated *in vacuo*. Further purification of the crude product was achieved by silica gel chromatography using ethyl acetate as eluent. The product was obtained as white crystalline powder (282.1 mg, 64 %).

^{1}H-NMR (400 MHz, DMSO-d_6): δ (ppm) = 8.74 (t, 6.0 Hz, 0.5H, **a**) [a)], 8.56 (t, 6.0 Hz, 0.5H, **a**) [a)], 8.09 (t, J = 5.6 Hz, 1H, **b**), 6.19 (dd, J = 17.0, 10.0 Hz, 1H, **c**), 6.06 (dd, J = 17.1, 2.2 Hz, 2H, **d**), 5.57 (dd, J = 10.0, 2.3 Hz, 1H, **e**), 4.64 (qd, J = 6.9 Hz, 2H, **f**), 3.49 (q, J = 6.1 Hz, 1H, **g**) [a)], 3.30 (q, J = 6.1 Hz, 1H, **g**) [a)], 3.16 (q, J = 6.9, 5.7 Hz, 2H, **h**), 1.69 (p, J = 7.0 Hz, 2H, **i**), 1.36 (td, J = 7.3 Hz, 3H, **j**). [a)] due to rotamers a splitting of the signals is observed.

^{13}C-NMR (101 MHz, DMSO-d_6): δ (ppm) = 189.28 (**a**)[a)], 182.03 (**b**)[a], 176.77 (**c**)[a)], 172.38 (**d**)[a)], 164.62 (**e**), 131.71 (**f**), 124.97 (**g**), 68.76 (**h**), 41.49 (**i**) [a)], 35.75 (**j**) [a)], 30.02 (**k**), 15.62 (**l**). [a)] due to rotamers a splitting of the signals is observed.

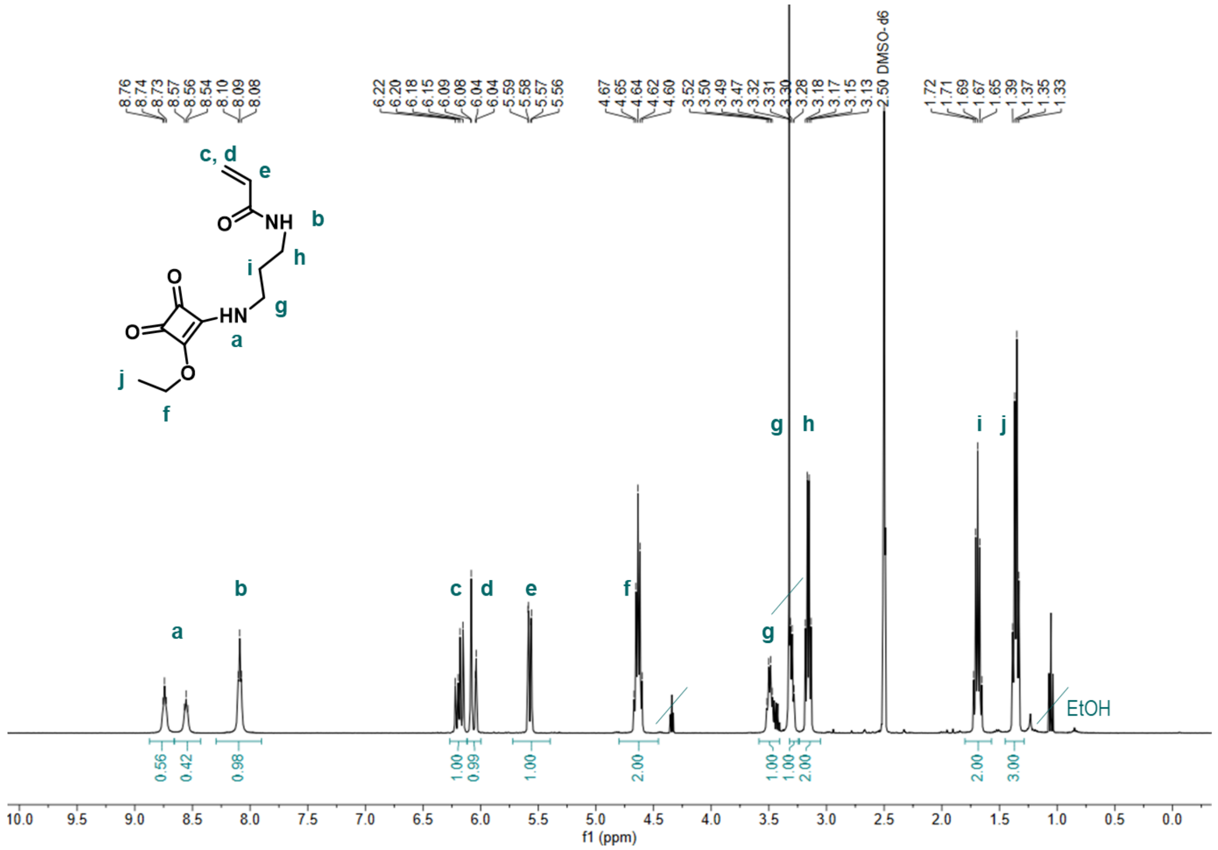

Figure S 10: ¹H-NMR spectrum (400 MHz) of A-SQ in DMSO-d₆.

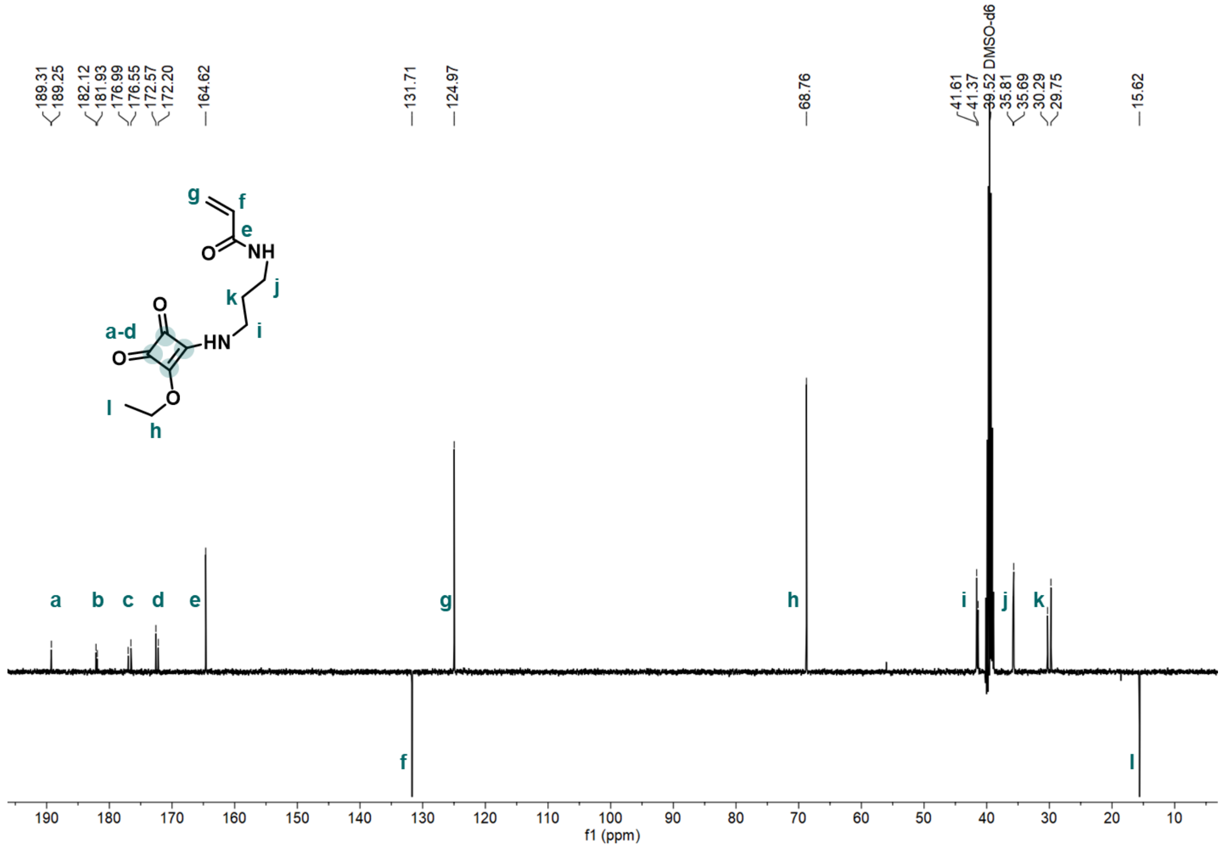

Figure S 11: APT ¹³C NMR spectrum (101 MHz) of A-SQ in DMSO-d₆.

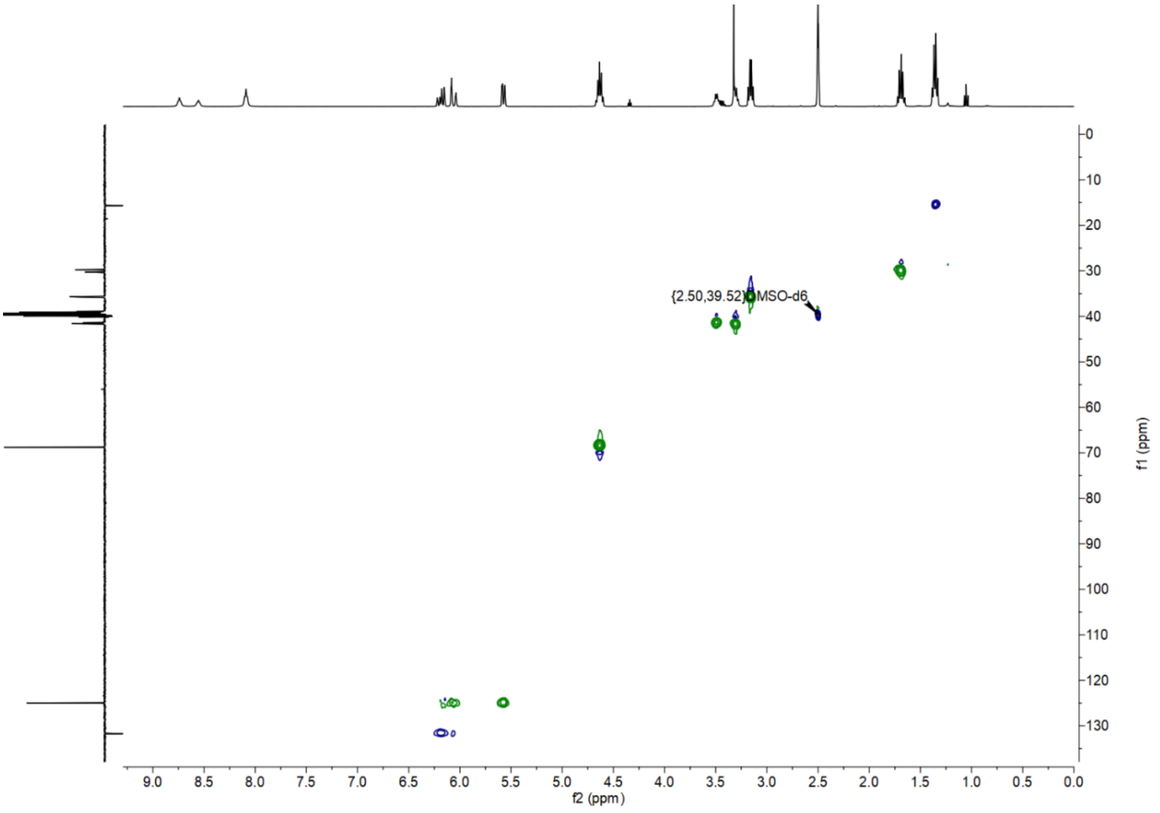

Figure S 12: 2D HSQC NMR spectrum of A-SQ in DMSO-d_6.

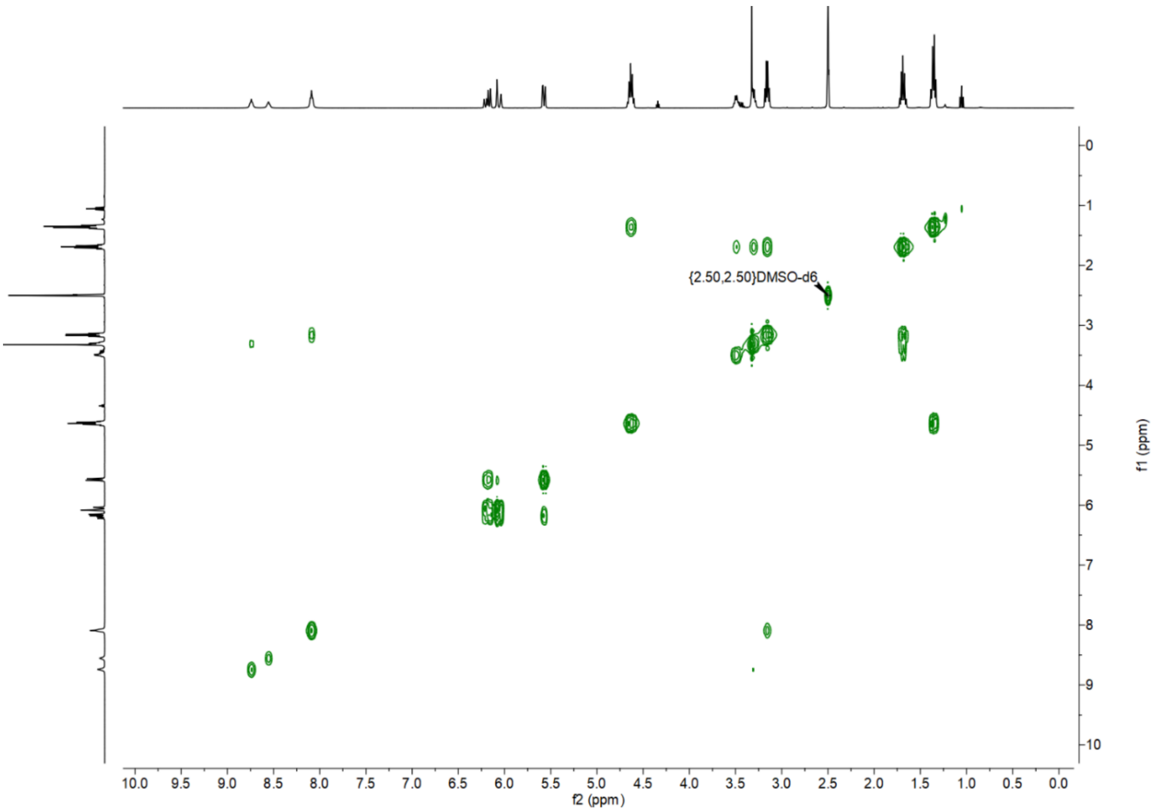

Figure S 13: 2D COSY NMR spectrum (400 MHz) of A-SQ in DMSO-d_6.

3.1.3 2-((2-ethoxy-3,4-dioxocyclobut-1-en-1-yl)amino)ethyl acrylate, Squaric ester amide acrylate (At-SQ)

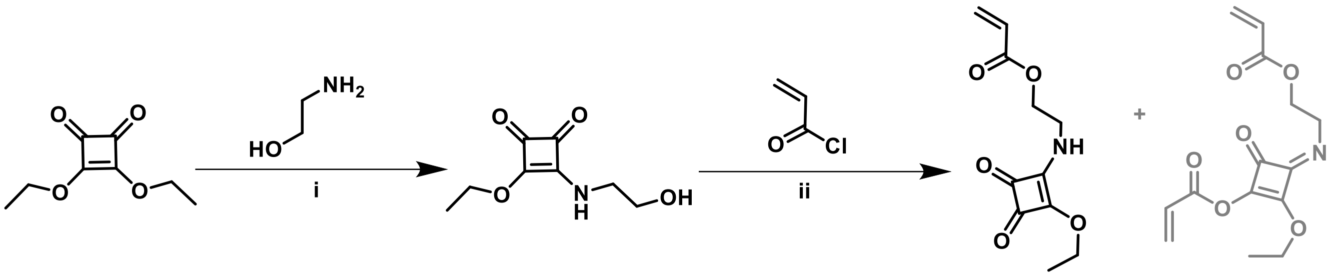

Figure S 14: Synthesis of At-SQ: i) EtOH, RT, 16 h, yield: 78 %, and ii) TEA, DCM, 0 °C, yield: 21 %.

Synthesis of 3-ethoxy-4-((2-hydroxyethyl)amino)cyclobut-3-ene-1,2-dione

In a round bottom flask, squaric acid diethyl ester (2.00 g, 1.74 mL, 11.75 mmol, 1.0 eq.) was dissolved in ethanol (50 mL). 2-Aminoethanol (0.72 g, 0.70 mL, 11.75 mmol, 1.0 eq.) was slowly added dropwise and the reaction mixture stirred for 16 h at RT. Afterwards the reaction mixture was concentrated *in vacuo* to half of its volume, poured into ice cold diethyl ether and placed overnight at -20 ºC. A colorless precipitate was formed that could be filtered off and dried *in vacuo* to yield the product as white powder (1.70 g, 78 %).

^{1}H-NMR (300 MHz, DMSO-d_6): δ (ppm) = 8.72 (br, 0.5H, **a**)[a)] ,8.56 (br, 0.5H, **a**)[a)], 4.64 (q, *J* =7.1 Hz, 2H, **b**), 3.49 (br, 3H,**c+d**)[a)], 3.33 (m, 1H, **d**)[a)], 1.36 (t, *J* = 7.1 Hz, 3H, **e**). [a)] due to rotamers a splitting of the signals is observed.

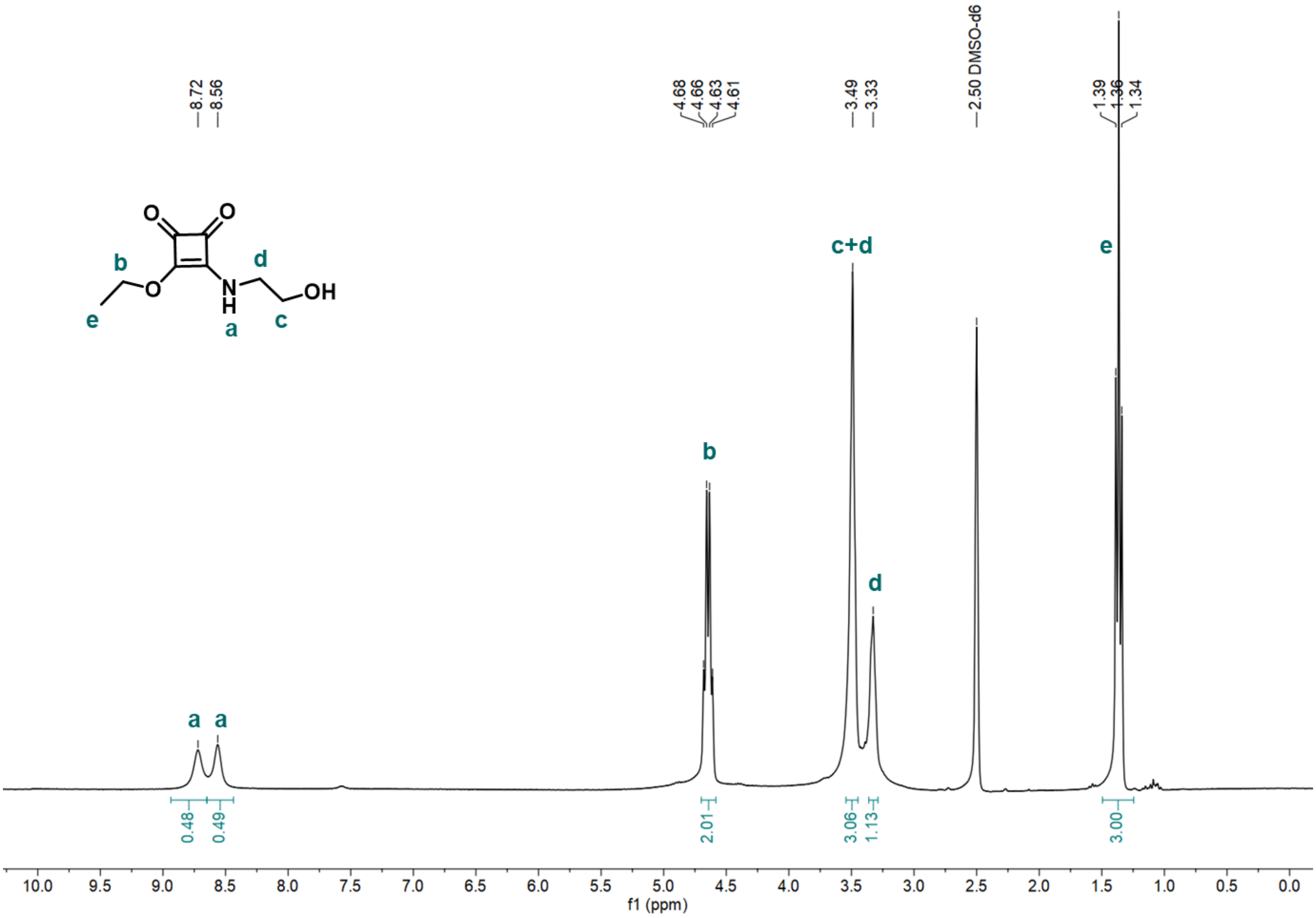

Figure S 15: ^{1}H NMR spectrum of 3-ethoxy-4-((2-hydroxyethyl)amino)cyclobut-3-ene-1,2-dione (300 MHz) in DMSO-d_6.

Synthesis of 2-((2-ethoxy-3,4-dioxocyclobut-1-en-1-yl)amino)ethyl acrylate, Squaric ester amide acrylate (At-SQ)

In an oven-dried round-bottom flask 3-ethoxy-4-((2-hydroxyethyl)amino)cyclobut-3-ene-1,2-dione (1.0 g, 5.4 mmol, 1.0 eq.) was dispersed under Argon atmosphere in anhydrous DCM (10 mL). After addition of TEA (0.83 mL, 5.9 mmol, 1.1 eq.) the solution became more clear. It was cooled for 30 min using an ice bath, before acryloyl chloride was added dropwise (0.48 mL, 5.0 mmol, 1.1 eq.). The reaction mixture was allowed to stir overnight on ice melting to water, before it was poured into water (100 mL) and the product was extracted with DCM (3x 20 mL). The combined organic layers were dried over Na_2SO_4, filtered, and concentrated *in vacuo*. Further purification of the product was achieved by silica gel chromatography using a mixed eluent of ethyl acetate and n-hexane (v:v, 1:3 to 1:1). The desired product At-SQ was obtained as colorless crystals (0.27 g, 1.14 mmol, 21%). In addition, the by-product 2-((3-(acryloyloxy)-2-ethoxy-4-oxocyclobut-2-en-1-ylidene)amino)ethyl acrylate (At-SQ-At), which was eluting faster, was afforded as yellow oil (0.39 g, 24%).

At-SQ:

^{1}H-NMR (300 MHz, Chloroform-*d*)): δ (ppm) = 7.00 (s, 0.7H, **a**)[a)] 6.43 (dd, *J* = 17.3, 1.4 Hz, 1H, **b**), 6.11 (dd, *J* = 17.3, 10.4 Hz, 1H, **c**), 5.88 (dd, *J* = 10.4, 1.4 Hz, 1H, **d**), 5.82 (s, 0.3H, **a**)[a)], 4.75 (q, *J* = 7.1 Hz, 2H, **e**), 4.39 – 4.29 (m, 2H, **f**), 3.86 (m, 2H, **g**), 1.45 (t, *J* = 7.1 Hz, 3H, **h**). [a)] due to mesomeric structures a splitting of the signals is observed.

^{13}C-NMR (75 MHz, Chloroform-*d*): δ (ppm) = 177.95 (**a**), 172.80 (**b**), 165.88 (**c**), 132.02 (**d**), 127.80 (**e**), 70.07 (**f**), 63.39 (**g**), 43.88 (**h**), 15.94 (**i**).

At-SQ-At:

^{1}H-NMR (300 MHz, Chloroform-*d*)): δ (ppm) = 6.70 (dd, J = 16.5, 10.1 Hz, 1H, **a**), 6.53 (dd, J = 16.6, 1.7 Hz, 1H, **b**), 6.32 (dd, J = 17.2, 1.5 Hz, 1H, **c**), 6.01 (dd, J = 17.2, 10.4 Hz, 1H, **d**), 5.93 (dd, J = 10.1, 1.8 Hz, 1H, **e**), 5.83 (dd, J = 10.4, 1.5 Hz, 1H, **f**), 4.85 (q, J = 7.1 Hz, 2H, **g**), 4.40 (t, J = 5.2 Hz, 2H, **h**), 4.28 (t, J = 5.2 Hz, 2H, **i**), 1.48 (t, J = 7.1 Hz, 3H, **j**).

^{13}C-NMR (75 MHz, Chloroform-*d*): δ (ppm) = 188.26 (**a**), 186.55 (**b**), 183.74 (**c**), 171.91(**d**), 165.55(**e**), 164.77(**f**), 132.59 (**g**), 131.87 (**h**), 127.78 (**i**), 127.77 (**j**), 71.70 (**k**), 62.45(**l**), 45.23 (**m**), 15.84(**n**).

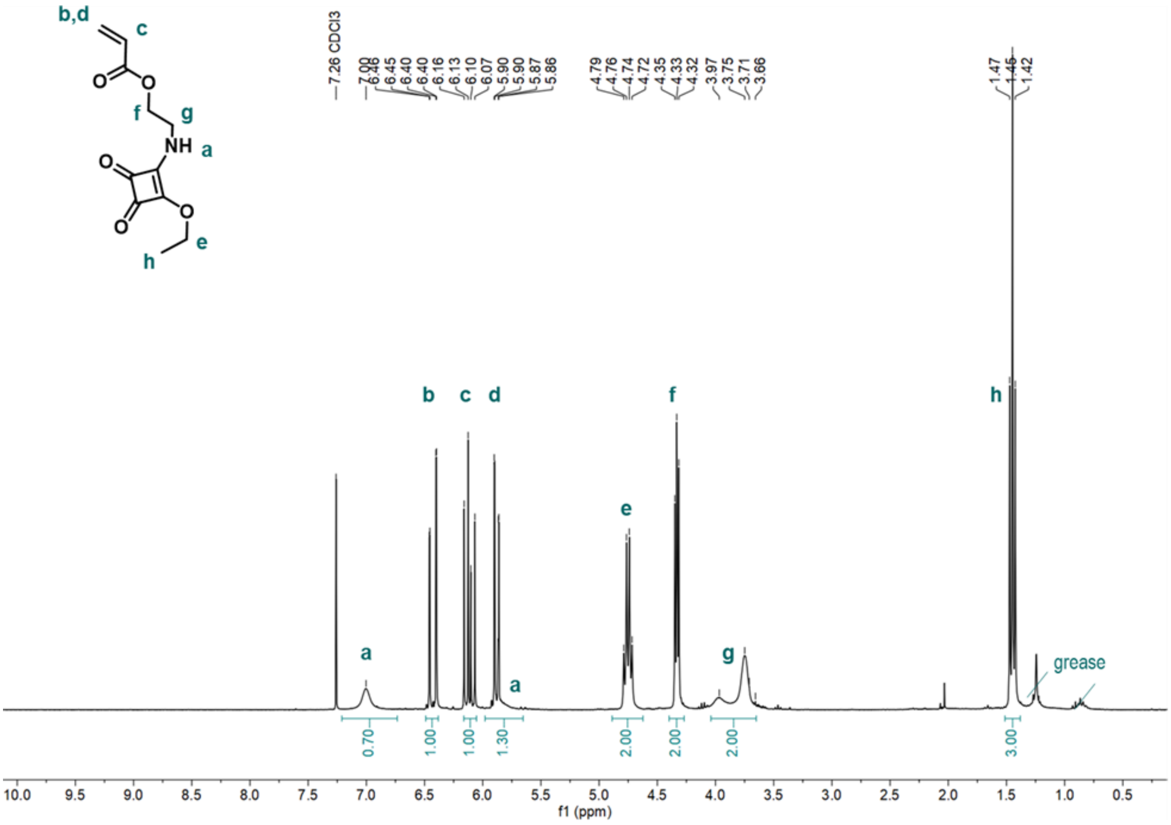

Figure S 16: ^{1}H NMR spectrum of At-SQ (300 MHz) in $CDCl_3$.

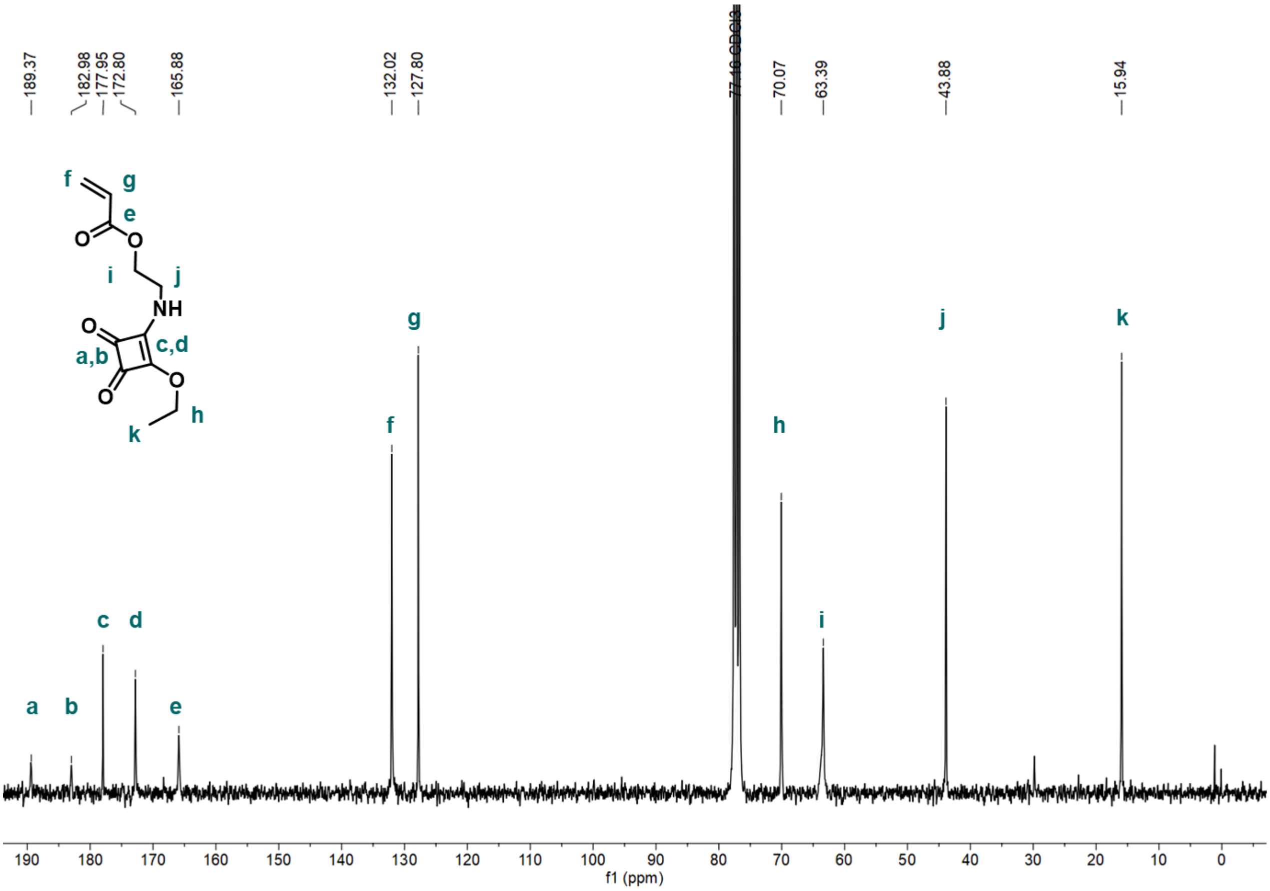

Figure S 17: ^{13}C NMR spectrum of At-SQ (75 MHz) in $CDCl_3$.

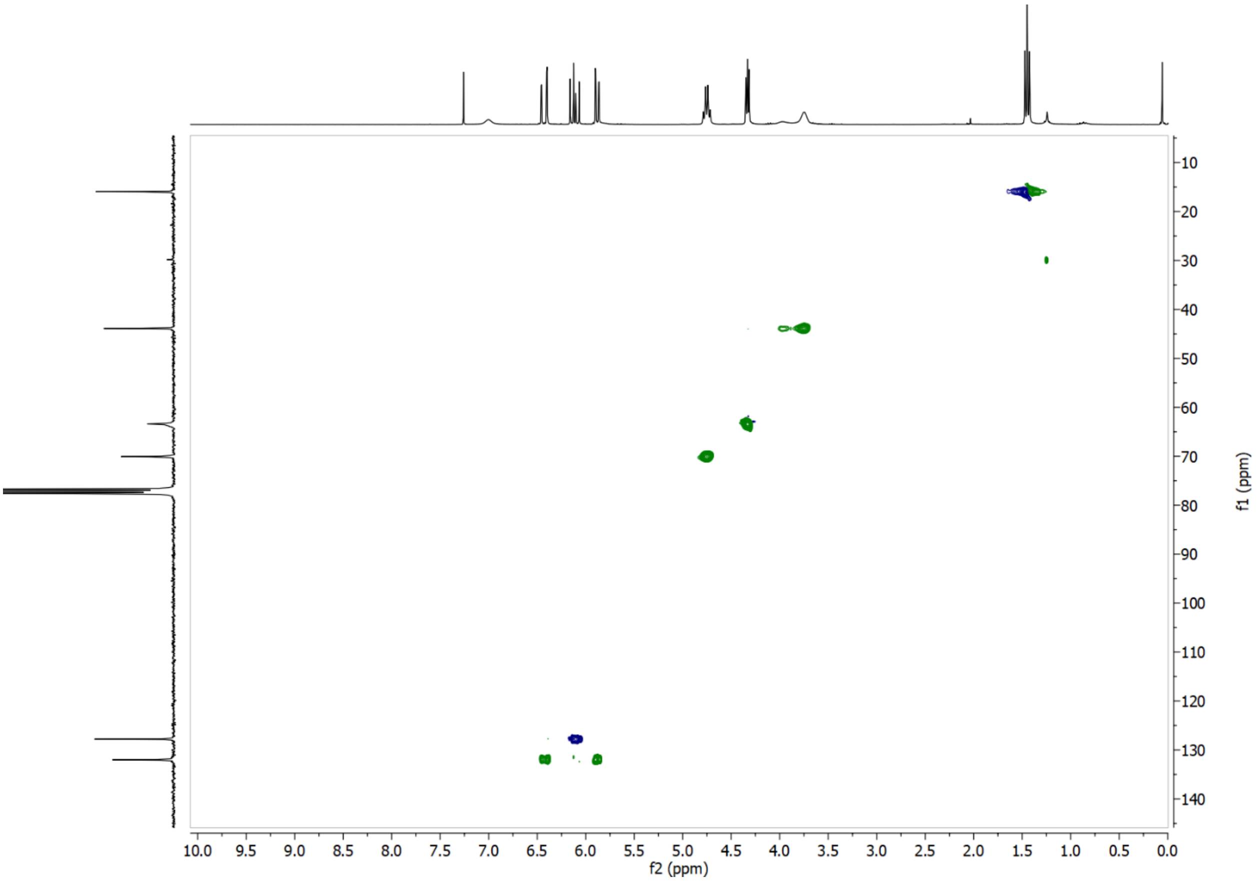

Figure S 18: 2D HSQC NMR spectrum of At-SQ in $CDCl_3$.

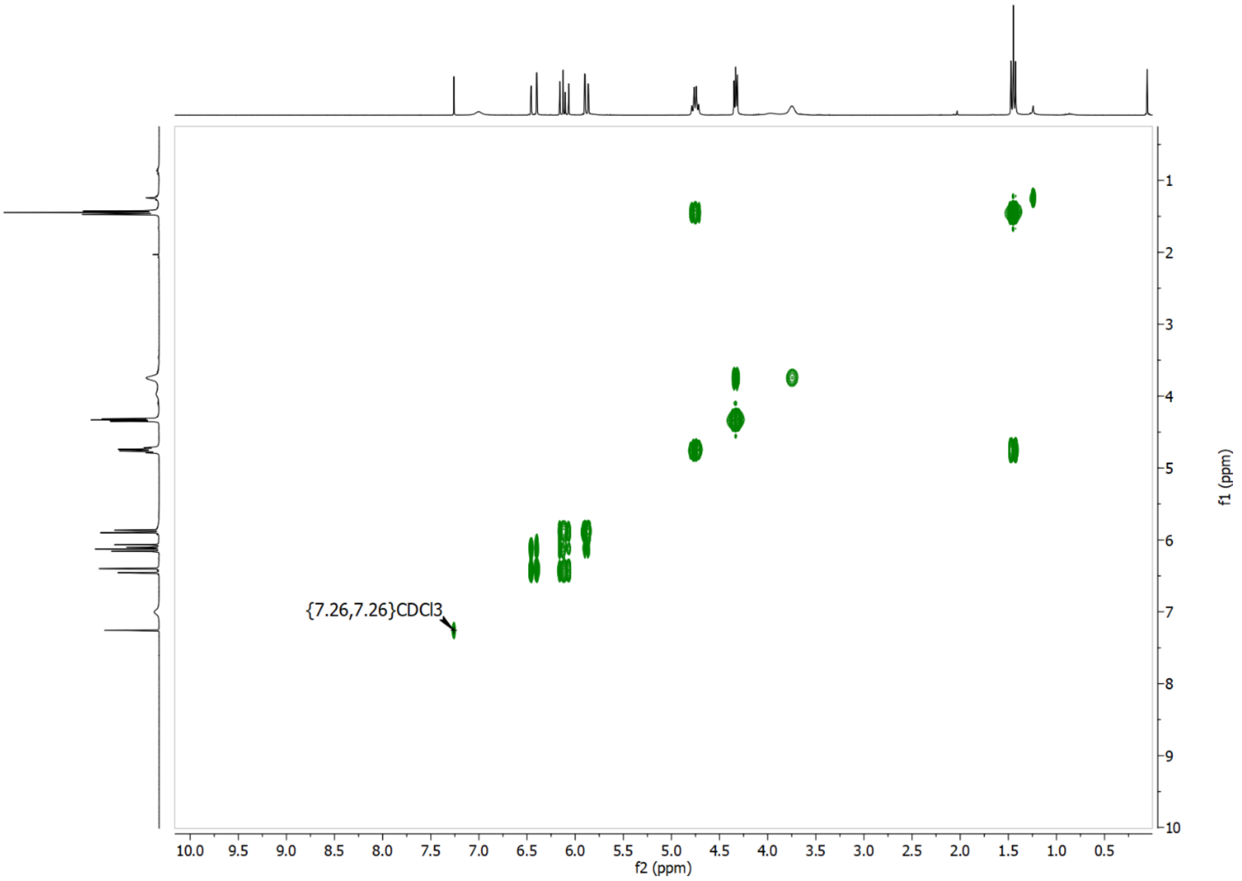

Figure S 19: 2D COSY NMR spectrum (300 MHz) of SQ-At in $CDCl_3$.

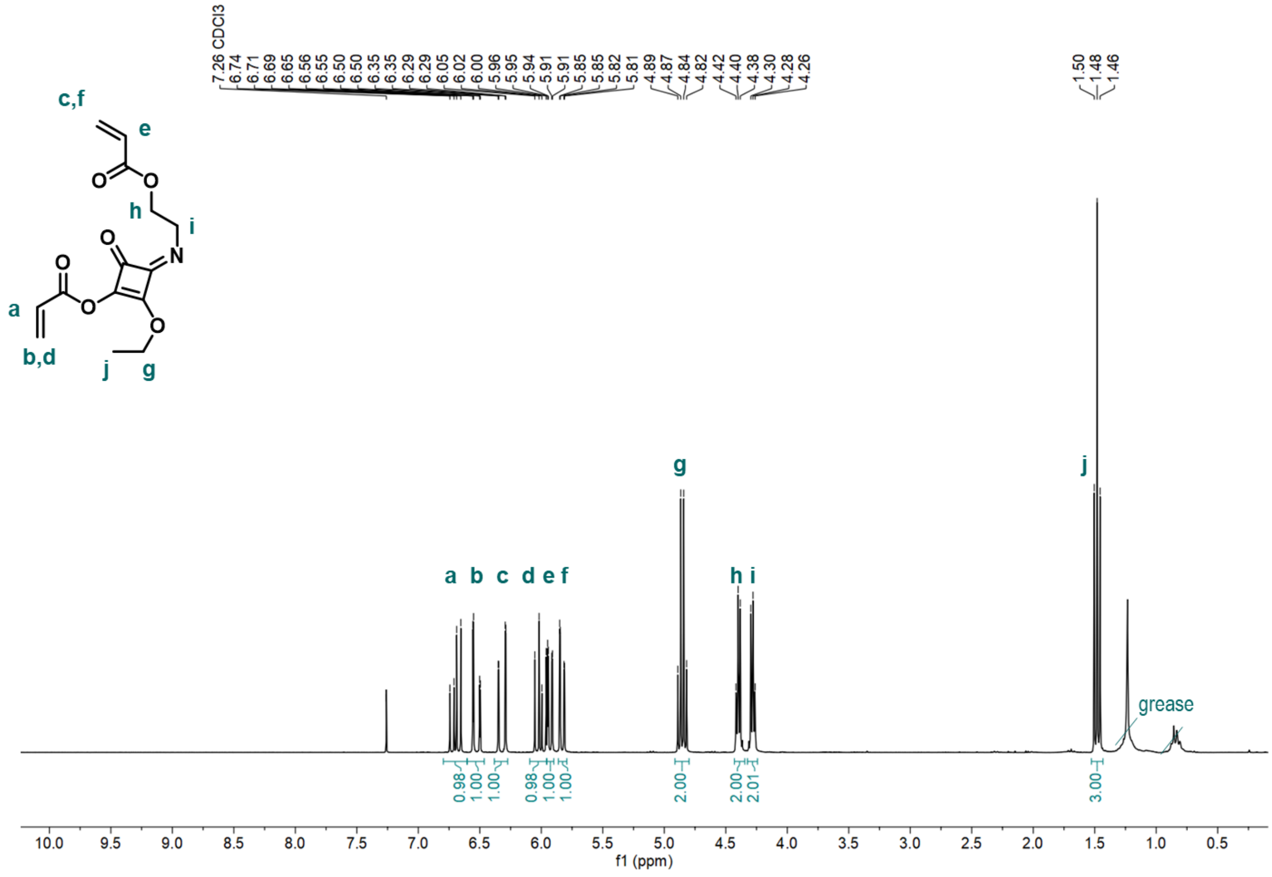

Figure S 20: ^{1}H NMR spectrum of At-SQ-At (by-product) (300 MHz) in $CDCl_3$.

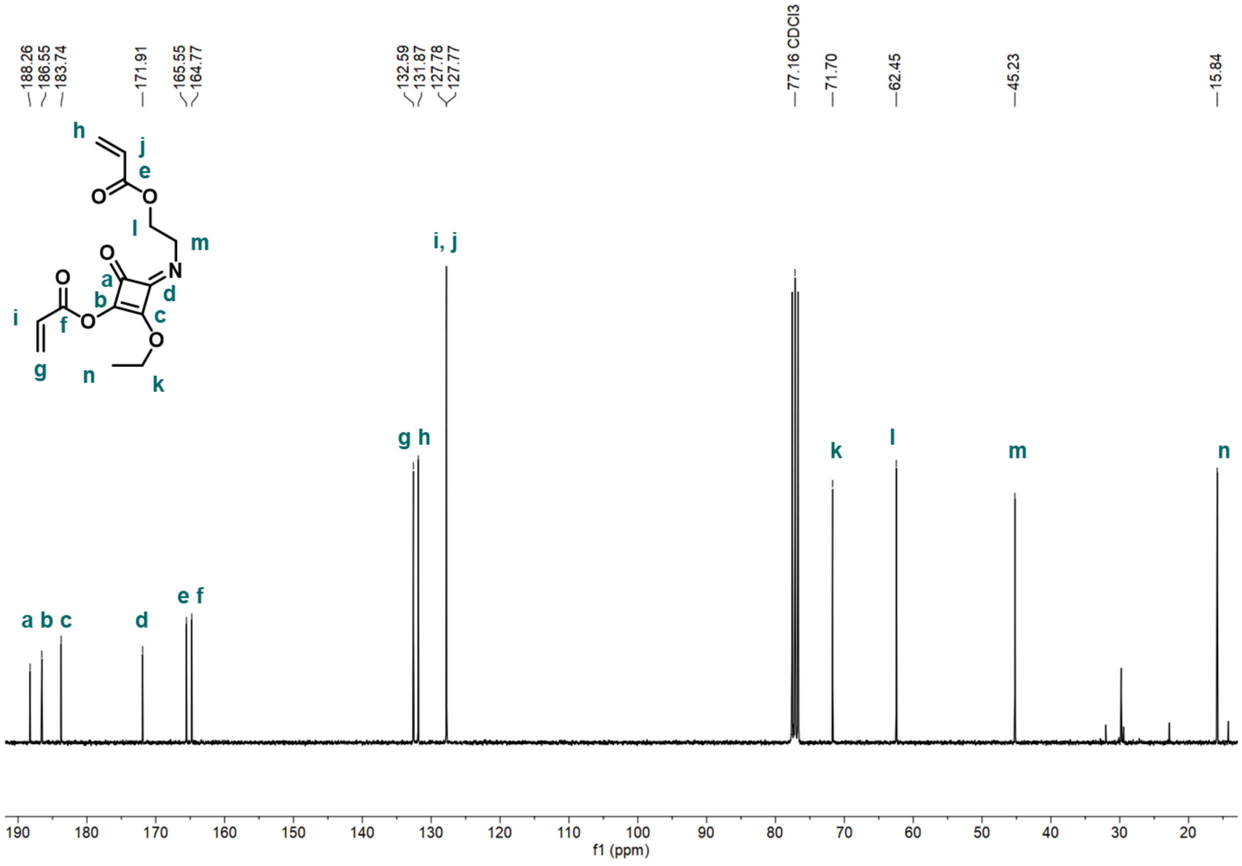

Figure S 21: ^{13}C NMR spectrum of At-SQ-At (by-product) (75 MHz) in $CDCl_3$.

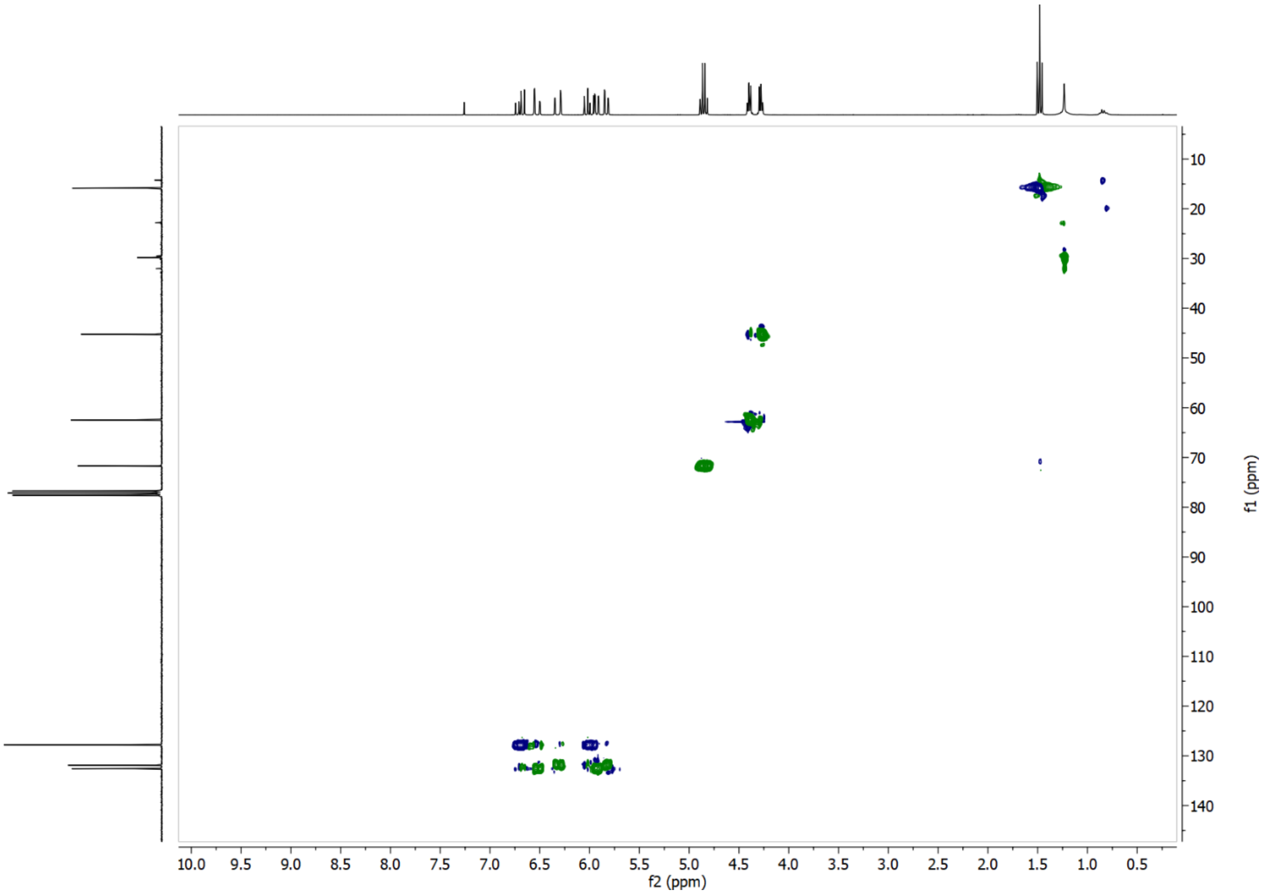

Figure S 22: 2D HSQC NMR spectrum of At-SQ-At (by-product) in $CDCl_3$.

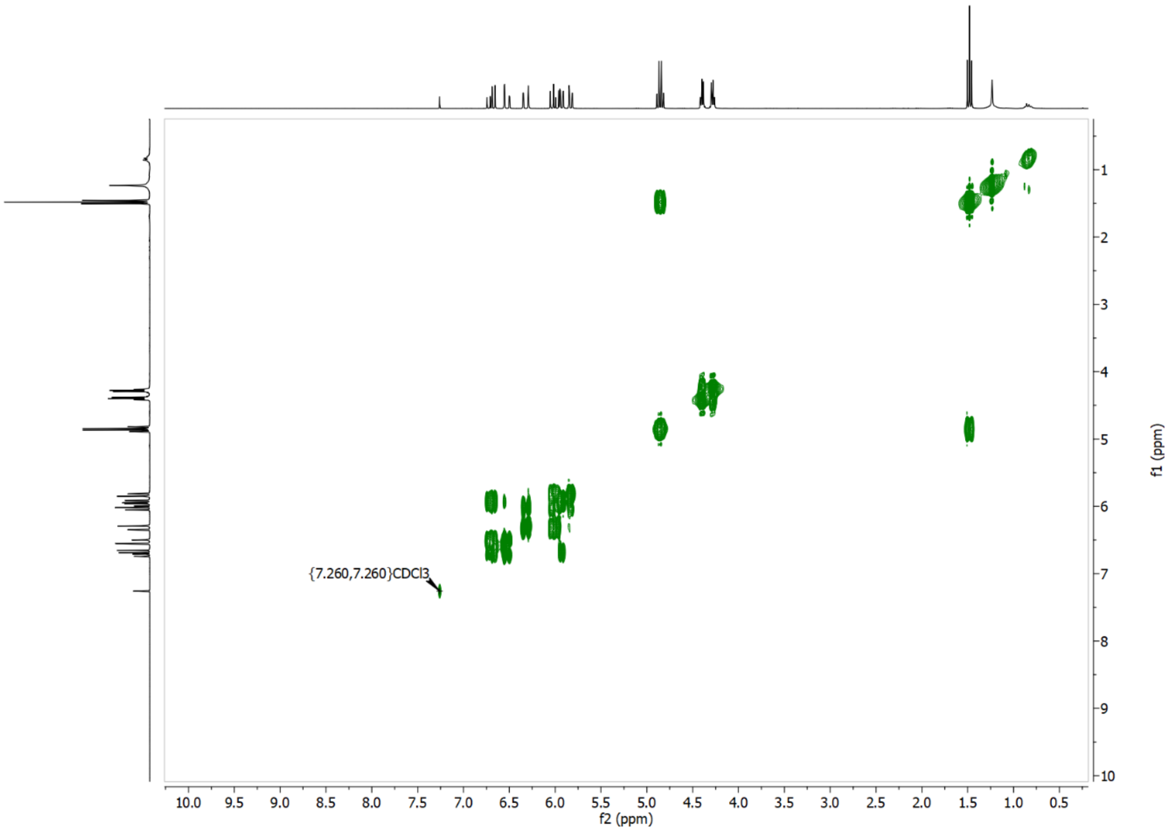

Figure S 23: 2D COSY NMR spectrum of At-SQ-At (by-product) in $CDCl_3$.

3.2 Synthesis of macro-trithiocarbonate-chain transfer agent (macro-TTC-CTA)

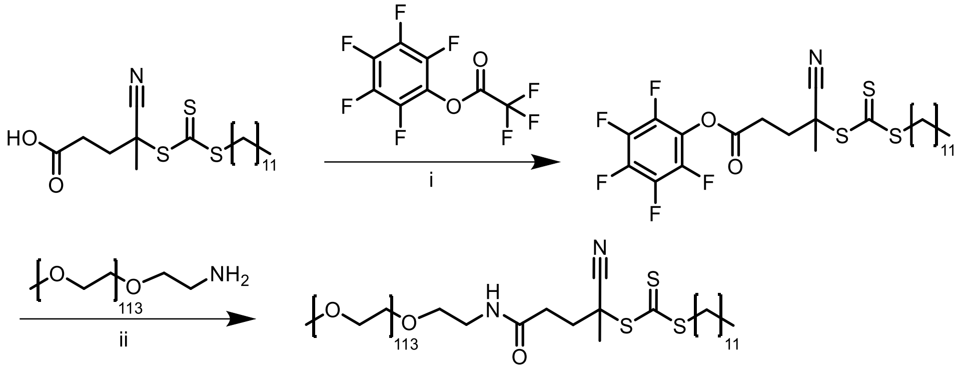

Figure S 24: Synthesis of poly(ethylene glycol)-trithiocarbonate-CTA, i) TEA, DCM, 0 °C, 3 h, yield: 77 %, ii) TEA, DCM, 0 °C, 16 h, yield: 95 %.

Synthesis of pentafluorophenyl-trithiocarbonate chain transfer agent (PFP-TTC-CTA) (i)

In an oven-dried round bottom flask 4-cyano-4-[(dodecylsulfanylthiocarbonyl)sulfanyl]pentanoic acid (TTC-CTA, 1.00 g, 2.5 mmol, 1 eq.) was dissolved under Argon atmosphere in anhydrous DCM (25 mL) and TEA (0.75 mg, 7.4 mmol, 2.5 eq.). The solution was cooled for 30 min using an ice bath and under protection from light, before pentafluorophenyl trifluoroacetate (1.74 g, 8.2 mmol, 2.5 eq.) was added dropwise *via* syringe over 45 min. The reaction mixture was allowed to stir for another 3 h on ice, before the reaction mixture was diluted with DCM (10 mL) washed with water (25 mL). The obtained aqueous layer was extracted with DCM (30 mL). The combined organic layers were dried over $MgSO_4$, filtered, and concentrated *in vacuo*. Further purification of the product was achieved by silica gel chromatography using a mixed eluent of petroleum ether and ethyl acetate (15:1). The product PFP-TTC-CTA was obtained as orange oil (1.10 g, 77 %).

^{1}H-NMR (300 MHz, Chloroform-*d*): δ (ppm) = 3.34 (t, 2H, **a**), 3.06-2.99 (m, 2H, **b**), 2.71-2.64 (m, 1H, **c**), 2.55-2.48 (m,1H, **d**), 1.93 (s, 3H, **e**), 1.72 – 1.68 (m, 2H, **f**), 1.43 – 1.38 (m, 2H, **g**), 1.30-1.26 (m, 16H, **h**), 0.88 (s, 3H, **i**).

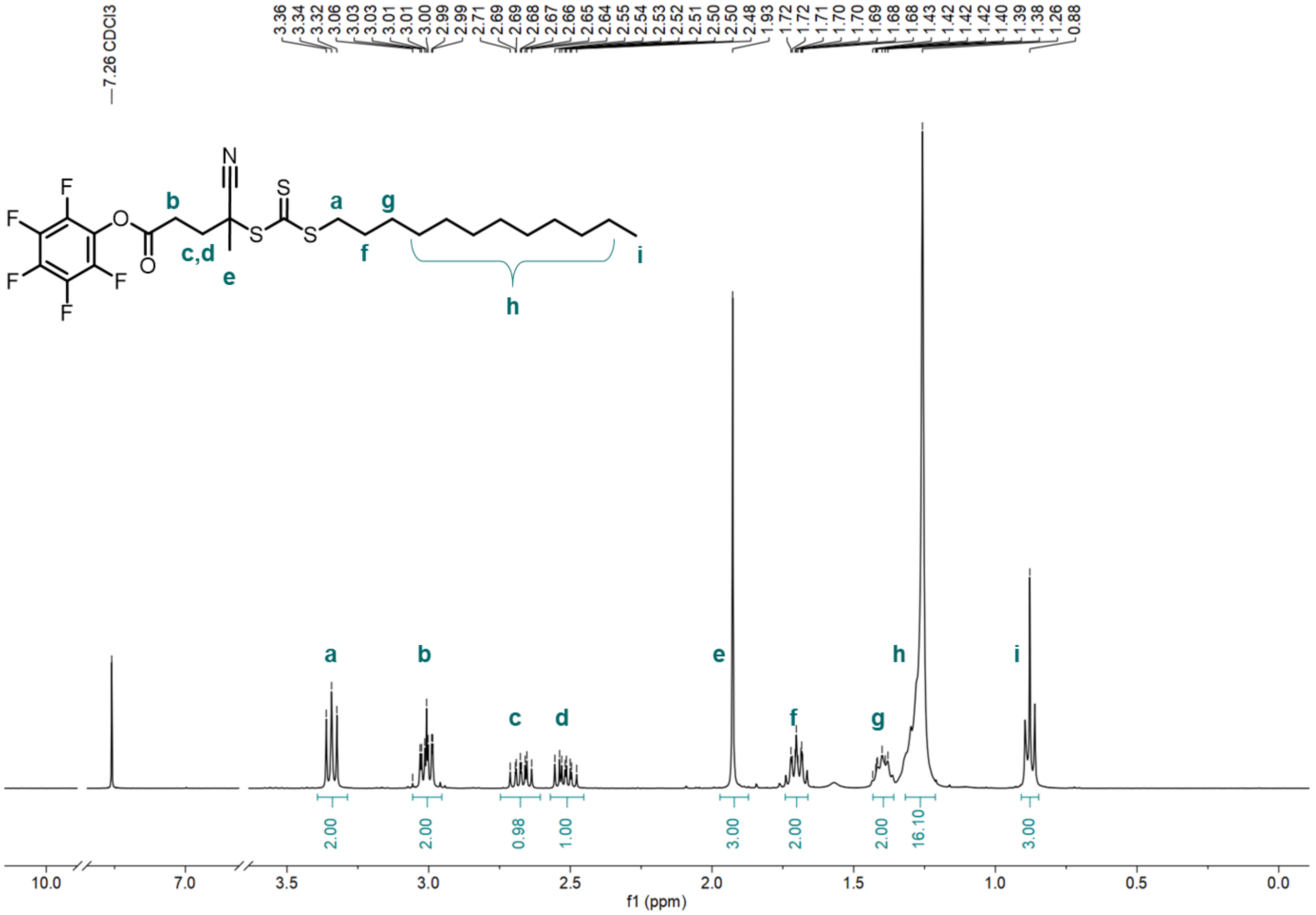

Figure S 25: ^{1}H-NMR spectrum (300 MHz) of PFP-TTC-CTA in $CDCl_3$.

Synthesis of Poly(ethylene glycol)-Trithiocarbonate-CTA, PEG-TTC-CTA (ii)

PFP-TTC-CTA (0.57 g, 1.0 mmol, 5 eq.) was dissolved in an oven-dried round bottom flask under Argon atmosphere in anhydrous DCM (5 mL). This solution was stirred for 15 min using an ice bath and under protection from light, before methoxypoly(ethylene glycol)amine (mPEG-NH_2, M_n: 5 kDa) (1.00 g, 0.20 mmol, 1 eq.) dissolved in anhydrous DCM (10 mL) and TEA (0.10 g, 1.00 mmol, 5 eq.) was added over 70 min forming an intense orange solution. The mixture was stirred for 16 h on ice melting to water. The reaction mixture was concentrated *in vacuo* to 7 mL, before being precipitated three times in cold diethyl ether (- 20 °C). To remove remaining pentafluorophenyl-TEA salts the precipitated PEG-TTC-CTA was dissolved in millipore water (40 mL) and dialyzed for 24 h against Millipore water. Finally, the PEG-TTC-CTA was isolated by lyophilization as yellowish powder (1.03 g, 95 %).

^{1}H-NMR (400 MHz, Chloroform-*d*): δ (ppm) = 6.42-6.40 (m, 1H, **a**), 3.82 – 3.79 (m, 2H, **b**), 3.66-3.61 (m, 497H, **c**), 3.47 – 3.42 (m, 2H, **d**), 3.37 (s, 3H, **e**), 3.33 – 3.29 (m, 2H, **f**), 2.51 – 2.34 (m, 4H, **g**), 1.88 (s, 3H, **h**), 1.71-1.64 (m, 2H, **i**), 1.38-1.35 (m, 2H, **j**), 1.25-1.24 (m, 16H, **k**), 0.88-0.85 (m, 3H, **l**).

SEC (DMAc, PMMA-Std.) M_n = 10,013 g/mol, M_w = 10,384 g/mol, PDI = 1.04.

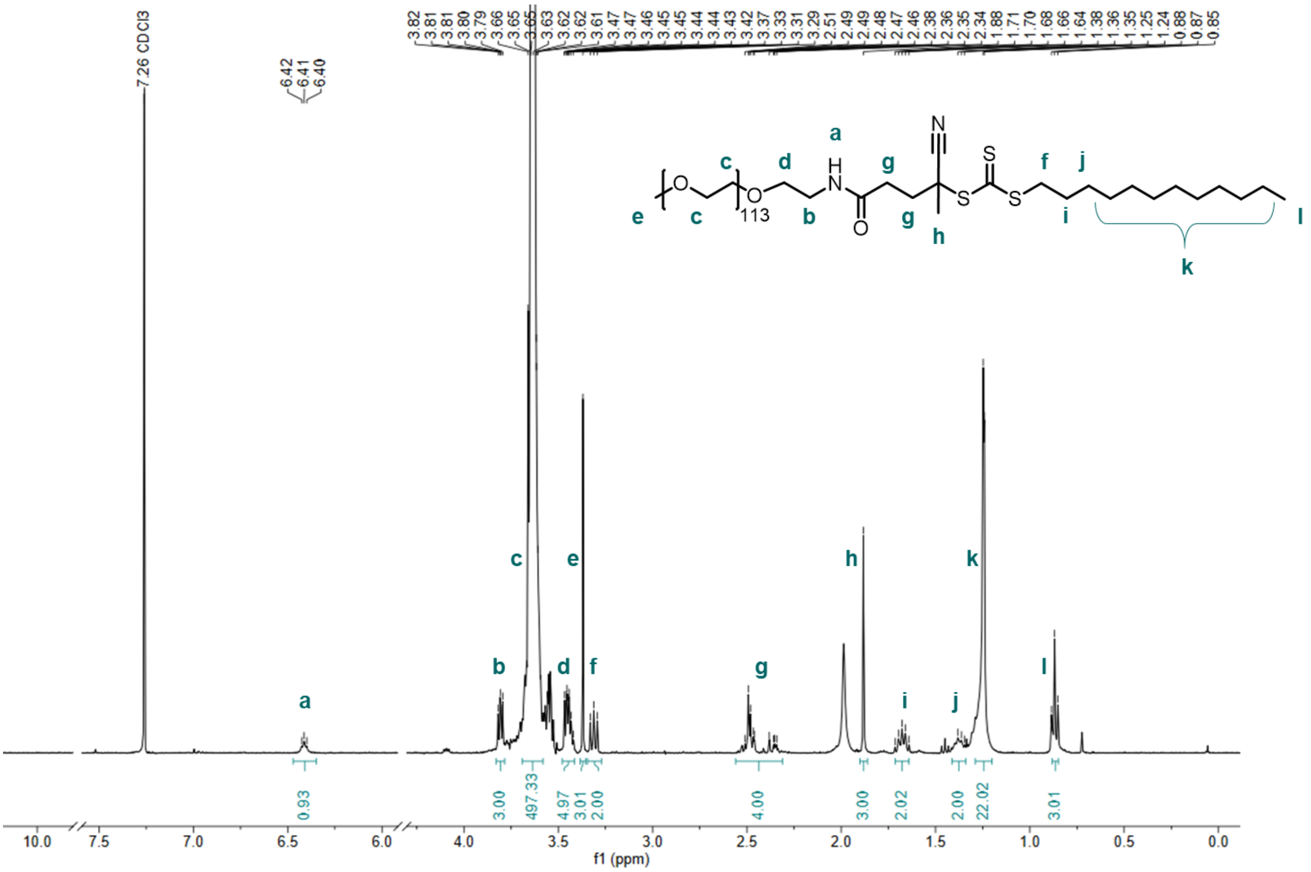

Figure S 26: ^{1}H-NMR spectrum (300 MHz) of PEG-TTC-CTA in $CDCl_3$.

3.3 Block copolymerization of MA-SQ with PEG-TTC-CTA

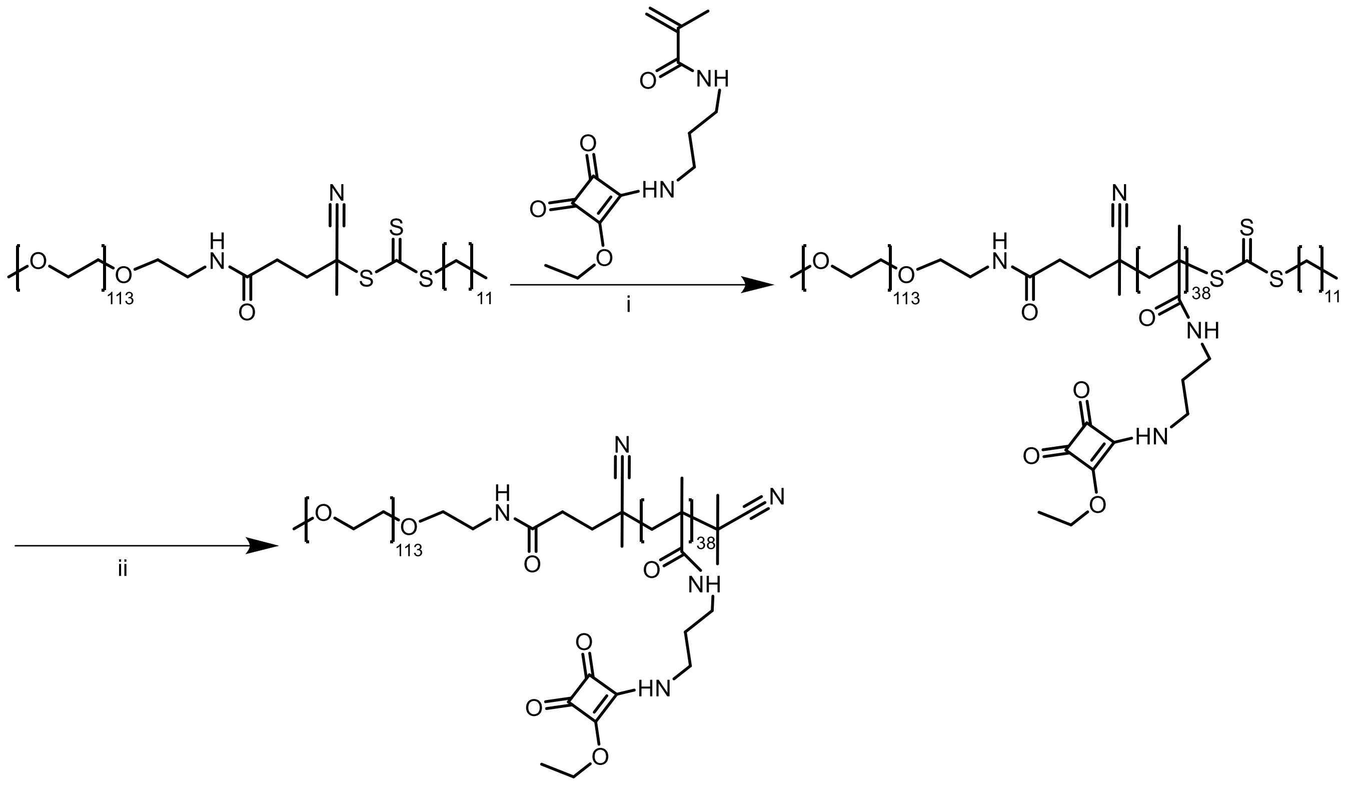

Figure S 27: Synthesis of block copolymer mPEG113-b-p(MA-SQ)38 with macro-PEG-TTC-CTA, i) AIBN, DMF, 70 °C, 45 h, yield: quantitative, ii) excess AIBN, DMF, 70 °C, 12 h, yield: 97%.

Block copolymerization (i)

A typical block copolymerization of MA-SQ with a theoretical degree of polymerization (DP) of 50 was performed using the macro-CTA in a Schlenk tube equipped with a stir bar. The reaction vessel was loaded with AIBN (1.0 mg, 7.0 μmol, 0.2 eq.), macro-CTA (180.0 mg, 33.0 μmol, 1.0 eq.), and MA-SQ (443.8 mg, 1.67 mmol, 50 eq.), which were subsequently dissolved in anhydrous pre-degassed DMF (1.7 mL). After three freeze-pump-thaw cycles the tube, while being in its evacuated state, was immersed in an oil bath at 70 °C for 45 h. After the reaction time, ^{1}H-NMR indicated a monomer conversion of 78 %. The block copolymer was precipitated three times in cold diethyl ether (-20 °C), isolated by subsequent centrifugation, and dried under high vacuum for 14 h affording mPEG$_{113}$-*b*-p(SQ-MA)$_{38}$-CTA (526.2 mg, quantitative) as yellow solid.

SEC (DMAc, PMMA-Std.): M_n = 22,183 g/mol, M_w = 25,830 g/mol, PDI = 1.16.

Removal of trithiocarbonate end group (ii)

In a Schlenk tube equipped with a stir bar mPEG$_{113}$-p(SQ-MA)$_{38}$-CTA (526.2 mg, 33.3 μmol, 1 eq.) and AIBN (274.4 mg ,1.670 mmol, 50 eq.) were dissolved under argon atmosphere in pre-degassed DMF (2.5 mL). The tube was immersed in an oil bath at 70 °C for 12 h. The resulting polymer was isolated by threefold precipitation into cold diethyl ether (-20 °C) and centrifugation. Finally, the polymer was dried under high vacuum for 13 h affording mPEG$_{113}$-*b*-p(SQ-MA)$_{38}$ (491.2 mg, 32.0 μmol, 97 %) as yellow solid.

^{1}H-NMR (400 MHz, DMSO-d_6): δ (ppm) = 8.72 (s, 0.5H, **a**)[a)] 8.56 (s, 0.5H, **a**)[a)], 7.30 (s, 1H, **b**), 4.65 (s, 2H, **c**), 3.51 (s, 14H, **d**), 3.46-3.40 (m, 1H, **e**)[a)], 3.32-3.26 (m, 1H, **e**)[a)],2.99 (m, 2H (1.7+0.2H), **f**), 1.62-0.78 (m, 10H, **g**). [a)] due to rotamers a splitting of the signals is observed.

SEC (DMAc, PMMA-Std.): M_n = 20,207 g/mol, M_w = 25,951 g/mol, PDI = 1.28.

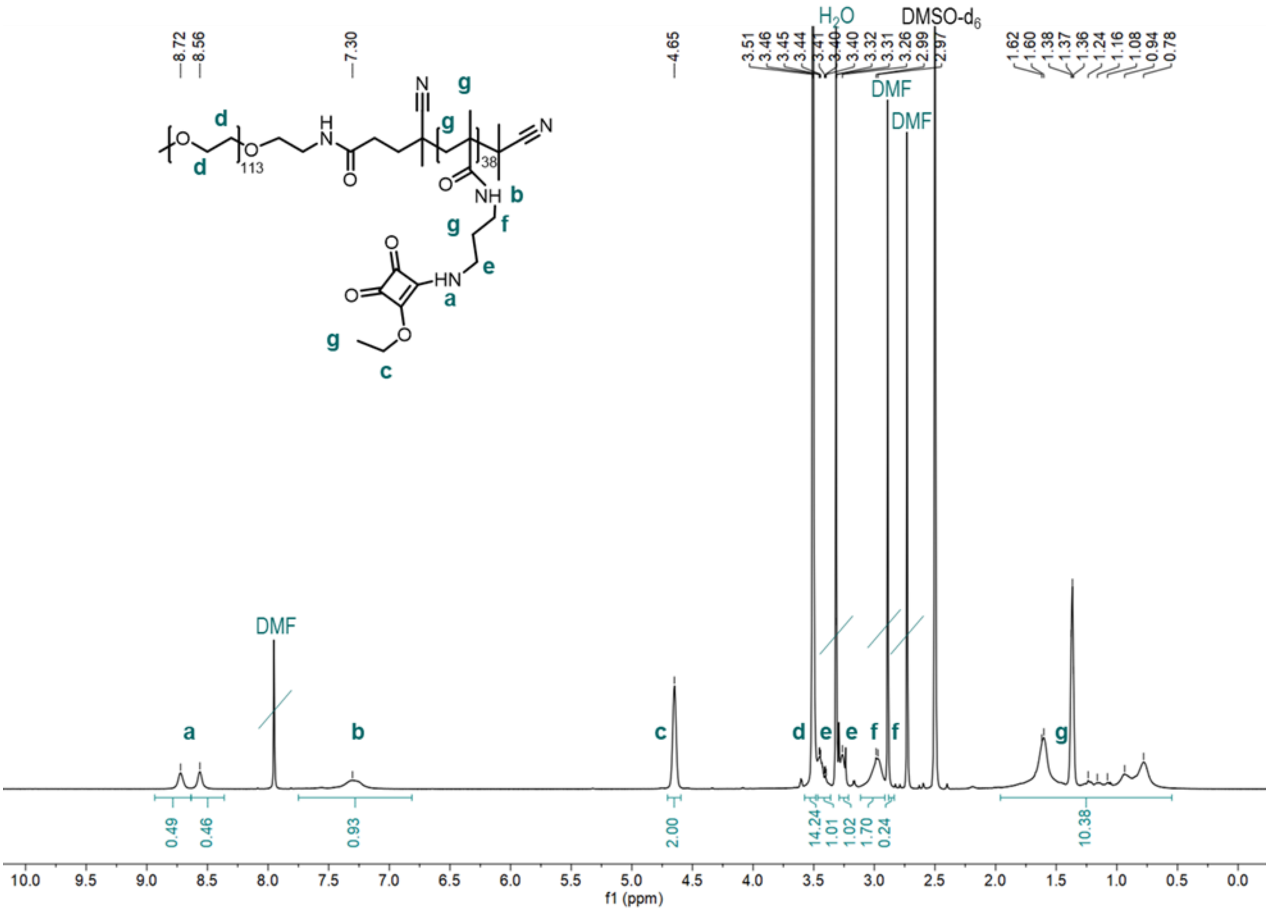

Figure S 28: ^{1}H-NMR (300 MHz) spectrum of mPEG$_{113}$-b-p(MA-SQ)$_{38}$ in DMSO-d$_6$.

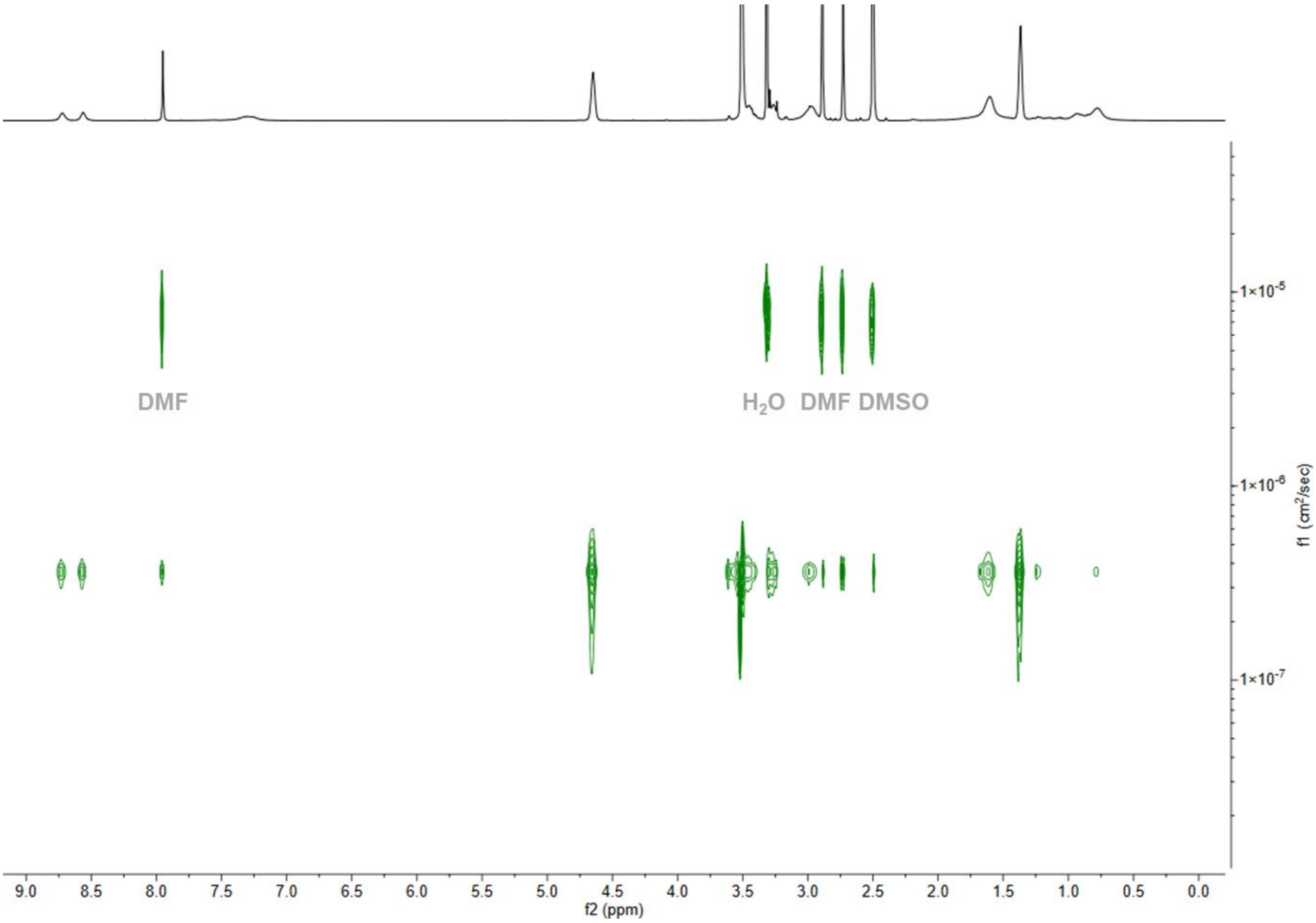

Figure S 29: DOSY spectrum of mPEG$_{113}$-b-p(MA-SQ)$_{38}$ in DMSO-d$_6$.

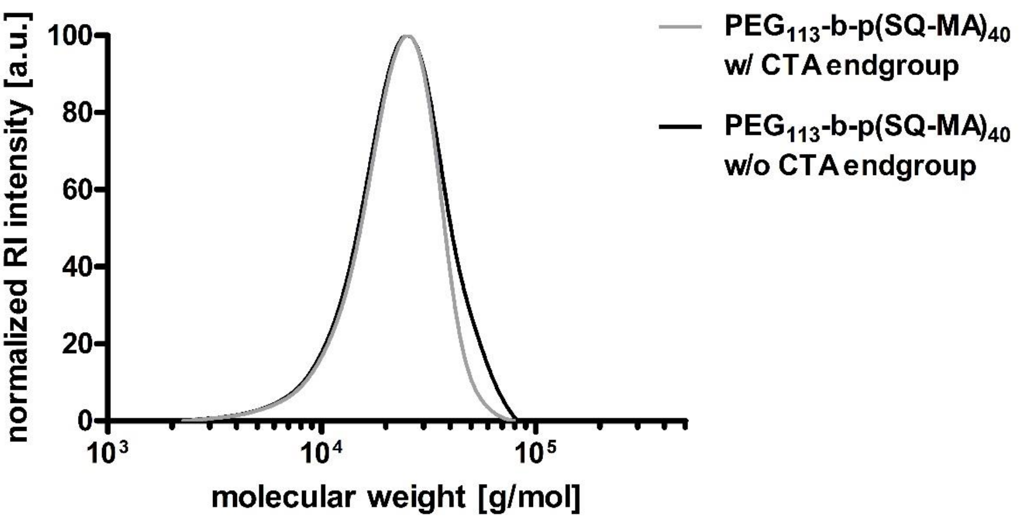

Figure S 30: Molecular weight distributions of block copolymer mPEG$_{113}$-p(SQ-MA)$_{38}$ before (grey) and after RAFT end group removal (black) determined by SEC ((DMAc, PMMA-Std).

3.4 Homopolymerization of MA-SQ with TTC-CTA

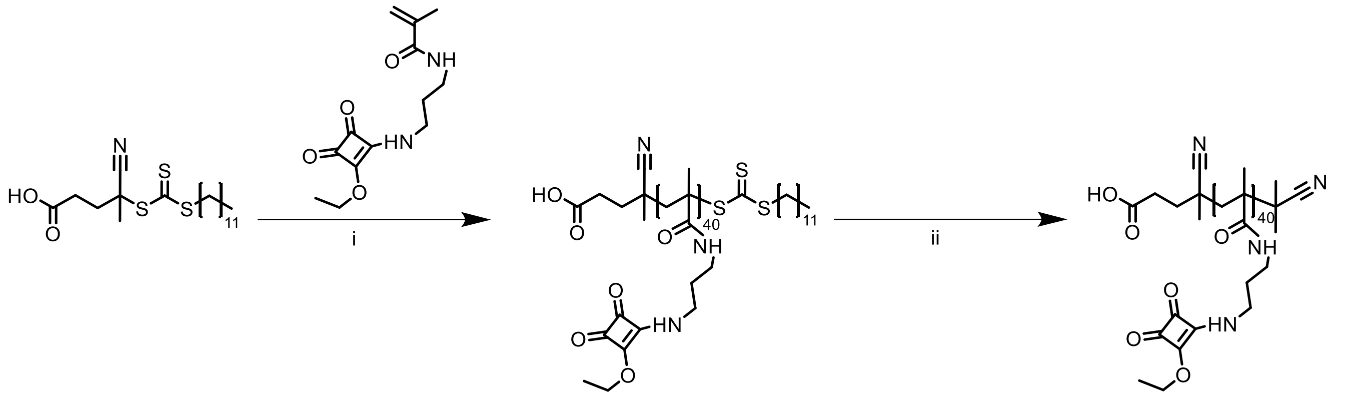

Figure S 31: Synthesis of homopolymer p(MA-SQ)$_{40}$ with TTC-CTA i) AIBN, DMF, 70 °C, 18 h, yield: quantitative, ii) excess AIBN, DMF, 70 °C, 12 h, yield: 97 %.

For a typical homopolymerization with a DP of 50, MA-SQ (533.0 mg, 2.0 mmol, 50 eq.), TTC-CTA (16.1 mg, 40.0 µmol, 1.0 eq.), and AIBN (1.3 mg, 8 µmol, 0.2 eq.) were dissolved in 2 mL anhydrous pre-degassed DMF. The solution was degassed by three freeze-pump-thaw cycles, before it was immersed in an oil bath at 70 °C for 37 h, while being in its evacuated state. After the reaction time,^{1}H-NMR analysis indicated a monomer conversion of 79 %. The homopolymer was precipitated three times in cold diethyl ether (-20 °C), isolated by subsequent centrifugation, and dried under high vacuum for 16 h affording p(SQ-MA)$_{40}$-CTA (440.2 mg, 40.2 µmol, quantitative) as yellow solid.

SEC (DMAc, PMMA-Std.): M_n =13,764 g/mol, M_w = 17,296 g/mol, PDI = 1.26.

For free radical polymerization conditions, the homopolymerization was proceeded analogously without TTC-CTA and with increased equivalents of the radical initiator AIBN (5.7 mg, 40 µmol, 1 eq.).

SEC (DMAc, PMMA-Std.): M_n = 47,986 g/mol, M_w = 108,804 g/mol, PDI = 2.27.

Removal of trithiocarbonate end group:

Analogously to the previously described end group removal, the homopolymer p(SQ-MA)$_{40}$-CTA (440.2 mg, 40.2 µmol, 1 eq.) and AIBN (331.0 mg, 2.02 mmol, 50 eq.) were dissolved under argon atmosphere in pre-degassed DMF (1.5 mL). The tube was immersed in an oil bath at 70 °C for 12 h. The resulting polymer was isolated by threefold precipitation into cold diethyl ether (-20 °C) and centrifugation. After the polymer was dried under high vacuum for 14 h, p(SQ-MA)$_{40}$ (425.3 mg, 39.2 µmol, 97 %) was obtained as yellow solid.

^{1}H-NMR (300 MHz, DMSO-*d$_6$*): δ (ppm) = 8.73 (s, 0.5H, **a**)[a)], 8.57 (s, 0.5H, **a**)[a)], 7.31 (s, 1H, **b**), 4.66-4.64 (m, 2H, **c**), 3.45 (m, 1H, **d**)[a)], 3.27 (m, 1H, **d**)[a)], 3.01-2.71 (m, 2H, **e**), 1.62-0.78 (m, 10H, **f**). [a)]due to rotamers a splitting of the signals is observed.

SEC (DMAc, PMMA-Std.): M_n =13,757 g/mol, M_w = 17,892 g/mol, PDI = 1.30.

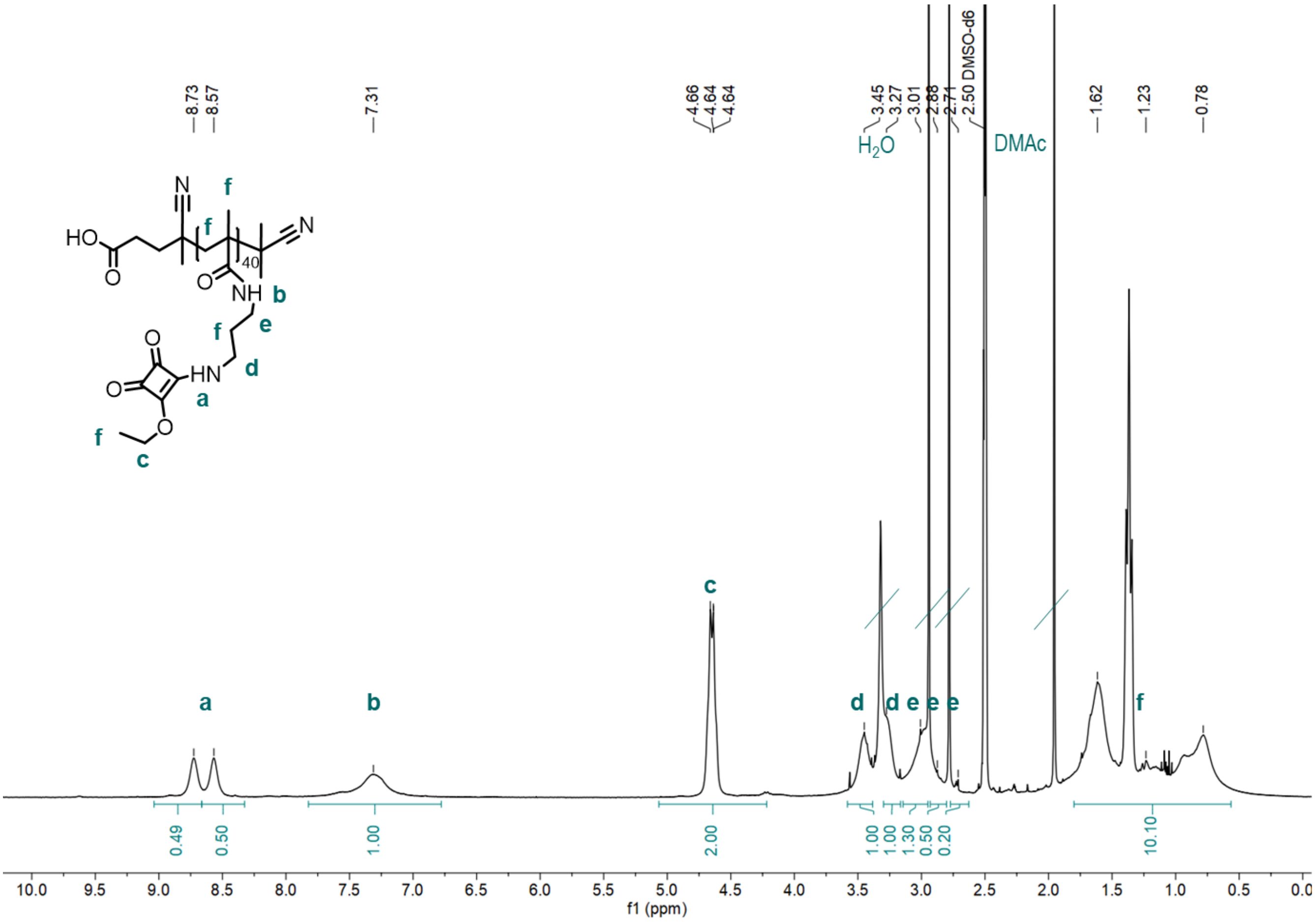

Figure S 32: ^{1}H-NMR (300 MHz) spectrum of p(MA-SQ)$_{38}$ in DMSO-d$_6$.

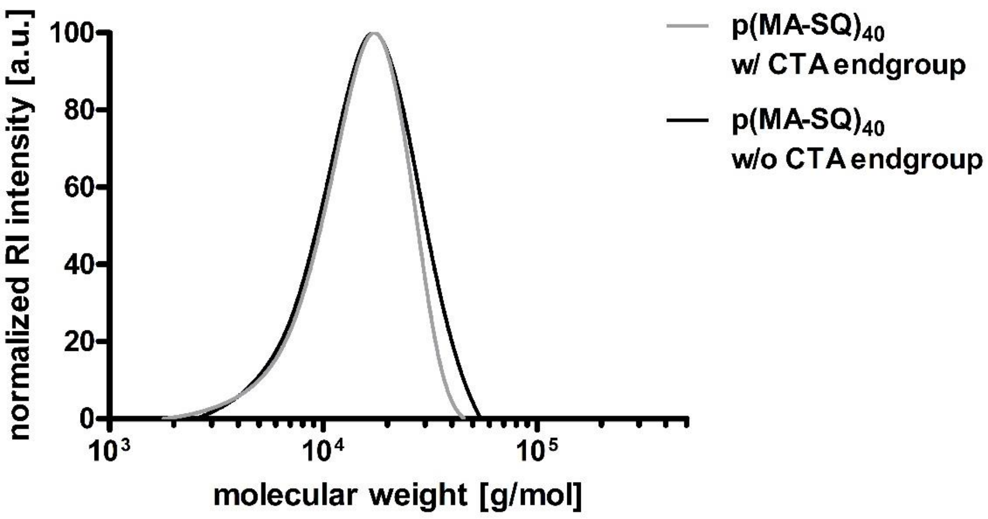

Figure S 33: Molecular weight distributions of homopolymer p(SQ-MA)$_{40}$ before and after RAFT end group removal determined by SEC (DMAc, PMMA-Std).

3.5 Reaction kinetics of RAFT homopolymerization

To investigate the kinetics of the RAFT homopolymerization of MA-SQ, samples of the reaction mixture were taken at different time points. After 0, 90, 180, and 320 min 15 µL reaction mixture was taken, while the reaction mixture stayed under inert Argon atmosphere. Samples were diluted immediately with DMSO-d_6 and exposed to air, before ^{1}H NMR measurements were performed. Monomer conversion was determined by the ratio between the integrals of the polymer (CH_2-*NH*-C- at 8.7 and 8.6 ppm; -O-CH_2-CH_3 at 4.7 ppm, grey) to integrals of the monomer (CH_2=C- at 5.6 and 5.3 ppm, orange).

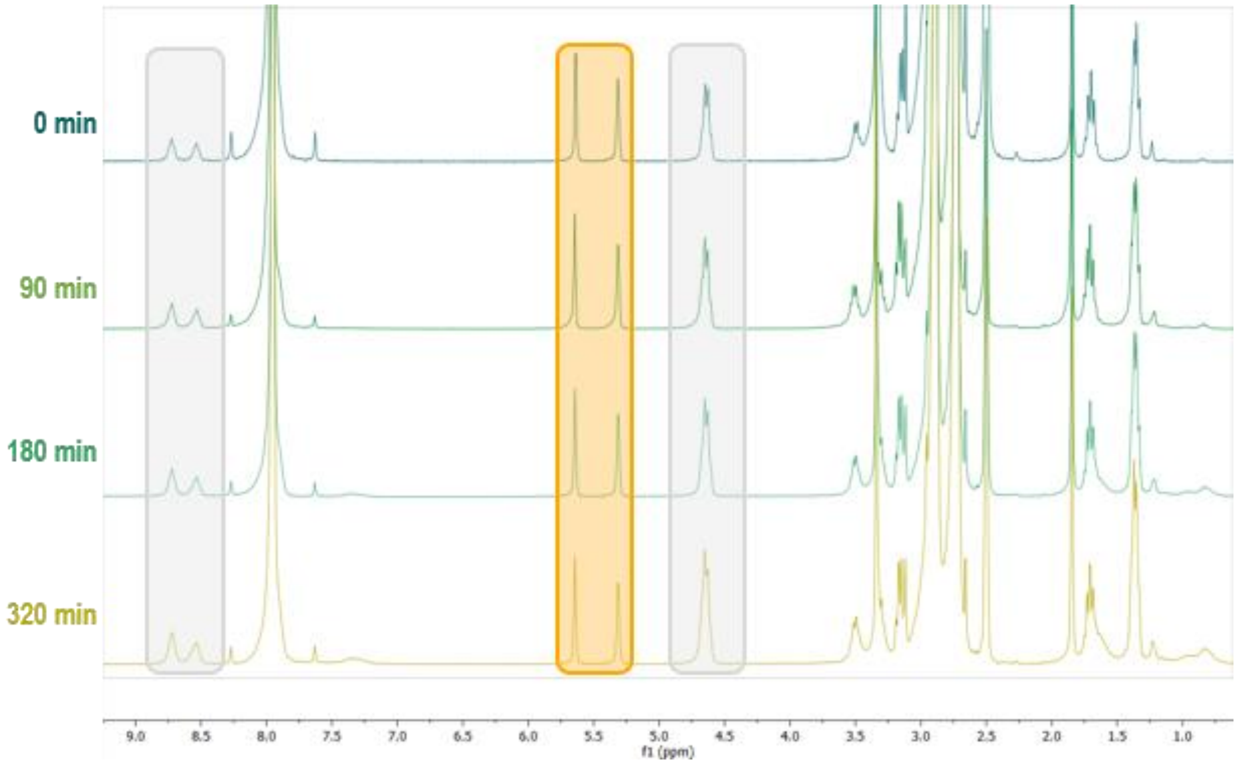

Figure S 34: ^{1}H NMR spectra (300 MHz) of reaction mixture of p(MA-SQ) after 0, 90, 180, and 320 min in DMSO-d_6.

A

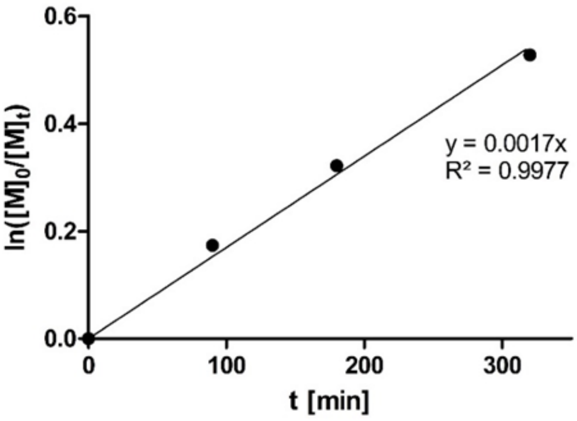

B

t [min]	conv. [%]	[M]	ln([M]$_0$/[M]$_t$)
0	0	0.744	0
90	16	0.625	0.174
180	27,5	0.539	0.322
320	41	0.439	0.528

Figure S 35: (A) Monomer conversion (ln([M]$_0$/[M]$_t$)) over reaction time, (B) Corresponding monomer conversion (conv.), monomer concentration ([M]), and (ln([M]$_0$/[M]$_t$)) over time.

3.6 Polymer-analogous reaction of homopolymer by amidation with primary, secondary, or tertiary amines

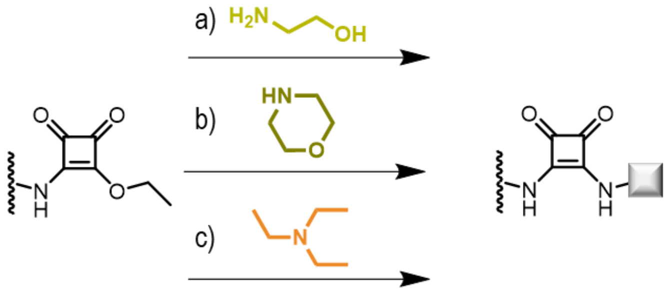

Figure S 36: Schematic reaction of pendant squaric ester amide moieties of homopolymer p(MA-SQ)$_{40}$ with a) aminoethanol, b) morpholine or c) triethylamine (TEA).

The reaction kinetics of the pendant squaric ester amide moieties of p(MA-SQ)$_{40}$ with primary, secondary, or tertiary amines were monitored by UV/Vis absorbance. Therefore, 25 µg homopolymer (25 µL of a 1.00 mg/mL stock solution in DMSO, 0.09 µmol, 1 eq.) was diluted with 1975 µL DMSO (v:v, 1:80) yielding a final concentration of 12.5 µg/mL. To this solution an excess of aminoethanol (5.4 µL, 90.00 µmol, 1000 eq.), morpholine (7.8 µL, 90.00 µmol, 1000 eq.), or TEA (12.6 µL, 90.00 µmol, 1000 eq.) was added, respectively. UV/Vis spectra were recorded regularly over 2.5 h.

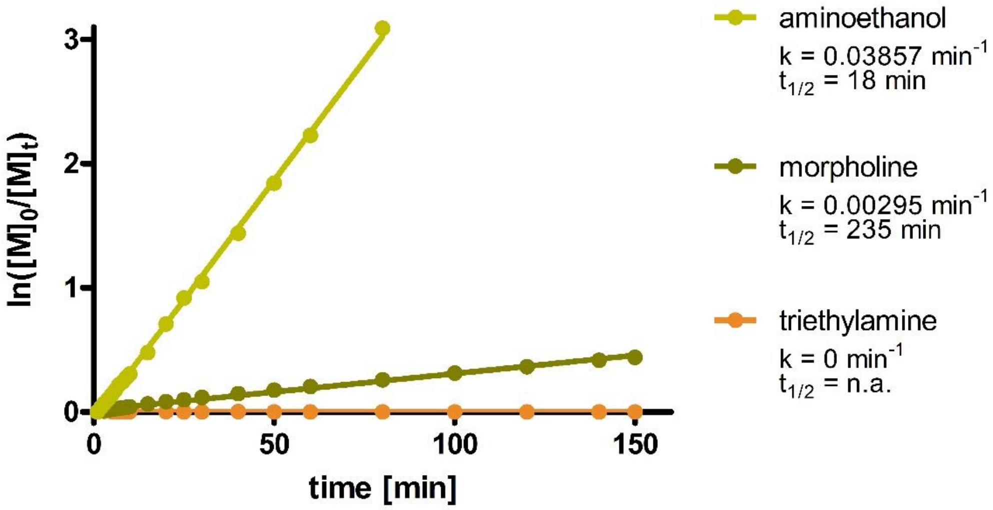

Figure S 37: ln ([M]$_0$/([M]$_t$) over time. Under the assumption the reaction followed - due to the vast excess of amine - first order kinetics, the reaction half time (t$_{½}$, time needed for half of the squaric ester amides to react with the amines) and the reaction rate constant k are given.

The same reaction could also be conducted on preparative scale using 20 mg homopolymer (72.6 µmol, 1 eq.) and only a slight excess of aminoethanol (21.7 µL, 0.36 mmol, 5.0 eq), morpholine (31.6 µL, 0.36 mmol, 5.0 eq), or TEA (50.5 µL, 0.36 mmol, 5.0 eq) was added. The reaction was conducted in 0.7 mL of DMSO-d$_6$ to record ^{1}H-NMR spectra before and after addition of the amines. After the reaction time the modified homopolymers were isolated by three-fold precipitation in cold diethyl ether (-20 °C) and dried under high vacuum. Subsequently, ^{1}H NMR and SEC analysis of the purified samples were performed.

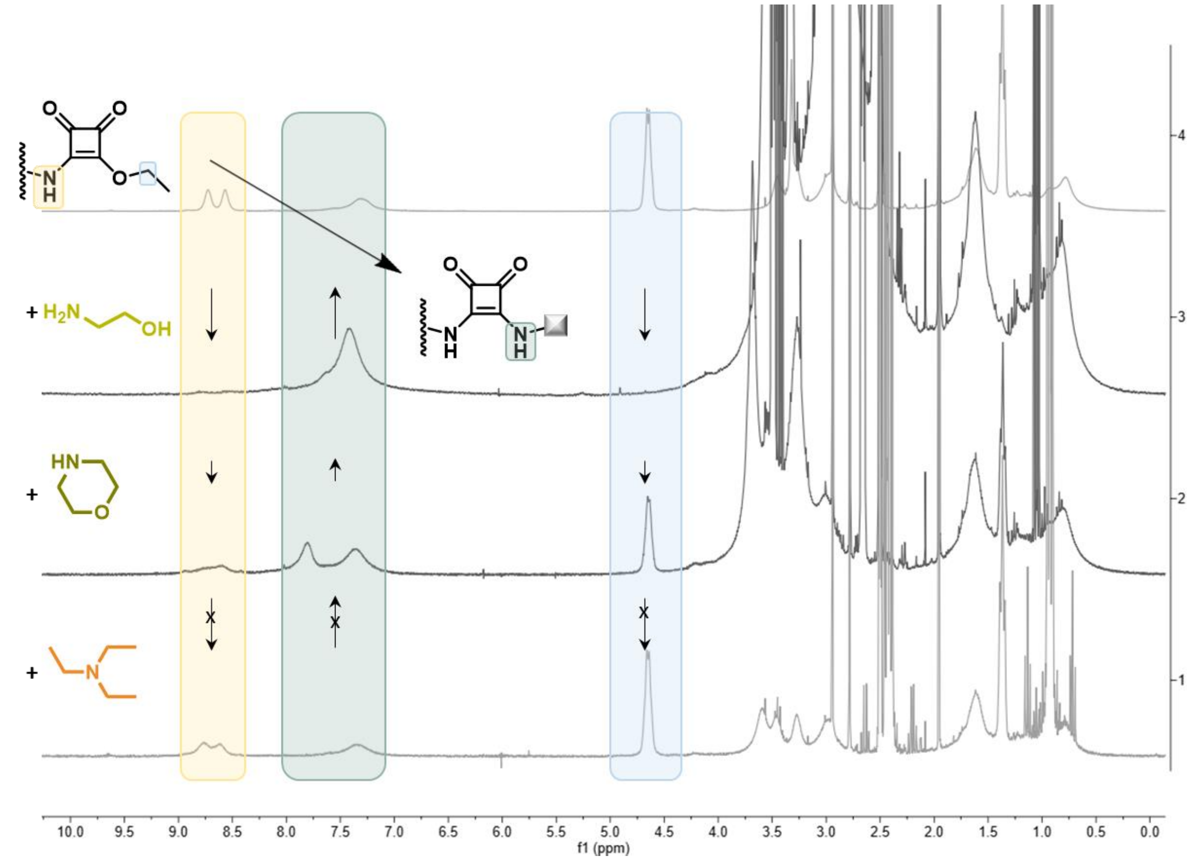

Figure S 38: ¹H NMR spectra of p(MA-SQ)₄₀ in DMSO-d₆ and the corresponding reaction mixtures 2.5 h after addition of primary amine aminoethanol, secondary amine morpholine, or tertiary amine TEA.

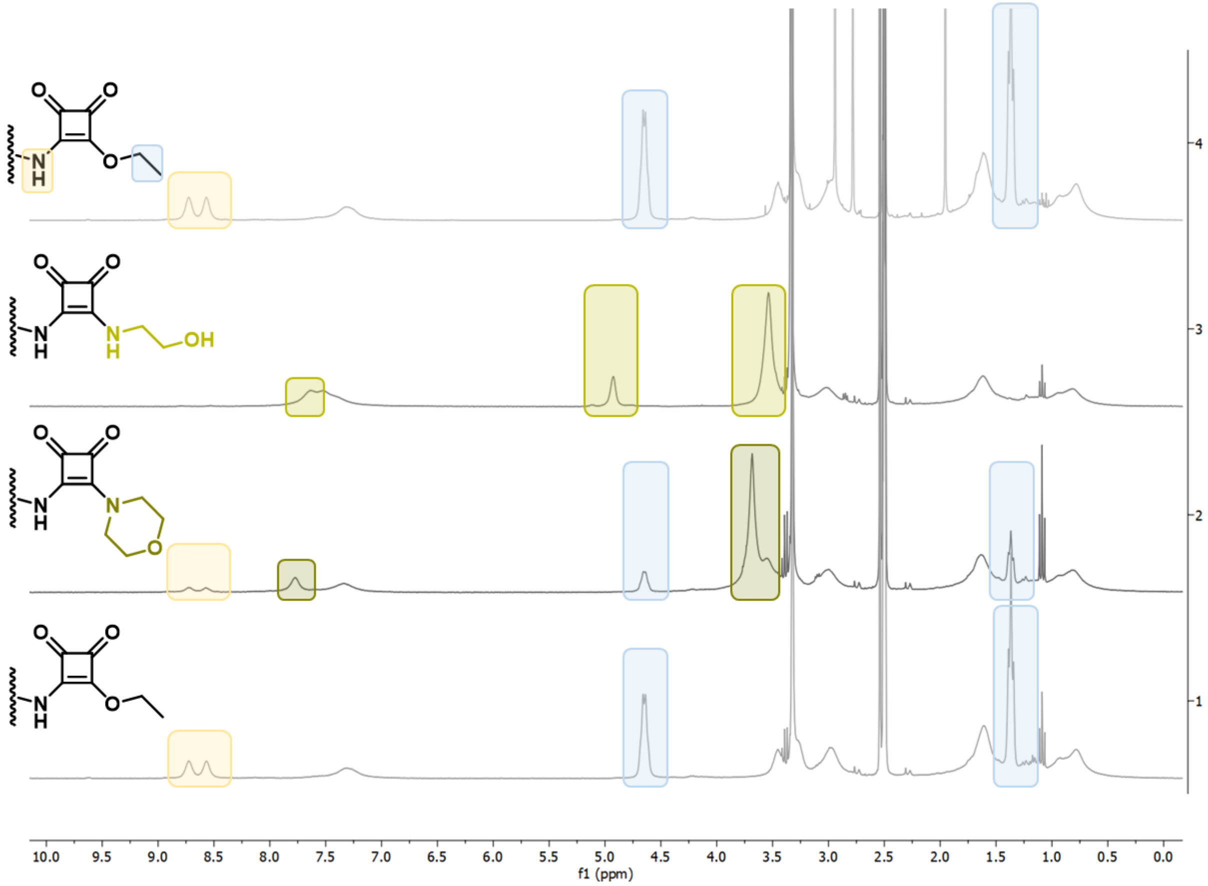

Figure S 39: ¹H NMR spectra of p(MA-SQ)₄₀ in DMSO-d₆ and purified p(MA-SQ)₄₀ after modification with primary amine aminoethanol, secondary amine morpholine, or tertiary amine TEA.

To investigate whether the reaction with the secondary amine morpholine could reach full conversion the polymer-analogous reaction of the homopolymer with morpholine was repeated with an increased reaction time of 22 h at an elevated temperature of 70 °C.

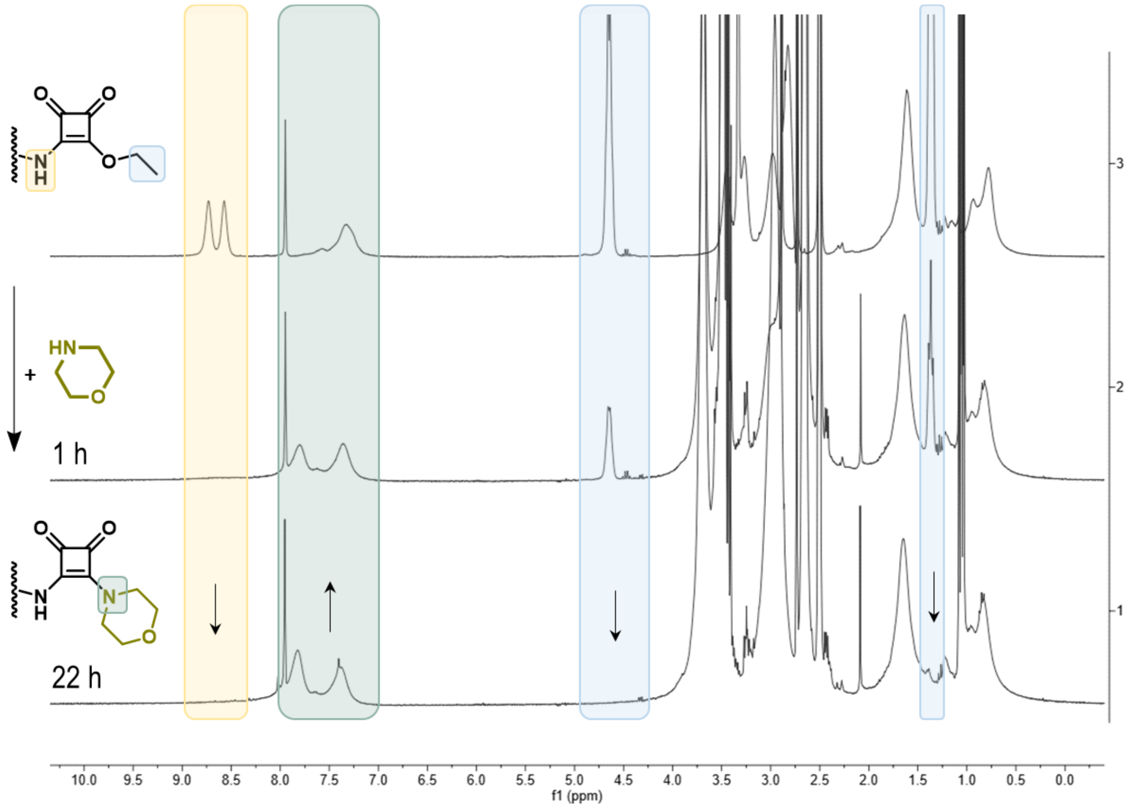

Figure S 40: ^{1}H NMR spectra of p(MA-SQ)$_{25}$ in DMSO-d$_{6}$ and the corresponding reaction mixtures 1 and 22 h after addition of the secondary amine morpholine at 70 °C.

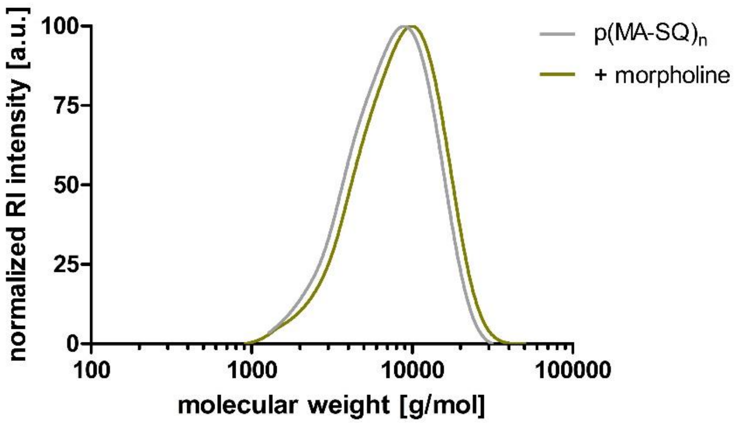

Figure S 41: SEC molecular weight distribution of p(MA-SQ)$_{25}$ before and after conversion with morpholine.

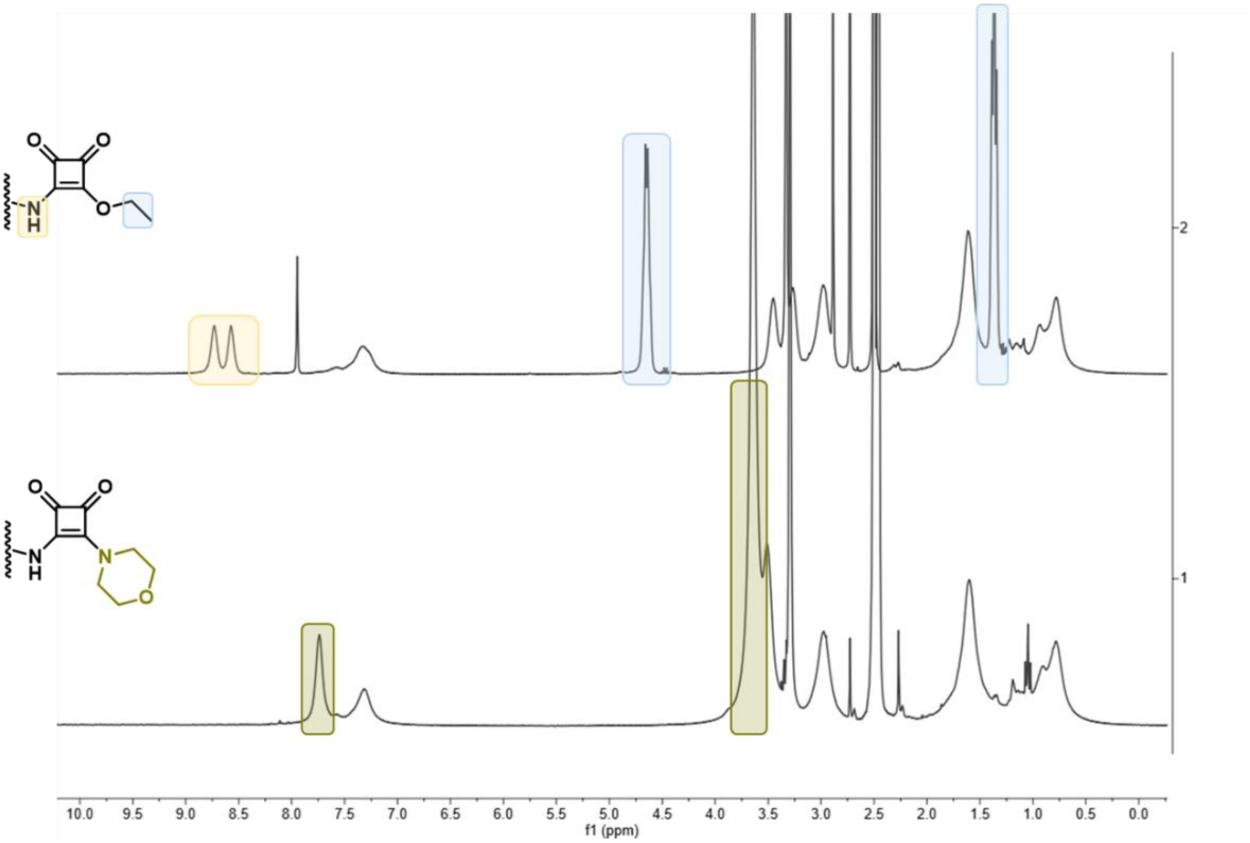

Figure S 42 ^1H NMR spectra of p(MA-SQ)$_{25}$ in DMSO-d$_6$ and purified p(MA-SQ)$_{25}$ after modification with secondary amine morpholine.

3.7 Block copolymer-analogous reaction of self-assembled micelles by amidation with primary amines

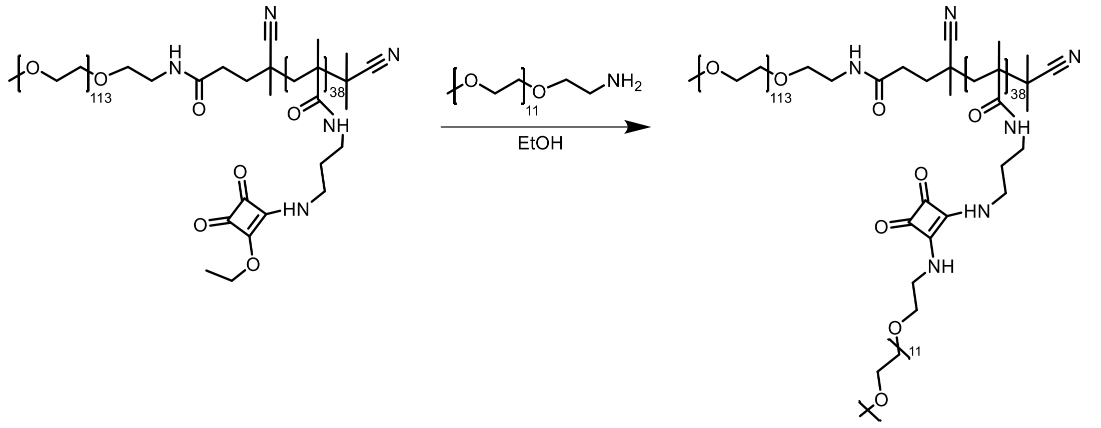

Figure S 43: Schematic reaction of self-assembled squaric ester amide block copolymer micelles mPEG$_{113}$-b-p(MA-SQ)$_{38}$ with PEG$_{11}$-amine.

In a DLS cuvette 1 mg of micelles mPEG$_{113}$-b-p(MA-SQ)$_{38}$ dispersed in 200 µL of ethanol (5.0 mg/mL, 2.56 µmol amine reactive units, 1.0 eq.) were treated with PEG$_{11}$-amine (9.6 mg, 12.8 µmol, 5.0 eq.) and monitored over time by repeated DLS measurements. A gradual particle unfolding and micelle disassembly could be observed both by mean volume mean as well as scattering count rate, and after 30 min all micelles disappeared.

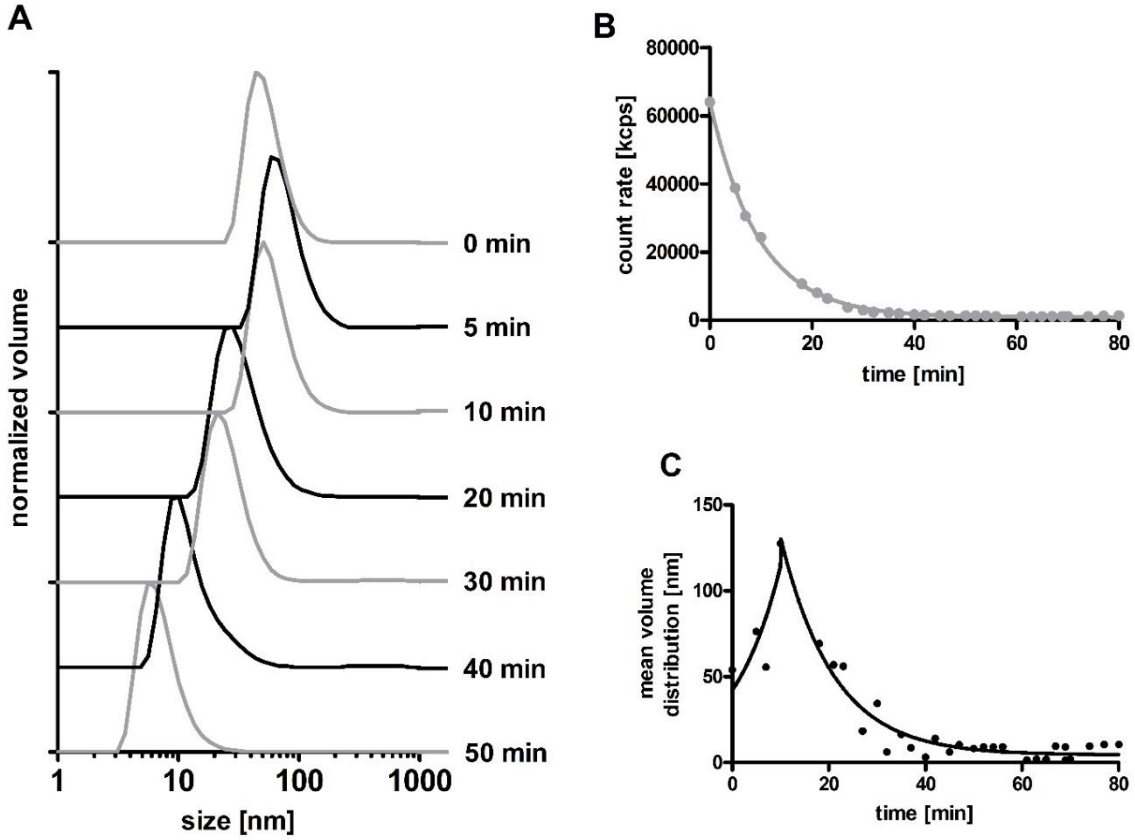

Figure S 44:DLS analysis of the aminolysis and micelle unfolding of the self-assembled squaric ester amide block copolymer micelles mPEG$_{113}$-b-p(MA-SQ)$_{38}$ with PEG$_{11}$-amine. (A) Particle size distribution, (B) scattering count rate, and (C) mean volume distribution

4. Nanogel Fabrication

4.1 Preparation of nanogels

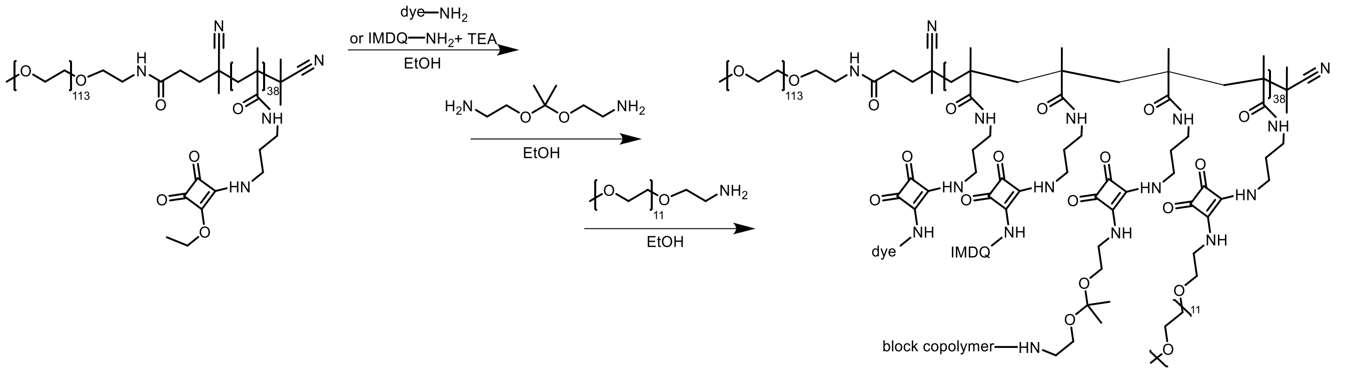

Figure S 45: Scheme of sequential preparation of nanogels

The block copolymer mPEG$_{113}$-p(SQ-MA)$_{38}$ (47.0 mg, 2.98 µmol polymer, 113.11 µmol reactive squaric ester amide units) was dispersed in ethanol (4.7 mL, 10 mg/mL). Ultrasonication of this dispersion for 1 h resulted in the self-assembly of polymeric micelles, which was verified by subsequent DLS measurements. For fluorescent labeling of the nanogels 113 µL Oregon Green (OG) or 117 µL tetramethylrhodamine (TMR) stock solution (2.5 mg/mL in DMSO, 0.57 µmol, 0.005 eq.) or 433 µL 800RS stock solution (2.5 mg/mL in DMSO, 1.14 µmol, 0.01 eq.) was added to the clear micellar dispersion and stirred for two days. For covalent drug loading of the nanogels with the immune modulator IMDQ, 489.1 µL of IMDQ stock solution (10 mg/mL in ethanol, 11.31 µmol, 0.10 eq.) was added to the micellar dispersion as well. After addition of 47.3 µL TEA (339.33 µmol, 3.00 eq.), the reaction mixture was allowed to stir at RT for 2 days.

The obtained dye-labeled IMDQ-conjugated or native dispersion was used to prepare three different kinds of nanogels (ketal-crosslinked, ether-crosslinked, and non-crosslinked). Thus, the dispersion was divided into three, wherefore 1.5 mL (15.0 mg, 0.95 µmol polymer, 36.2 µmol reactive squaric ester amide units) each was transferred into a Schlenk tube equipped with stirring bar. The different crosslinkers were added to the dispersions, whereby the added equivalents were adjusted in reference to the amine-reactive squaric ester amide units. 2,2,bis(aminoethoxy)propane (0.9 µL 5.4 µmol, 0.15 eq.) and 1,2-bis(aminoethoxy)ethane (0.8 µL 5.4 µmol, 0.15 eq.) were added as ketal- or ether-crosslinker. For the formulation of non-crosslinked nanogels 2-aminoethanol (0.7 µL 10.8 µmol, 0.30 eq.) was added. The reaction mixtures were allowed to stir at RT for one day. For complete conversion of the reactive squaric ester amide moiety, an excess of mPEG-NH_2 (M_n 0.75 kDa) (814 µL of 100 mg/mL in DMSO, 108.6 µmol, 3 eq.) was added to each reaction mixture, before they were allowed to stir for another five days. Complete conversion of the reactive ester units was confirmed by UV-Vis absorbance measurements. Subsequently, the reaction mixtures were dialyzed (MWCO: 1 kDa) against Millipore water containing 0.1 wt% NH_3 (1 L) for five days with water exchange twice per day. After lyophilization the nanogels were obtained as voluminous, yellowish powder (3x 21 mg, 63%), which could be easily re-dispersed in aqueous buffer at given concentrations.

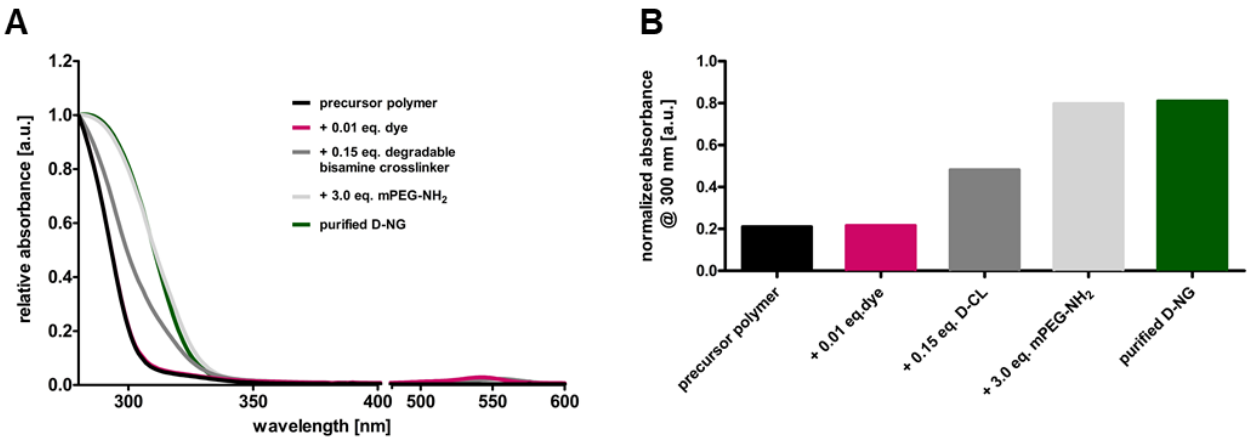

Figure S 46: Conversion of polymeric squaric ester amides to squaric bisamides during preparation of D-NG monitored by UV absorbance. (A) UV-Vis spectra of precursor polymer after step-wise nanogel preparation in ethanol compared to fully fabricated D-NG in PBS after purification, and (B) their corresponding absorbance at 300 nm.

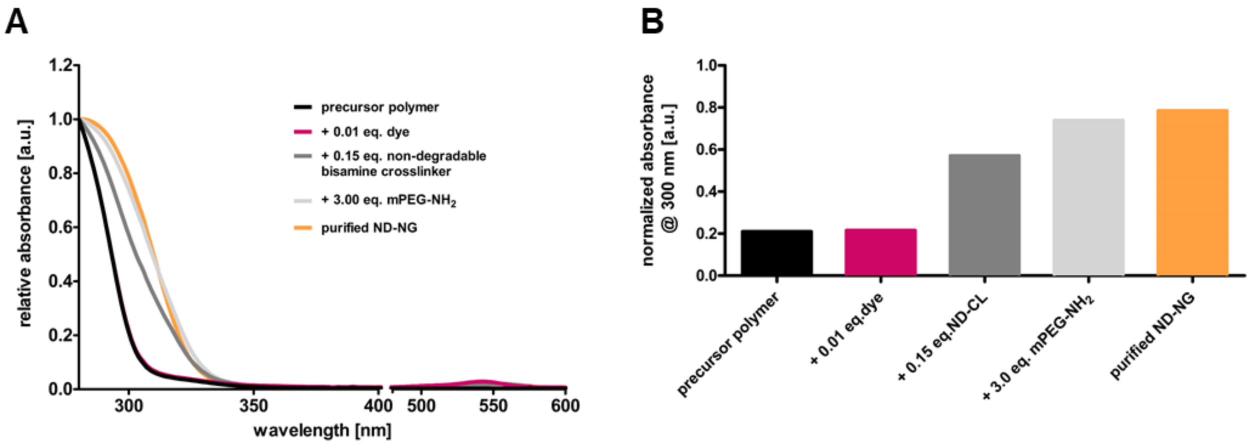

Figure S 47: Conversion of polymeric squaric ester amides to squaric bisamides during preparation of ND-NG monitored by UV absorbance. (A) UV-Vis spectra of precursor polymer after step-wise nanogel preparation in ethanol compared to fully fabricated ND-NG in PBS after purification, and (B) their corresponding absorbance at 300 nm.

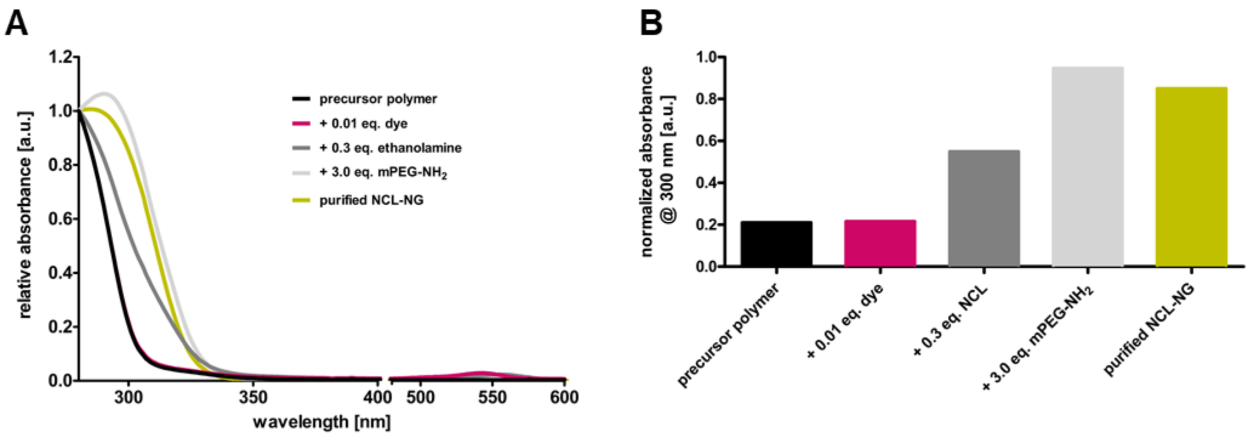

Figure S 48: Conversion of polymeric squaric ester amides to squaric bisamides during preparation of NCL-NG monitored by UV absorbance. (A) UV-Vis spectra of precursor polymer after step-wise nanogel preparation in ethanol compared to fully fabricated NCL-NG in PBS after purification, and (B) their corresponding absorbance at 300 nm.

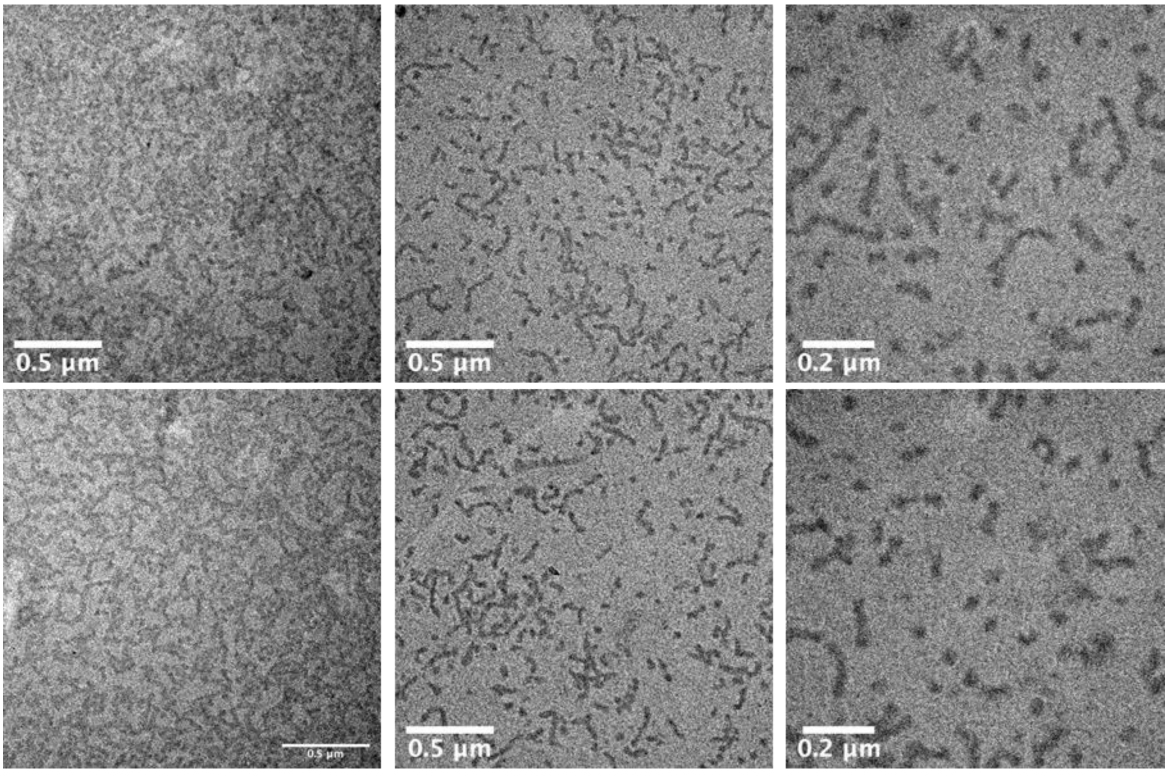

Figure S 49: TEM images of D-NG.

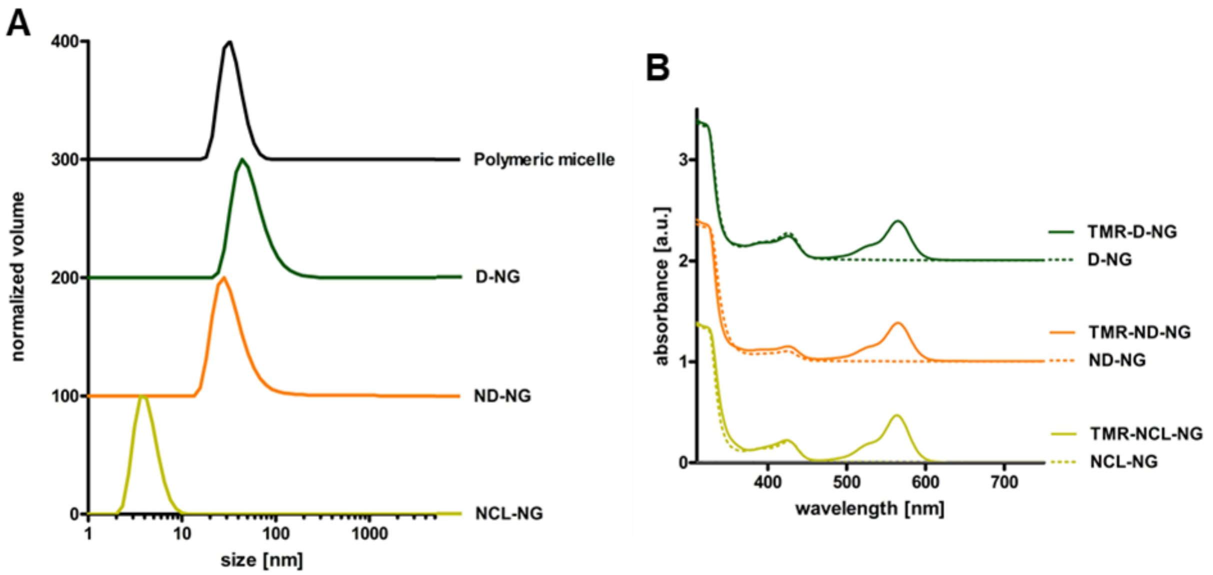

Figure S 50: Nanogel preparation in aqueous solution using sodium borate buffer. (A) DLS size distribution of polymeric precursor micelle, as well as of the corresponding fully fabricated and purified D-NG, ND-NG, and NCL-NG in aqueous solution. (B) UV Vis spectra of D-NG, ND-NG, and NCL-NG without and with fluorescent TMR-labeling indicating effective dye conjugation under aqueous conditions.

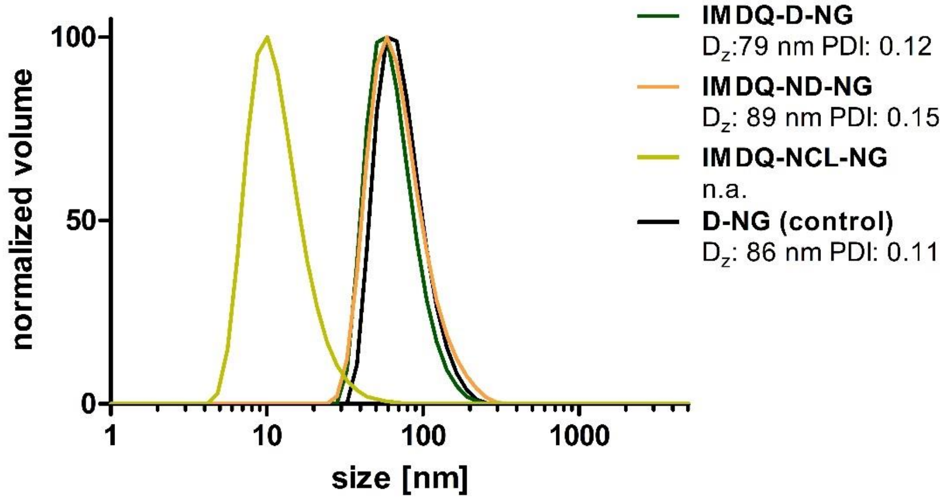

Figure S 51: DLS size distribution of fully fabricated nanogels IMDQ-D-NG, IMDQ-ND-NG, IMDQ-NCL-NG as well as empty ND-NG (control) in PBS. Note that NCL-NG disassemble upon preparation into fully soluble unimers.

4.2 IMDQ quantification via ^1H NMR analysis

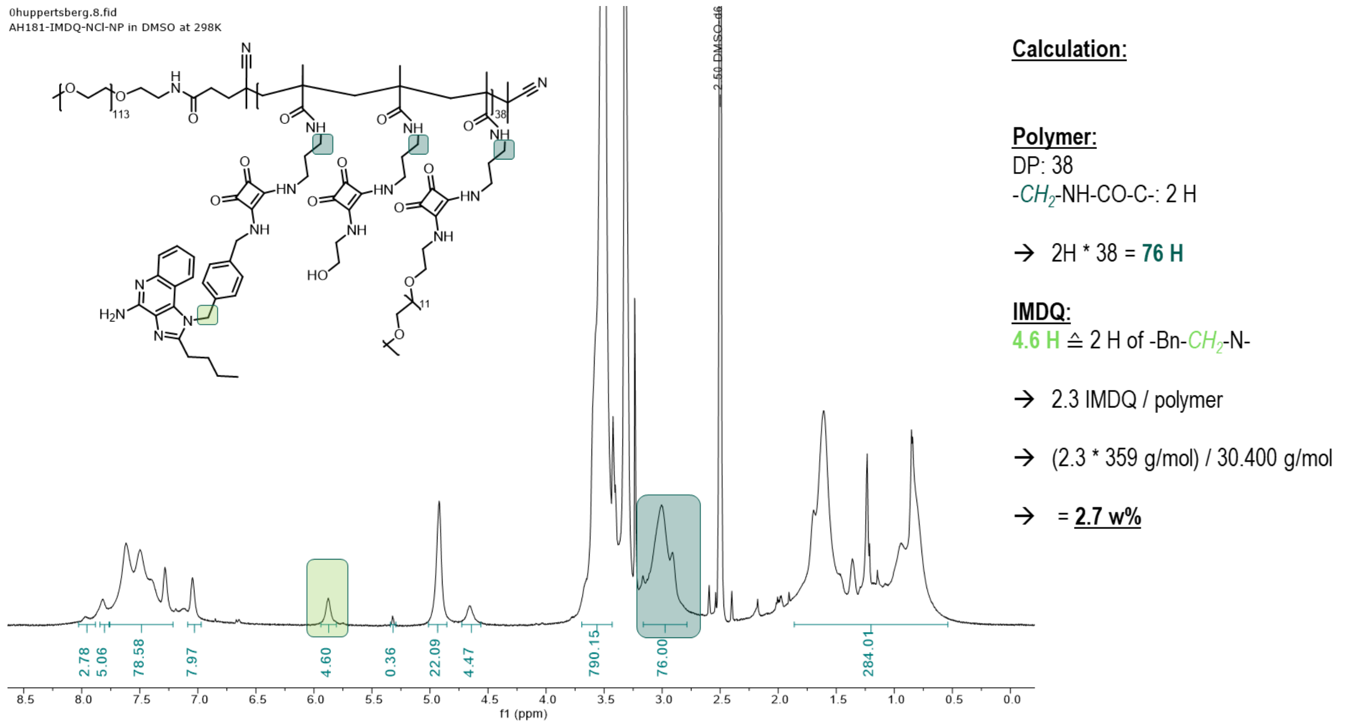

Figure S 52: ^1H NMR spectrum of IMDQ-NCL-NG in DMSO-d_6.

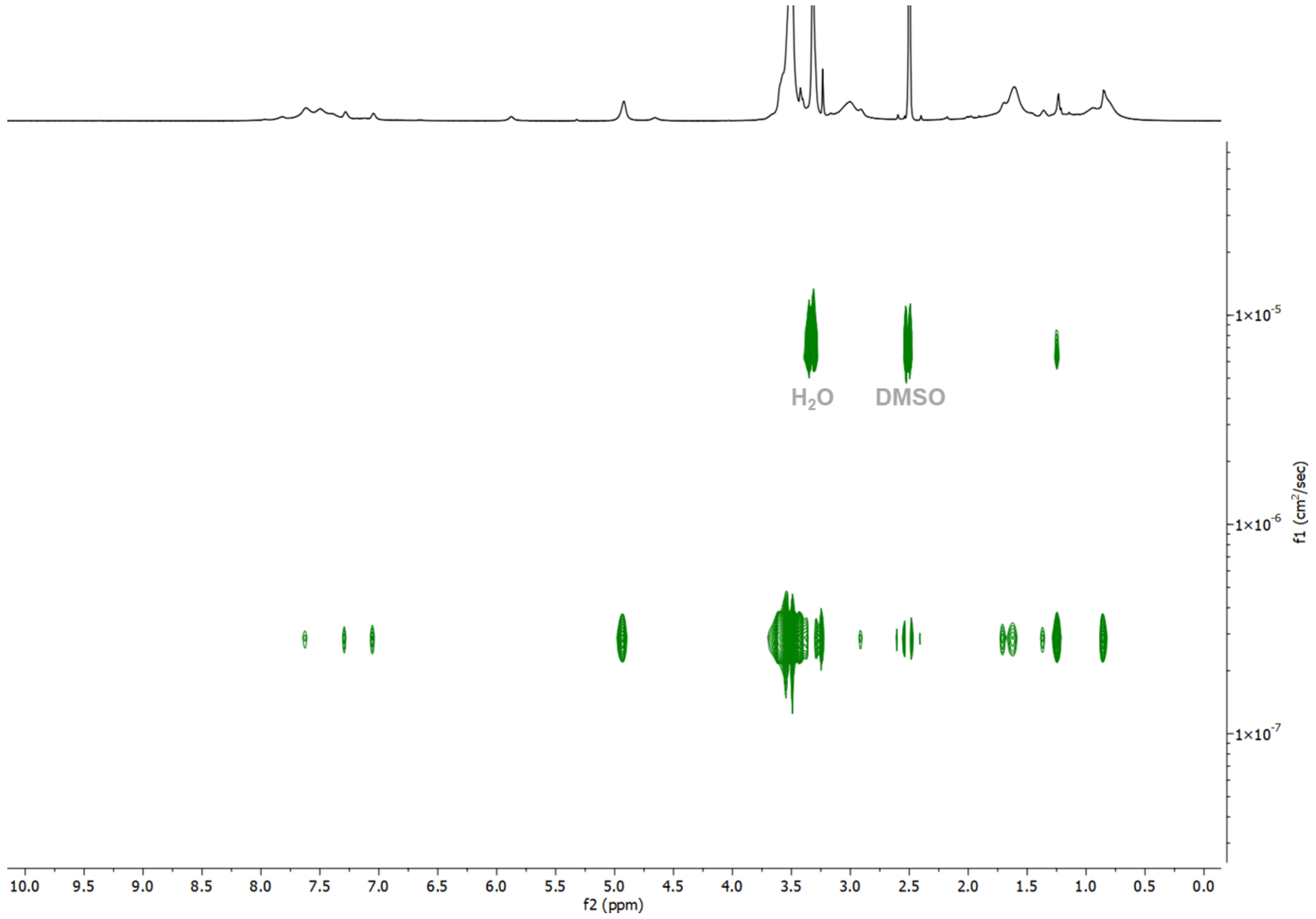

Figure S 53: DOSY spectrum of IMDQ-NCL-NG in DMSO-d_6.

4.3 Degradation/hydrolysis study of ketal-crosslinked nanogels

To investigate the degradability of the nanogels 50 mM acetate buffer (pH 5.2) was used. The lyophilized nanogels were re-dispersed directly in acetate buffer at 10.0 mg/mL followed by 5 min ultrasonication, subsequently DLS measurements were performed at 25 °C every 30 min for 24 h. To further evaluate stability at neutral pH nanogels were also re-dispersed in PBS and DLS measurements were conducted analogously.

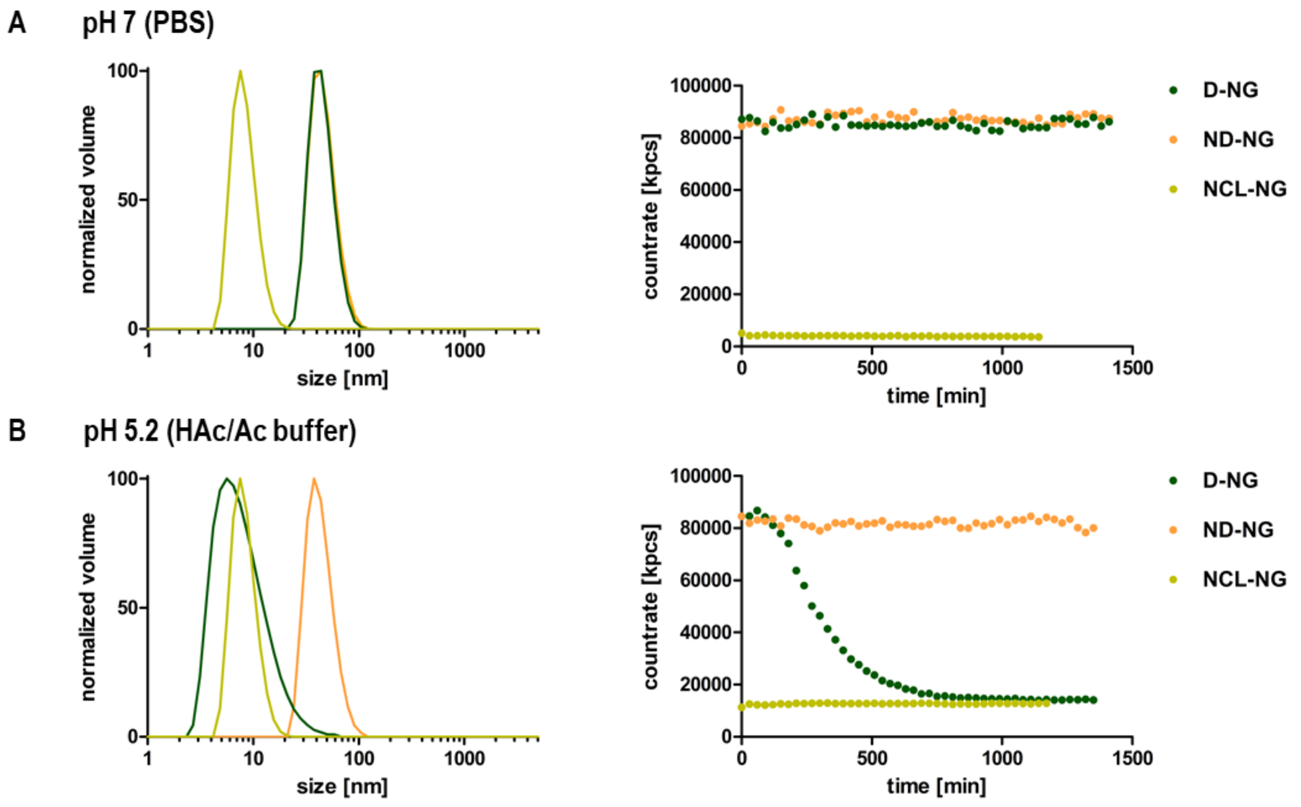

Figure S 54: DLS study of D-NG, ND-NG and NCL-NG (A) at neutral pH in PBS compared to (B) mildly acidic pH in HAc/Ac buffer. DLS count rate over time (left) and size distribution (right).

5. *In Vitro* Experiments

5.1 Cell culturing

RAW-Blue macrophages were cultured in Dulbecco's modified Eagle's medium (DMEM) supplemented with 10% fetal bovine serum, 1% penicillin/ streptomycin, 2 mM L-glutamine, 1 mM sodium pyruvate, and 0.01% zeocin at 37 °C with 5% CO_2 saturation.

5.2 Cell uptake experiment in RAW Blue-macrophages using confocal fluorescence microscopy

RAW-Blue macrophages were seeded on Willco-Dish glass bottom dishes (250 000 cells in 950 µL of culture medium) and incubated overnight. Next, cells were pulsed with 50 µL of a 2 mg/mL tetramethylrhodamine cadaverine-labeled nanogels (D-NG or ND-NG) or polymer solution (NCL-NG) in PBS (yielding a total nanogel/polymer concentration of 100 µg/mL) followed by incubation for 4 h and 24 h. Afterwards, culture medium was aspirated, and cells were washed two times with PBS. Next, 500 µL of 4% paraformaldehyde was added and allowed to fixate for 30 min at 37 °C. A staining solution was prepared by adding cholera toxin B (CTB)- AF488 (10 µL of a 1 mg/mL stock in PBS) and Hoechst 33258 (20 µL of a 1 mg/mL stock in PBS) to a PBS buffer containing 1% of BSA (2 mL). After aspiration and washing, 500 µL of this staining solution was added to the fixed cells and incubated for 30 min at 37 °C. Finally, cells were washed and stored in 1 mL PBS containing 1% of BSA. Confocal microscopy images were recorded by DMI6000 microscope purchased from Leica (Wetzlar, Germany) with a 1.40 NA 63x oil immersion objective, coupled to an Andor DSD2 scanner system. Images were processed by ImageJ software package.

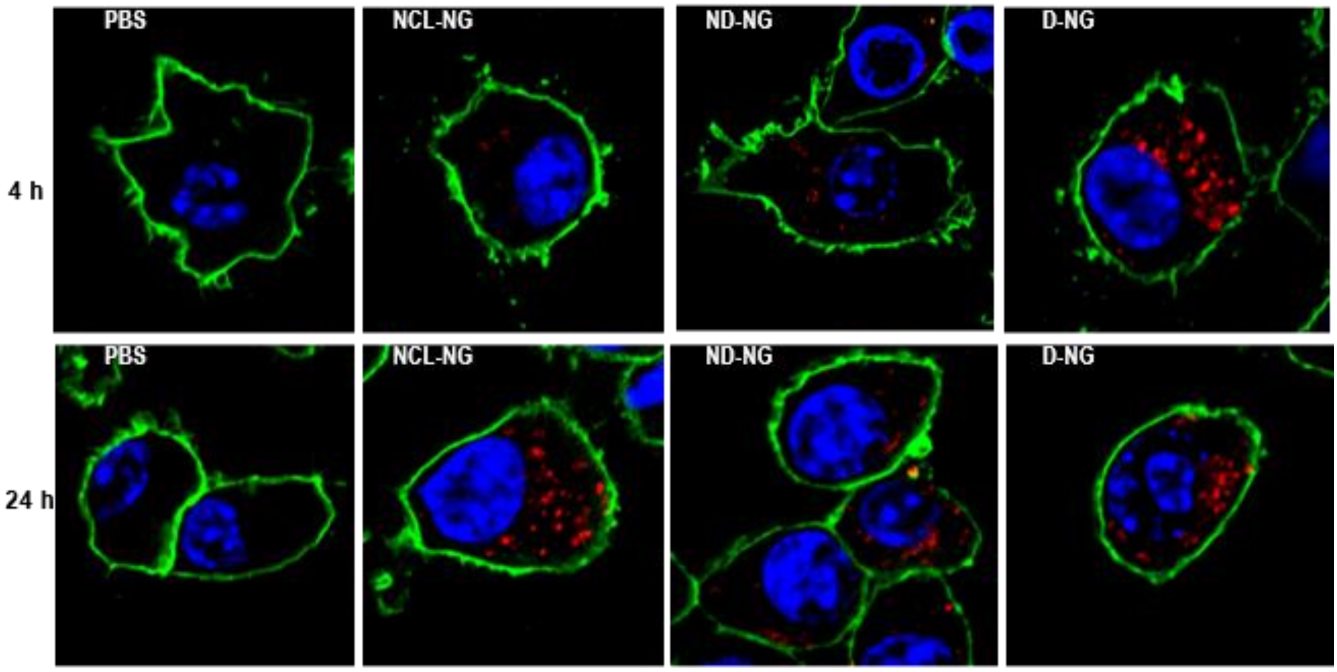

Figure S 55: Confocal microscopy images of RAW Blue macrophages incubated with PBS (control), NCL-NG, ND-NG, or D-NG at 100 µg/mL for 24 h (blue: nuclei stained with Hoechst 33258; green: cell membrane stained with cholera toxin B (CTB)- AF488; red: nanogel samples labeled with tetramethylrhodamine (TMR)).

5.3 Flow cytometry (FACS)

RAW-Blue macrophages were seeded into 24-well titer plates (250 000 cells per well in 950 µL of culture medium) and incubated overnight to allow cell sedimentation and subsequent adhesion to the bottom of the wells. Next, cells were incubated with 50 µL of tetramethylrhodamine-labeled nanogel (D-NG and ND-NG) or polymer solution (NCL-NG) in PBS (yielding a total nanogel/polymer concentration of 10 µg/mL, 50 µg/mL or 100 µg/mL). All samples were run in triplicate (n = 3) and the experiment was conducted for 24 h at 37°C. Cell culture medium was then aspirated, and cells were washed with 1 mL of PBS and incubated with 250 µL of Cell dissociation buffer (15 min, 37°C). The cell suspensions were then diluted with 750 µL of cell culture medium, transferred into Eppendorf tubes and centrifuged immediately (350*g*, 10 min, 5°C). Finally, the supernatant was aspirated, and the cell pellets were suspended in 250 µL of PBS. They were kept on ice to maintain cell integrity prior to flow cytometric analysis performed on a BD Accuri C6 (BD Biosciences). The data was processed by FlowJo software.

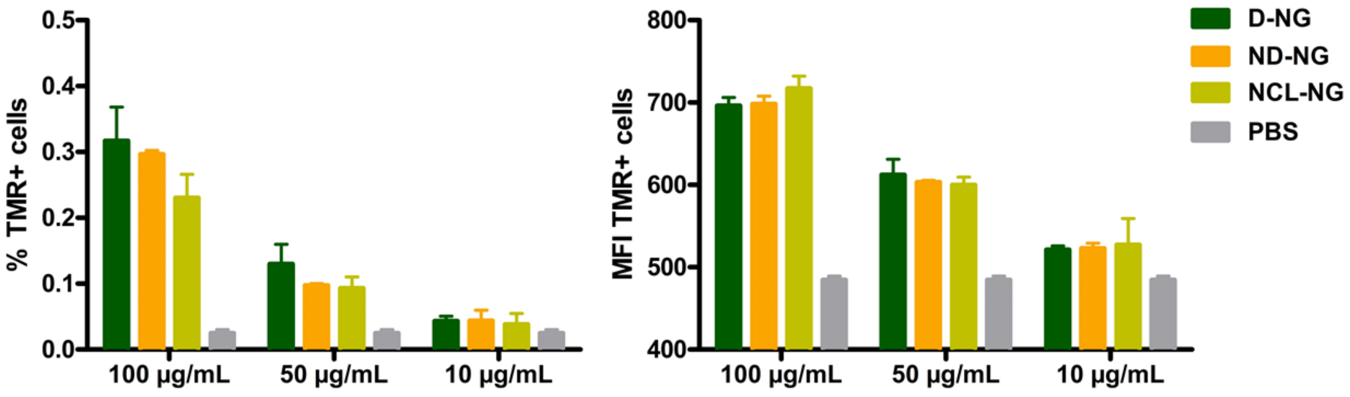

Figure S 56: Flow cytometry analysis revealing percentage of cells positive for tetramethylrhodamine (%TMR+ cells) and mean cellular fluorescence intensity (MFI) of RAW Blue macrophages incubated with tetramethylrhodamine-labeled D-NG, ND-NG, NCL-NG at 100, 50, and 10 µg/mL or PBS as control for 24 h.

5.4 Cellular metabolic activity of RAW-Blue macrophages using MTT assay

Cellular metabolic activity as indicator of cell viability, proliferation and cytotoxicity was determined after incubation of RAW-Blue Macrophages with IMDQ-loaded nanogels using a colorimetric MTT assay. Each well containing RAW-Blue macrophages was treated with IMDQ-loaded or empty nanogels at given concentrations. After 24 h 25 µL of 3-(4,5-dimethylthiazol-2-yl)-2,5-diphenyltetrazolium bromide (MTT, 2 mg/mL in PBS) was added, respectively, before the macrophages were incubated at 37 °C for 1.5 h. To dissolve the formed formazan crystals, 100 µL of 10% SDS (m/v)/0.01 M HCl was added to each well followed by incubation at 37 °C overnight. Cellular metabolic activity was determined in relation to positive (PBS blank, 100% activity) and negative (10% DMSO, 0% activity) control samples based on absorbance measurements at 570 nm. Experiments were performed in quadruplicate (n = 4).

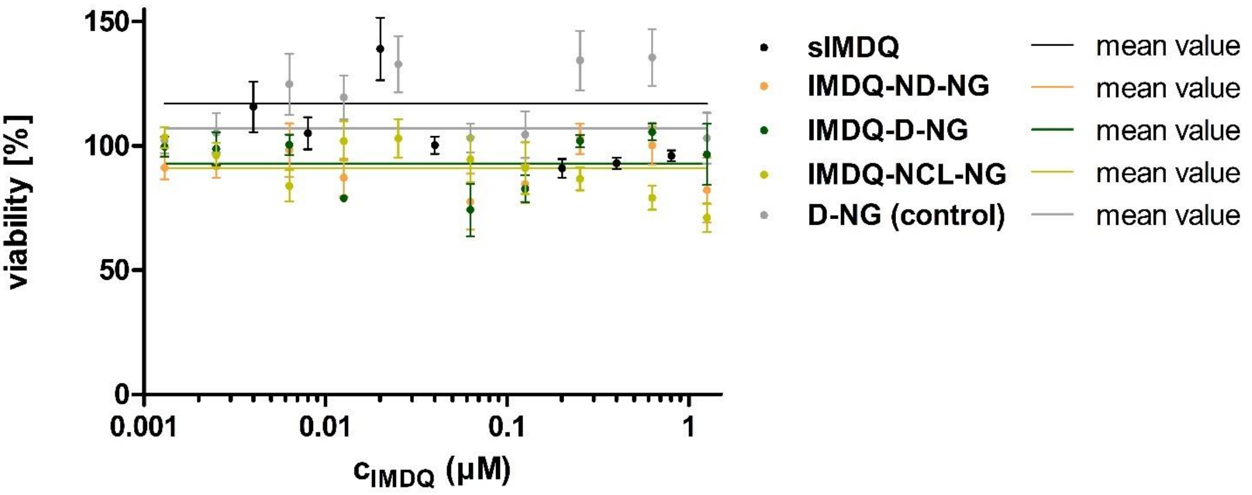

Figure S 57: MTT Assay indicating cell viability of RAW-Blue macrophages treated with sIMDQ, IMDQ-D-NG, IMDQ-ND-NG, IMDQ-NCL-NG and D-NG (control) (n = 4).

5.5 RAW-Blue macrophage TLR reporter assay

To compare the immune-modulatory properties of prepared IMDQ-loaded nanogels with the free drug (sIMDQ) *in vitro* TLR receptor stimulation was analyzed by RAW-Blue Macrophage reporter assay. Therefore, NF-κB/AP-1 activation following TLR receptor stimulation was detected by secreted embryonic alkaline phosphatase (SEAP) as proposed by the manufacturer InvivoGen. RAW-Blue cells were seeded into 96-well plates (90,0000 cells/well in 180 µL culture medium) and allowed to adhere overnight. The next day, 20 µL of IMDQ-loaded nanogel or control samples (sIMDQ, empty NG) was added to each well at given final concentrations. After 24 h or 48 h incubation at 37 °C, 50 µL supernatant of each well was collected and transferred into another 96-well plate. Subsequently, levels of SEAP were quantified by QUANTI-Blue Assay. After addition of 150 µL QUANTI-Blue solution, samples were incubated at 37 °C for 2 h. Detection of optical density at 620 nm with a plate reader determined SEAP levels. An increase in optical density relative to the negative control (PBS treated) declared TLR receptor stimulation. All experiments were performed in quadruplicate (n=4).

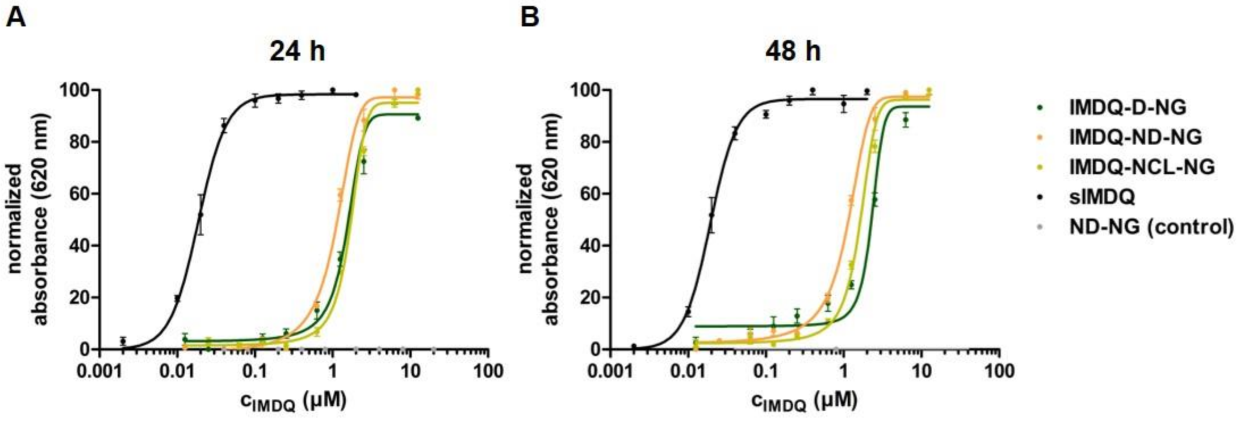

Figure S 58: RAW-Blue Assay indicating TLR stimulation of RAW-Blue macrophages incubated (A) for 24 h or (B) for 48 h with sIMDQ, IMDQ-D-NG, IMDQ-ND-NG, IMDQ-NCL-NG and ND-NG (control) (n = 4).

6. *In Vivo* Experiments

6.1 Biodistribution imaging of 800RS-labeled nanogels

The conducted animal study followed an approval by the local ethics committee on animal care (number 23 177-07/G 17-1-030, Government of Rhineland Palatinate, Germany). 8–10 week-old female BALB/c mice (body weight ~20–25 g) were obtained from Charles River (Sulzfeld, Germany) and kept according to the guidelines of the institute and the local government (12 h light-dark cycles at 25 °C and 40–60% humidity with humane care, access to regular chow and water ad libitum). To investigate the biodistribution of the nanogels 100 µL of 800RS-labeled nanogels (2 mg/mL in PBS) were injected intravenously into Balb/c mice (n = 3, two mice treated with PBS served as control). Prior to injection mice were anesthetized with isoflurane gas and treated *via* tail vein injection with the nanogels. After 24 h, mice were again anesthetized with isoflurane gas and transferred into an IVIS (*in vivo* imaging spectrum) system (Caliper Life Sciences, Hopkinton, MA, USA) for *in vivo* NIR fluorescence imaging. Picture integration time of the fluorescence source was set to 4 s. Recording parameters were set for excitation at 745 nm and emission at 800 nm to visualize the near infrared dye RS800 labeled nanogels. Afterwards, mice were sacrificed, and organs were harvested and put again into the imaging chamber for *ex vivo* signal quantification using Live Image software from PerkinElmer (Waltham, MA, USA). Organs were carefully circumscribed by region-of-interests (ROIs), and then their fluorescence was quantified as the average of area-normalized radiant efficiency.

6.2 Bioactivity imaging of IMDQ-loaded nanogels

To study the immune-stimulating effect of IMDQ-loaded nanogels, heterozygous BALB/c IFN-β (IFN-$\beta^{+/\Delta\beta\text{-}luc}$) reporter mice in the range of 7−9 weeks were housed in individual ventilated cages and given ad libitum access to food and water. The mice were subjected to intravenous injection of 5 µg IMDQ (soluble or bound to nanogel in 100 µL of PBS) *via* tail vein injection (n = 3, a mouse treated with PBS served as control). For subsequent *in vivo* bioactivity imaging, mice were injected subcutaneously with 200 µL D-luciferin (15 mg/ mL, Gold Biotechnology, USA) at the given time points (0, 4, 24, and 48 h post injection), and *in vivo* luminescence imaging was recorded 12 min later using the IVIS Lumina II imaging system from PerkinElmer, (Waltham, MA, USA). Photon flux was analyzed by Living Image 4.4 software from Caliper life sciences (Hopkinton, MA, USA).

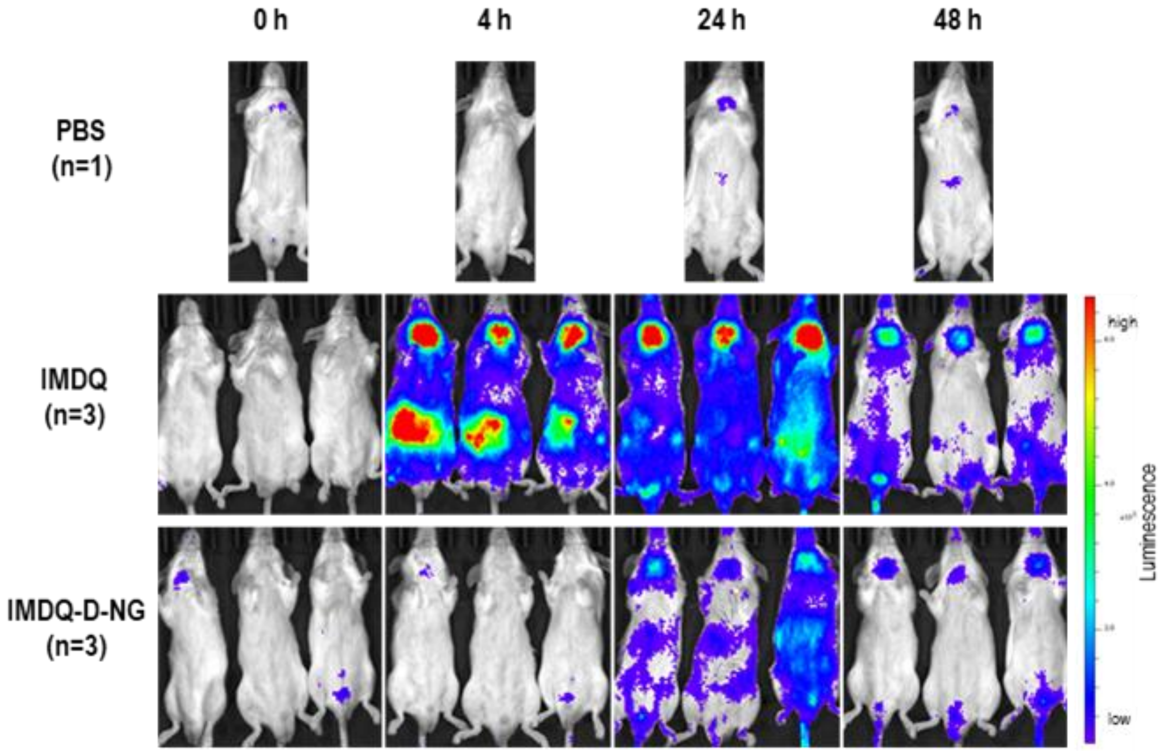

Figure S 59: In vivo bioactivity of soluble and nanogel bound IMDQ (IMDQ and IMDQ-D-NG) after intravenous. injection into heterozygous BALB/c IFN-β (IFN-$\beta^{+/\Delta\beta\text{-luc}}$) reporter mice (n = 3).

6.3 Flow cytometry analysis of splenic innate immune activation

Heterozygous BALB/c IFN-β (IFN-$\beta^{+/\Delta\beta\text{-luc}}$) reporter mice were intravenously injected with 5 µg IMDQ (soluble or bound to nanogel in 100 µL of PBS, n = 3) were sacrificed after 48 h and spleens where isolated for flow cytometry analysis. A single cell suspension was prepared form the dissected organ for analysis by flow cytometry. Isolated splenocytes were collected in ice cold PBS, smashed through 70 µm cell strainers, washed with PBS and stained for 30min at 4°C with a live-dead stain and the following primary labeled monoclonal antibodies: CD3e, CD11c, CD11b, CD19, CD69, CD80, CD86, F4-80, Ly-6G, and MHC-II. 123count ebeads were added to determine cellularity prior to acquiring them on by flow cytometry (BD FACS Aria). Analyses were done using the FlowJo software package according to the following gating procedure.

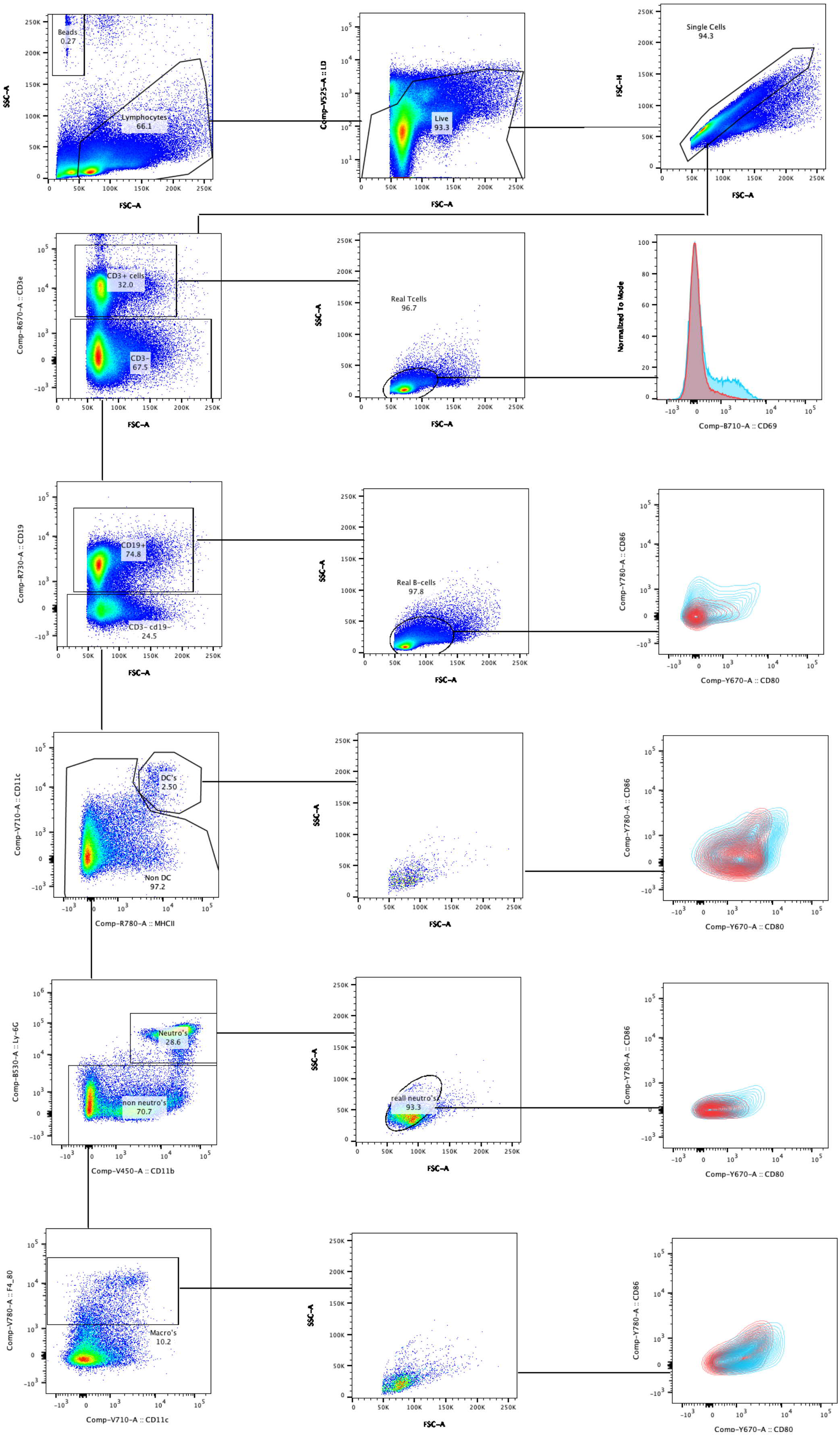

Figure S 60: Gating procedure for flow cytometry analysis of splenic innate immune activation.

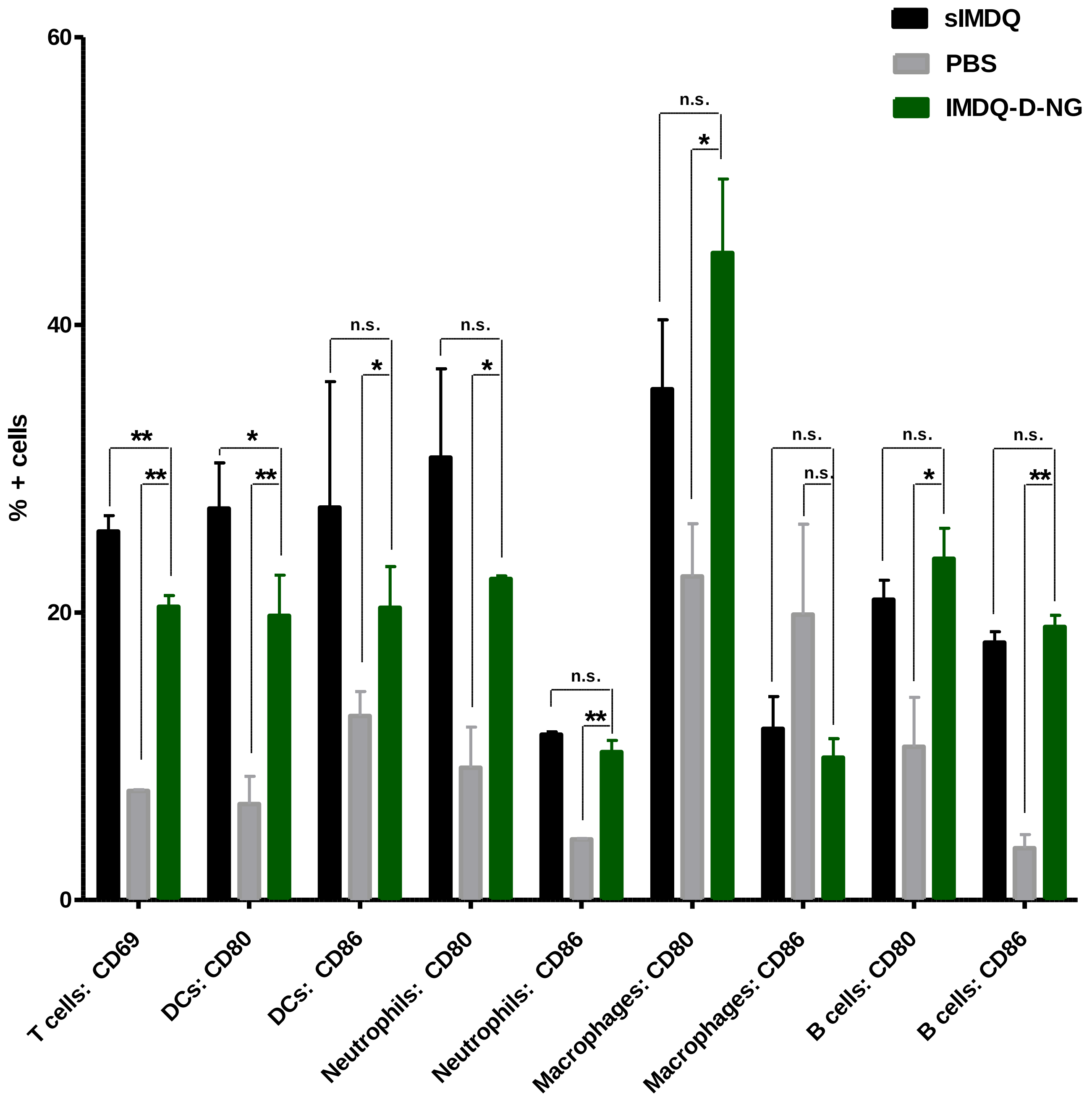

Figure S 61: Percentage of immune activation marker positive cells in the respective immune cell subpopulation (CD69 for T cells and CD80/CD86 for DCs, neutrophils, macrophages, and B cells) according to the gating procedure above.

6.4 Acute toxicity studies

To investigate acute *in vivo* toxicity BALB/c mice were intravenously injected with 5 µg of soluble or nanogel bound IMDQ (n = 4, 100 µL of sIMDQ (0.05 mg/mL in PBS) or IMDQ-D-NG (2 mg/mL in PBS), PBS served as control). After 4 or 24 h, mice were sacrificed and blood was retrieved by heart puncture to determine the serum contents for aspartate aminotransferase (AST), alanine aminotransferase (ALT), glutamate hydrogenase (GLDH), lipase (LIP), and blood urea by nitrogen content (BUN). The parameters were measured by the central laboratory of the University Medical Center of Mainz. The remaining sera were diluted 1:4 and stored at -20°C. Cytokine concentrations for TNF-α, IFN-γ and IL-6 were determined by cytometric bead assay (CBA; BD Biosciences, San Jose, CA) as recommended by the manufacturer. For that purpose, bead populations with distinct fluorescence intensities were conjugated with cytokine-specific capture antibodies. Recombinant cytokines were used to prepare standard dilutions. Samples were mixed with capture beads, subsequently incubated with PE-conjugated detection antibodies for 1 h (at room temperature in the dark), and subjected to flow cytometric analysis. Results were analyzed using FCAP Array Analysis Software v.1.0.1 (BD Biosciences).

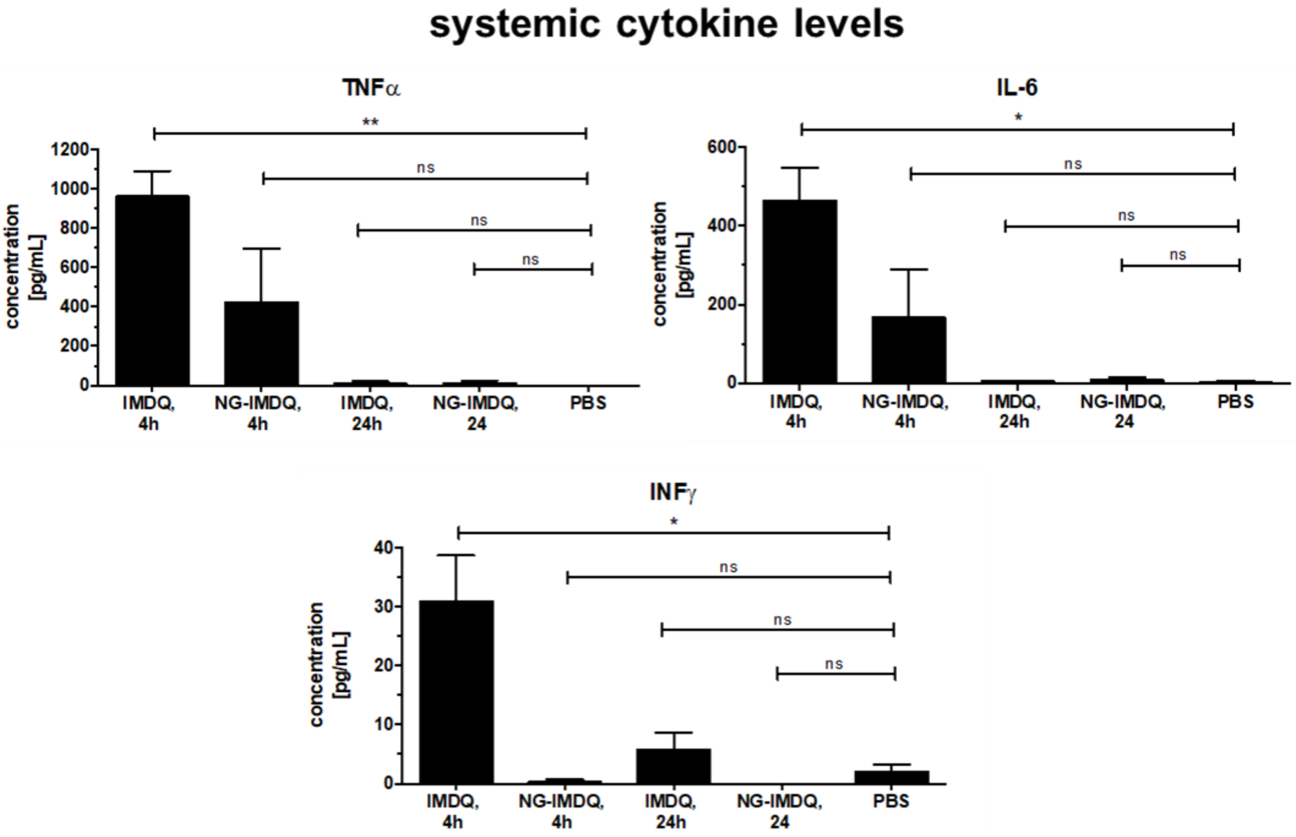

Figure S 62: Serum cytokine levels of mice treated with 5 µg soluble or nanogel bound IMDQ (n = 4).

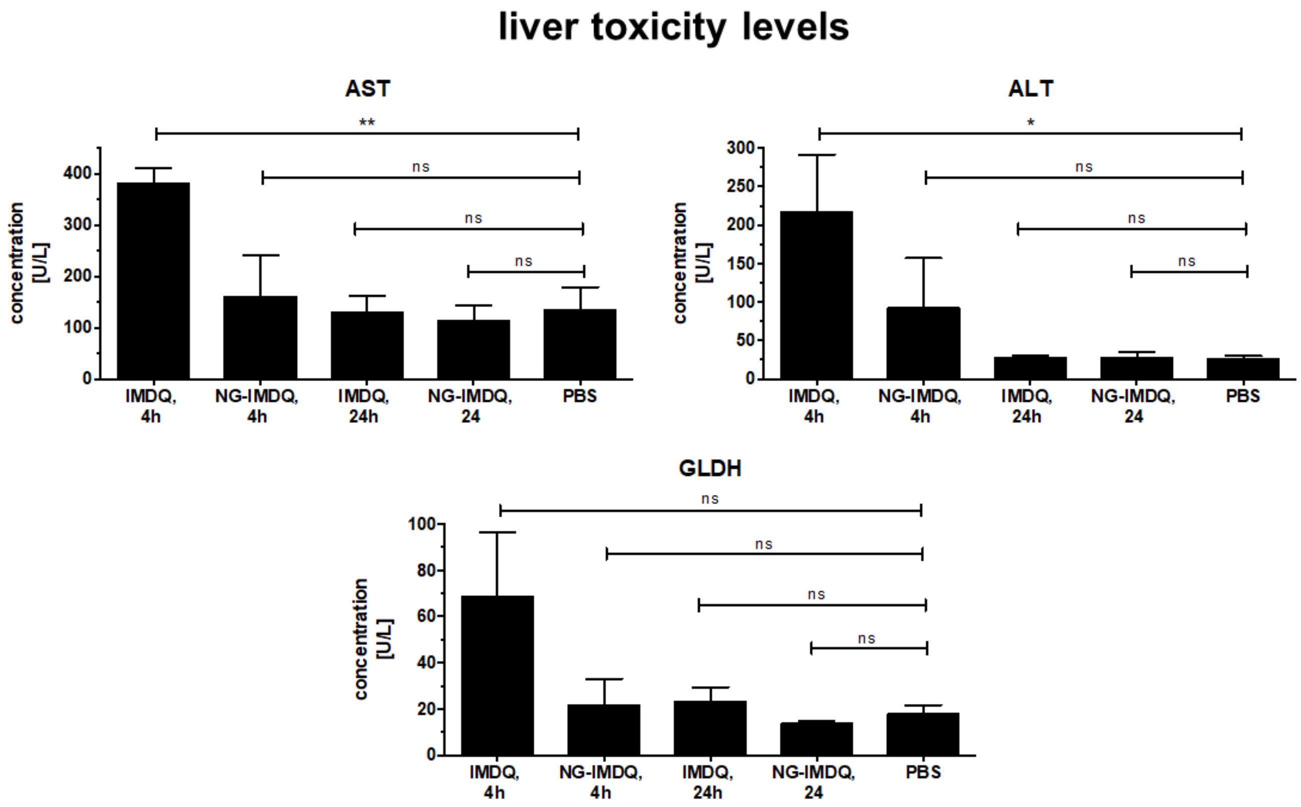

Figure S 63: Liver enzyme serum activities of mice treated with 5 µg soluble or nanogel bound IMDQ (n = 4).

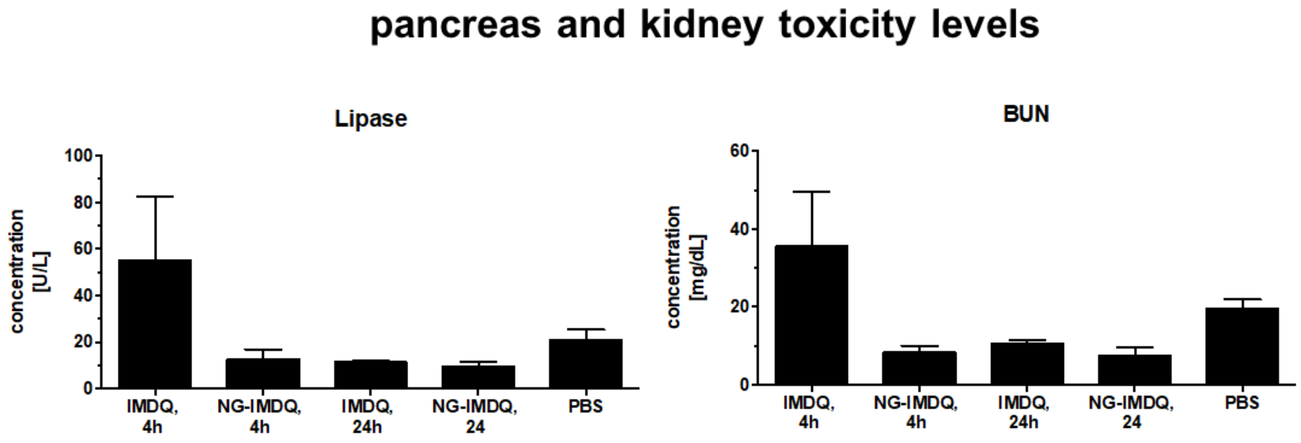

Figure S 64: Pancreas lipase serum activity and kidney toxicity related blood urea nitrogen (BUN) concentration of mice treated with 5 µg soluble or nanogel bound IMDQ (n = 4).

7. Statistical analysis

Data are shown as mean values + standard deviation. To calculate statistical significance of the mean values, unpaired Student's t-test with Welch's correction was performed using Graph Pad Prism 8 software (*: $p \leq 0.05$; **: $p \leq 0.01$; ***: $p \leq 0.001$).
